# Pleiotropic prodrugs for both symptomatic and disease-modifying treatment of Alzheimer’s disease

**DOI:** 10.1016/j.apsb.2025.07.005

**Published:** 2025-07-07

**Authors:** Anže Meden, Neža Žnidaršič, Damijan Knez, Yuanyuan Wang, Ziwei Xu, Huajing Yang, Weiting Zhang, Anja Pišlar, Andrej Perdih, Simona Kranjc Brezar, Neža Grgurevič, Stane Pajk, Haopeng Sun, Stanislav Gobec

**Affiliations:** a University of Ljubljana, Faculty of Pharmacy, Ljubljana SI-1000, Slovenia; b University of Ljubljana, Faculty of Veterinary Medicine, Ljubljana SI-1000, Slovenia; c China Pharmaceutical University, Nanjing 210038, China; d National Institute of Chemistry, Ljubljana SI-1000, Slovenia; e Department of Experimental Oncology, Institute of Oncology, Ljubljana SI-1000, Slovenia

**Keywords:** Pleiotropic prodrug, Alzheimer’s disease, Butyrylcholinesterase, *α*_2A_ adrenoreceptor, Disease-modifying treatment, *N*-Heterocyclic ureas, Carbamates, Neurodegenerative diseases

## Abstract

The inherent complexity of Alzheimer’s disease (AD) and failed clinical trials have spiked the interest in multifunctional ligands that target at least two key disease-associated macromolecules in AD pathology. Here we present a focused series of pleiotropic *N*-carbamoylazole prodrugs with dual mechanism of action. Pseudo-irreversible inhibition of the first therapeutic target, human butyrylcholinesterase (hBChE), enhances cholinergic transmission, and thereby provides symptomatic treatment, same as the standard therapeutics in use for AD. Simultaneously, this step also functions as a metabolic activation that liberates a nanomolar selective *α*_2_-adrenergic antagonist atipamezole, which blocks pathological amyloid *β* (A*β*)-induced and noradrenaline-dependent activation of GSK3*β* that ultimately leads to hyperphosphorylation of tau, thus achieving a disease-modifying effect. Lead compound **8** demonstrated long-term pseudo-irreversible hBChE inhibition, metabolic activation in human plasma, blood–brain barrier permeability, and *p.o.* bioavailability in mice. Multi-day *in vivo* treatment with **8** in an A*β*-induced AD murine model revealed a significant alleviation of cognitive deficit that was comparable to rivastigmine, the current drug of choice for AD therapy. Furthermore, decreased GSK3*β* activation and lowered tau phosphorylation were observed in APP/PS1 mice. This surpasses the symptomatic-only treatment with cholinesterase inhibitors, as it directly blocks an essential pathological cascade in AD. Therefore, these multifunctional *α*_2_-adrenergic antagonists–butyrylcholinesterase inhibitors, exemplified by lead compound **8**, present an innovative, small molecule-based, disease-modifying approach to treatment of AD.

## Graphical abstract

Multifunctional *N*-carbamoylazole pleiotropic prodrugs disclosed here offer a novel treatment of Alzheimer’s disease. First, inhibition of butyrylcholinesterase (hBChE) leads to a symptomatic improvement of cognitive deficit. Second, and more importantly, a disease-modifying effect is achieved by blocking *α*_2A_ adrenoreceptor signalling, which in turn prevents hyperphosphorylation of tau.

**Figure:**
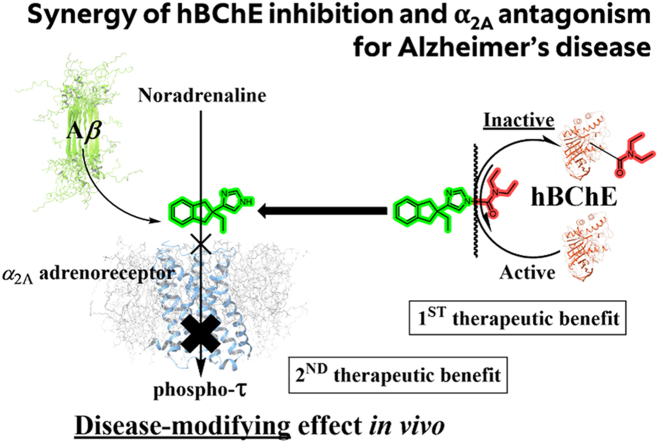

## 1 Introduction

Dementia, “a syndrome, usually of a chronic or progressive nature, that leads to deterioration in cognitive function beyond what might be expected from the usual consequences of biological ageing”, is the 7th leading cause of death, currently afflicting more than 55 million people worldwide^1^^,^^2^. The most common variant of dementia is Alzheimer’s disease (AD), a neurodegenerative disease, uniquely characterized by extracellular aggregates of amyloid *β* (A*β*) and intracellular neurofibrillary tangles of hyperphosphorylated protein tau (ptau). Until three monoclonal antibodies (aducanumab, lecanemab, and donanemab) have been approved in the last three years claiming disease-modifying treatment of AD^3^, ^4^, ^5^, ^6^, only symptomatic treatment was available, predominantly based on cholinesterase inhibitors (ChEIs). The latter stem from the so-called “cholinergic hypothesis of AD” and only temporarily improve cognition in AD patients^7^, ^8^, ^9^.

Human butyrylcholinesterase (hBChE) is the less known sibling of human acetylcholinesterase (hAChE) that performs various physiological roles besides halting the cholinergic neurotransmission, some of which are still unknown. BChE is a prospective drug target in neurodegenerative diseases, detoxification of cocaine, heroin, and other xenobiotics, nerve agent protection, ghrelin homeostasis, etc.^10^, ^11^, ^12^, ^13^ The rationale behind using selective BChEIs in AD is based on their lack of peripheral parasympathomimetic side effects, and the fact that in some brain regions BChE takes over as the main ChE as the disease progresses^14^.

The defect in the highly interconnected cholinergic and noradrenergic ascending neuromodulatory systems in neurodegenerative diseases has been recognised since 1980s, but the spotlight initially focused only on the cholinergic hypothesis^9^^,^^15^, ^16^, ^17^. Locus coeruleus (LC), the noradrenergic centre of the brain, is situated in the pons of the brainstem, providing multiple efferent projections across the central nervous system (CNS). LC-neurons are among the first to suffer from ptau accumulation during the prodromal phase of AD, leading to neuronal death and compensatory changes to preserve noradrenaline **1** levels, similar to what has been observed in the cholinergic system^18^^,^^19^. This noradrenergic dysfunction is hypothesized to cause or at least promote AD progression. In addition, it exacerbates neuroinflammation and impairs A*β* removal by microglia^20^. Noradrenaline's effects are mediated *via* nine human metabotropic adrenergic receptors, belonging to *α*_1_, *α*_2_, and *β* type. In general, noradrenaline loss results in cognitive dysfunction, while either agonism at *α*_1_ and *β* receptors, or *α*_2_ antagonism enhances memory and learning. Furthermore, there is some evidence that activation of *α*_2A_ and *β*_2_ adrenoreceptors stimulates A*β* production^18^^,^^21^, ^22^, ^23^, ^24^. Of the three highly homologous pre- and postsynaptic *α*_2_ receptor subtypes, *α*_2A_ and *α*_2C_ are mostly found in CNS, especially in LC and hippocampus, and modulate effects of noradrenaline. The *α*_2A_ subtype is mostly responsible for hypotensive, sedative, analgesic, and hypothermic effects^25^, ^26^, ^27^, ^28^.

**Figure 1:**
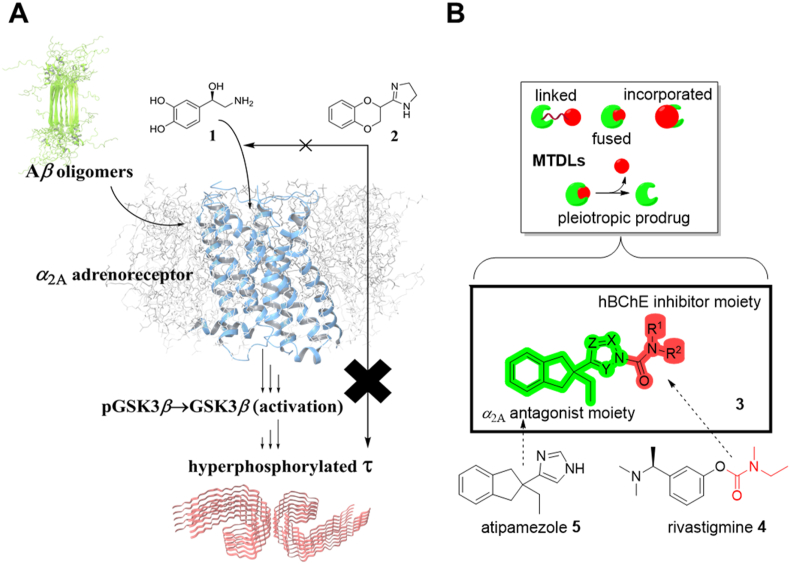
Rationale and design of multitarget-directed ligands presented in this study. (A) The pathological cascade that connects A*β* oligomers with neurofibrillary tangles of hyperphosphorylated tau. A*β* oligomers bind to a putative allosteric site on *α*_2A_ adrenoreceptor and amplify the noradrenaline (**1**)-mediated activation of GSK3*β* that in turn leads to ptau. This can be prevented altogether by administering an *α*_2_*orthosteric* antagonist, such as idazoxan **2**, which has been demonstrated *in vitro* and *in vivo*. (B) Different design strategies of multifunctional/multitarget-directed ligands (MTDLs) and the general structure of the pleiotropic prodrugs **3** described in this work with its carbamoyl warhead in red and the leaving group in green. Both pharmacophoric elements of **3** originate from two registered drugs, *O*-aryl carbamate rivastigmine **4** and *α*_2_ antagonist atipamezole **5**.

Importantly, Zhang et al.^29^ have shown that even in nanomolar concentrations, extracellular A*β* oligomers allosterically bind to the 3^rd^ extracellular loop of the *α*_2A_ adrenoreceptor and potentiate the noradrenaline-dependent activation of glycogen synthase kinase 3*β* (GSK3*β*) that leads to tau hyperphosphorylation. This cascade is blocked by administering *α*_2A_ adrenergic orthosteric antagonists (*e.g.*, idazoxan **2**) or GSK3*β* inhibitors (*e.g.*, LiCl) (Fig. 1A). This was also confirmed in two transgenic mice models, where long-term treatment with **2** reduced A*β* plaque load, GSK3*β* activation, microglial activation and neuroinflammation, tau hyperphosphorylation, and ameliorated cognitive deficits in cognitively-impaired mice with significant amyloid burden. Besides inhibiting GSK3*β*, these disease-modifying effects could also be attributed to the altered SorLA-dependent amyloid precursor protein (APP) trafficking. APP proteolysis with *β*-secretase, which is the first step leading towards A*β*, takes place in endosomes, whereas SorLA mediates the removal of APP from endosomes. This process is disrupted following the activation of *α*_2A_ adrenoreceptor^30^.

Late-onset sporadic AD is in its essence a multifactorial disease. Therefore, due to its inherent complexity, drug discovery efforts have shifted towards designing multifunctional or multi-target directed ligands (MTDLs) that act simultaneously on two or more prospective “targets” involved in the pathophysiology of AD. This multifunctionality is typically achieved through linking, fusing, or incorporating two or more molecular frameworks that each have activity of their own into a new, single molecular entity^31^, ^32^, ^33^, ^34^. In this study, rather than finding a molecule that would simultaneously act on two drug targets with disparate pharmacophoric requirements, we instead designed and synthesized *N*-carbamoylazoles/*N*-heterocyclic ureas exemplified by the general structure **3** (Fig. 1B, Supporting Information Scheme S1).

**Scheme 1:**
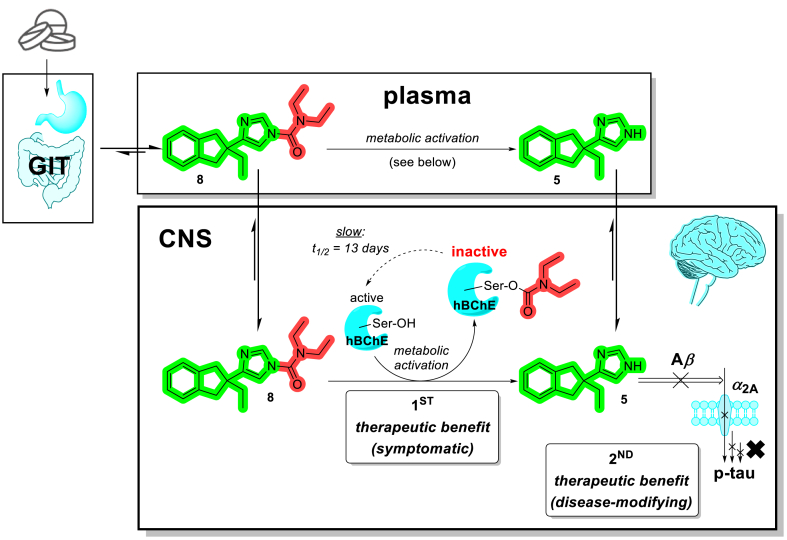
Dual mechanism of action—hBChE-mediated metabolic activation of **8** to the selective *α*_2_-adrenergic antagonist atipamezole **5** and their respective mechanisms of action.

*N*-Heterocyclic ureas, which have been reported as serine hydrolase inhibitors, are known to transfer their carbamoyl group to the catalytic serine residues, while their pyr-, tri-, tetra-, or imidazole moieties function as leaving groups^35^^,^^36^. Similarly, our *N*-carbamoylazoles **3** rely on the metabolic activation carried out by hBChE, which coincidently results in the pseudo-irreversible inhibition of this serine hydrolase by the carbamoyl warhead (Fig. 1B, in red—akin to the carbamate warhead in rivastigmine **4**, registered AD therapeutic), that is transferred to the catalytic Ser198. This process also liberates the leaving group, 4-(2-ethylindan-2-yl)-1*H*-imidazole **5** (INN name: atipamezole) or its analogues. **5** acts as an *α*_2_ antagonist (Fig. 1B in green) with orthogonal, but synergistic activity on the second target (*vide infra*—Scheme 1). Therefore, compounds of the general structure **3** can be designated as pleiotropic prodrugs, achieving a dual effect—one taking place during the metabolic activation, and the second one occurring after the activation—in contrast with the classic prodrugs, which by definition exhibit little to no pharmacological activity prior to the activation^37^, ^38^, ^39^. To the extent of our knowledge, this target combination has not yet been reported and validated *in vivo*.

Atipamezole **5** itself is a highly potent, low nanomolar, selective *α*_2_ adrenergic antagonist used in veterinary medicine to reverse the sedative and analgesic effects of (dex)medetomidine^40^, ^41^, ^42^. It is not *α*_2_ subtype selective, but is approx. 100-fold more potent and 200-fold more selective over the *α*_1_ adrenoreceptors compared to idazoxan **2**. Atipamezole exhibits negligible binding to other receptors (including monoaminergic and imidazoline receptors) or ion channels. In addition, it is rapidly absorbed and eliminated (half-life of 1.7–2 h in humans), has high volume of distribution and extensive first-pass metabolism. Unfortunately, it is not suitable for oral administration in humans^40^^,^^41^. It is also a pan-CYP450 reversible inhibitor and displays significant species difference in pharmacokinetics^43^^,^^44^. Especially in combination with ChEIs, subchronic administration of several *α*_2_ antagonists in different rodent models improved cognitive performance and diminished neuroinflammation^45^, ^46^, ^47^. For example, **5** decreased the number of high-voltage spindles, facilitated transmission of the dentate gyrus hippocampal cells, and improved the memory retention of rats in the radial arm maze task, performance of aged rats in linear arm maze and passive avoidance task, and memory consolidation. In active avoidance task, learning was improved with subchronic, but not acute administration regime. Low doses of **5** are likely to improve alertness, selective attention, planning, learning, recall, memory consolidation and plasticity, with only mild effects on emotional behaviour. Additionally, **5** increased sexual activity in rats and primates, improved recovery in brain damage models, and enhanced effects of anti-Parkinsonian drugs^41^^,^^48^, ^49^, ^50^, ^51^.

## 2 Results and discussion

### 2.1 Pleiotropic prodrug’s first target—increased steric demands lead to selective and prolonged BChE inhibition

First, the leaving groups themselves (compound **5** and its analogues **12**, **14**, **16**, Table 1) were tested for ChE inhibition, but proved ineffective. Therefore, the presence of carbamoyl warhead in compounds with general structure **3** was needed to achieve the first therapeutic effect, *i.e.*, inhibition of BChE. Covalent, pseudo-irreversible mechanism of action of *N*-carbamoylazoles (**6**–**11**, **13**, **15**, **17**) was confirmed by IC_50_ time-dependency and kinetic experiments (Supporting Information Figs. S1–S4, Table 2). Much to our surprise, *N*-carbamoylimidazoles **6**–**10** also achieved covalent inhibition of ChEs, in contrast with our previous findings.

**Table 1:** The biological activities of compounds **5**–**17** at ChEs and *α*_2A_ adrenoreceptors.

Structure	Compound	hBChE	hAChE	*α*_2A_^b^
		IC_50_±SD (nmol/L) or RA±SD (%)^a^ after 5 min preincubation		
		Time-dependency observed: Yes/No		
	**5**	Not active (82%±3%)	Not active (82%±3%)	100.6%^c^
	**6**	14±2 Yes	32,800±3900 Yes	6.0%
	**7**	9.9±0.9 Yes	52±3% Yes	d
	**8**	1520±90 Yes	Not active (62±4%) No	d
	**9**	2.5±2.0 Yes	7550±2140 Yes	d
	**10**	53±10 Yes	34%±12% Yes	d
	**11**	49%±2% Yes	Not active (69%±5%)	d
	**12**	Not active (67%±4%)	Not active (74%±2%)	14.8%
	**13**	8.4±4.6 Yes	3190±400 Yes	d
	**14**	Not active (82%±3%)	Not active (85%±2%)	6.1%
	**15**	1.9±0.5 Yes	234±46 Yes	d
	**16**	Not active (89%±6%)	Not active (83%±3%)	2.1%
	**17**	5.0±1.2 Yes	2930±1280 Yes	d

a SD: standard deviation of three independent experiments, each performed in triplicate. RA: residual activity at 100 μmol/L (mean±SD of three independent experiments performed in triplicate) is given in parentheses.

b Displacement assay at 1 μmol/L compound concentration with human *α*^2^A adrenoreceptor from CHO cells using antagonist radioligand [^3^*H*]RX821002. >50% inhibition is considered to represent significant binding of tested compounds. For details, see Supporting Information.

c *K*_i_(**5**) = 0.77 nmol/L—in agreement with the literature values: 1.2 nmol/L (human *α*_2A_ from CHO cells, [^3^*H*]RS79948-197 ligand)^52^ and 1.6 nmol/L (rat brain membranes, [^3^*H*]clonidine ligand)^41^.

d Not determined: *α*_2A_ binding was performed for representative compounds only.

**Table 2:** Kinetic parameters for hBChE inhibition^a^.

Compound	6	**7**	8
Reversible binding			
*K*_i_ [nmol/L]	116±3	73±6	2820±200
Carbamoylation			
*k*_carb_ [min^–1^]	9.0±2.5	7.2±4.5	3.7±1.7
${\Delta G}_{25\;{}^{\circ}C}^{‡}$ [kcal/mol]	18.6±0.2	18.7±0.4	19.1±0.3
Decarbamoylation			
*k*_decarb_ [×10^–2^ h^–1^]	3.59±0.28	0.92±0.14	∼0.22^53^
*t*_1/2_ [h]	19.3±1.5	75±11	∼318^53^

a Obtained by initial velocity measurements^53^ at 25 °C, values are given as mean±SD. Cf. Supporting Information Fig. S3 for further information. The reactivation rate of compound **8** is expected to be nearly identical to that of compound **11** from the previous study^53^, due to the presence of the same carbamoyl residue at Ser198.

In terms of structure–activity relationship, by varying the alkyl substituents on the carbamoyl moiety, the ChE selectivity profile could be fine-tuned—for example, from *N*,*N*-dimethylcarbamoyl **6** with 2500-fold selectivity for hBChE over hAChE to diethyl analogue **8** that bound selectively to hBChE. Furthermore, **8** had a decreased affinity and carbamoylation rate, while its decarbamoylation phase was prolonged >15-fold from days (as in case of **6**) to weeks (Table 2), which should allow *in vivo* BChE inhibition to build up with chronic administration. An inverse effect was observed upon increasing the electron-withdrawing character of the azole ring: going from imidazole **6**, 1,2,4-triazole **16**, 1,2,3-triazole **14**, to carbamoylimidazolium salt **13**, their inhibitory potencies increased with the concomitant loss of selectivity over hAChE. The increased electrophilicity and reactivity that leads to less specific catalytic Ser carbamoylation also roughly correlated with decreasing p*K*_a_ values of azolic leaving groups (and hydrolysis rates of *N*-carbamoylazoles in the buffer at physiological pH (Supporting Information Table S1).

**Figure 2:**
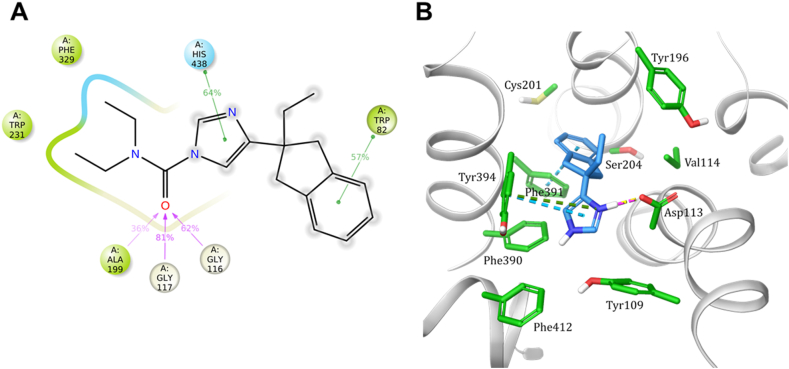
Analysis of molecular dynamics simulations. (A) Ligand interaction diagram based on 100 ns MD simulation of compound **8** in hBChE active site. Protein–ligand contacts and interactions that occur for more than 20% of the MD simulation time are shown: *π*–*π* interactions as green lines, cation–*π* interactions as red lines, hydrogen bonds are shown in blue and ionic interactions in magenta. Grey circles denote solvent exposure. (B) In lieu of crystal structure, the 500^th^ frame of the 1 μs MD simulation of compound **5** in *α*_2A_ adrenoreceptor (view from the top, orthogonally to the membrane). The key amino acid residues are shown as green sticks and **5** as azure sticks. Hydrogen bonds are shown as yellow dashed lines, *π*–*π* interactions as blue dashed lines, cation–*π* interactions as dark green dashed lines, and salt bridges as magenta dashed lines. This pose, locked in the receptor’s aromatic cage, prevailed during the last 500 ns.

The biological activity data were complemented by computational studies to provide an atomistic depiction of the dynamic processes occurring at both targets. Upon docking, *N*-carbamoylazoles produced highly comparable docking poses in the hBChE active site. For instance, the carbonyl oxygen of **8** was tridentately coordinated within the hBChE’s oxyanion hole comprised of Gly116, Gly117, and Ala199, ensuring optimal distance for the nucleophilic attack of catalytic Ser198 to occur. The *N*-alkyl substituents were oriented towards the acyl-binding pocket, the azole moiety formed a *π*–*π* interaction with His438, and the indan-2-yl moiety with Trp82 from choline-binding pocket (Fig. 2A). This binding mode was persistent during the first 85 ns of molecular dynamics (MD) simulations (Supporting Information Fig. S5). After carbamoylation, **8**–BChE intermediate disintegrates into Ser198–*N*,*N*-diethylcarbamoylated hBChE and leaving group **5**. Although the latter remained in the BChE active site during a long-timescale 2 μs of MD simulation, it formed only transient, dispersed interactions.

### 2.2 Pleiotropic prodrug’s second target—atipamezole is already optimal for α_2A_ adrenoreceptor

As was established from *in vitro* and *in silico* experiments above, the liberated leaving groups (compounds **5**, **12**, **14**, **16**) themselves only transiently bind to hBChE, implying that after carbamoylation this adrenoreceptor-active moiety can dissociate from the hBChE enzyme, and proceed to bind at *α*_2A_ receptors, thereby blocking the cascade leading from A*β* oligomers to ptau. However, all azolic leaving groups apart from atipamezole **5** did not possess any appreciable affinity for *α*_2A_ adrenoreceptors (Table 1, Fig. S4), therefore only *N*-carbamoylatipamezole derivatives **6**–**11** were considered for further studies.^41^, ^52^

Since the interaction of a cationic moiety of the ligand with Asp113^3.32^ residue is a necessary recognition motif in all monoaminergic receptors^25^, this could be the underlying reason for the lack of affinity for triazoles **14** and **16**. The latter are not ionized under physiological condition, as they are less basic than imidazole **5**—the p*K*_a_ values of the unsubstituted azoles are 1.2, 2.2, and 7.0, respectively^54^. In absence of a crystal structure, a 1 μs all-atom MD simulation of **5** in *α*_2A_ adrenoreceptor binding site confirmed a persistent hydrogen bond and an ionic interaction between protonated imidazole of **5** and Asp113^3.32^. The rest of the molecule engaged in recurrent van der Waals contacts as well as water bridges with lipophilic aromatic cage^25^ residues (Fig. 2B, Supporting Information Fig. S6). On the other hand, **14** and **16** featured different binding modes and/or quickly dissociated from the binding site *in silico*, which agrees with the lack of binding observed in the *α*_2A_ displacement assay (Fig. S6). Interestingly, **12** was inactive, as well, perhaps due to hindered rotation due to the additional *N*-methyl substituent (Fig. S6). It should be noted that atipamezole **5** analogues described in the literature always feature an unsubstituted imidazole ring^55^, ^56^, ^57^, ^58^, ^59^, ^60^, ^61^, ^62^.

### 2.3 Tuning the kinetics to engage both targets in the CNS

For these dual-acting compounds to achieve both desired therapeutic effects in the CNS—*i.e.*, the inhibition of BChE (symptomatic treatment and improvement of overall cognition) and *α*_2A_ antagonism (intervention by blocking disease mechanism), these pleiotropic prodrugs must permeate the blood–brain barrier and accumulate in the CNS at a sufficient level (Scheme 1). However, human plasma contains on average 4 mg hBChE/L^10^, thus upon peroral administration during absorption and distribution, significant metabolism of **3** could already take place in the plasma, before even reaching the desired site of action—the CNS. On the other hand, since metabolic activation concomitantly inactivates a stochiometric quantity of the BChE enzyme, a depletion of BChE in CNS could take place, preventing liberation of adrenergic antagonist **5** in the CNS, thus abolishing the second therapeutic benefit (arguably, this liberation could still take place in plasma, whereupon **5** would still permeate into CNS). In addition, due to its short half-life and fast elimination, to achieve a reasonable duration of action and application interval, **5** must be liberated in the CNS at a steady, slow enough rate to produce a lasting antagonistic effect at *α*_2A_ adrenoreceptors, while not depleting all BChE present in the tissues. Therefore, a fine tuning of the involved kinetic processes (*i.e.*, the rates of absorption, distribution into CNS, the plasma carbamoylation rate, metabolism of both *N*-carbamoylazole and **5**, rate of decarbamoylation) must be considered, and is most easily influenced by steric and electronic properties^53^^,^^63^ of the prodrug carbamoyl moiety (in red, Fig. 1 right). For these reasons, compound **8** was chosen to progress into further *in vivo* studies due to its selectivity for BChE over AChE, the markedly slow decarbamoylation phase, release of *α*_2A_-active moiety, and favourable physicochemical properties (Supporting Information Table S2).

**Figure 3:**
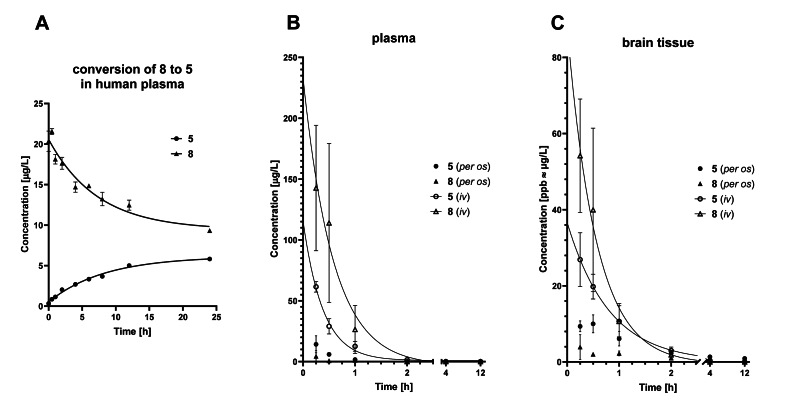
Preliminary pharmacokinetic analysis. (A) Hydrolysis of **8** (nominal starting concentration 20 μg/L) in human plasma at 37 °C, followed over the course of 24 h (*n* = 3, timepoints shown as means ± SD). The concentration of **8** is steadily decreasing (half-life 5.0 h), as it is hydrolysed by plasma hBChE to atipamezole **5**, the levels of which are rising. hBChE seems to be the main plasma hydrolase responsible for this pleiotropic prodrug activation since the pre-treatment of plasma with BChE-selective covalent inhibitor isoOMPA^64^ prevents this transformation (>85% **8** present after 24 h in a control experiment). (B, C) Concentration profiles of **5** and **8** in mice plasma (B) and brain (C) after peroral and intravenous administration of 1 mg/kg **8** to 3 months old BALB/c mice (*n* = 4 per group). Timepoints are shown as means ± SD and units are given in μg/L, which are roughly equivalent to ppb (*ρ*(mouse brain tissue) = 1.04 g/cm^3^)^65^.

In human plasma, **8** was slowly converted to **5** on a scale of several hours (Fig. 3A)^64^. With chronic administration of **8**, the total BChE inhibition is expected to build up gradually due to the long reactivation period of the carbamoylated enzyme (>10 days, Table 2), while providing a steady concentration of **5** present in the CNS. To test this hypothesis, compound **8** (1 or 5 mg/kg) was administered *p.o.* to C57BL/6JRccHsd mice once a day for 4 weeks. At the end of the treatment period, serum BChE activities dropped to 21%±6% (at 1 mg/kg dosage) or 10%±5% (at 5 mg/kg dosage) compared to activities before the start of treatment, while AChE activities were unchanged (Supporting Information Table S3). These results also alleviate the concerns of complete depletion of BChE in the body occurring upon chronic administration of compound **8**.

As seen from the plasma and brain distribution profile of compound **8** (1 mg/kg) after its intravenous and peroral administration to BALB/c mice (Fig. 3B and C)^65^, the prodrug **8** was absorbed from gastrointestinal tract and rapidly distributed across the body. Being highly lipophilic, **8** quickly permeated the blood–brain barrier and accumulated in the brain^65^, where it steadily liberated **5**. Due to the fast elimination rate of **5**, it could not be detected in plasma after 4 h, while its presence in the CNS endured for more than 12 h after *p.o.* administration. Ideally, this could translate into once or twice daily dosage regime—a significant improvement compared to atipamezole **5**.

**Figure 4:**
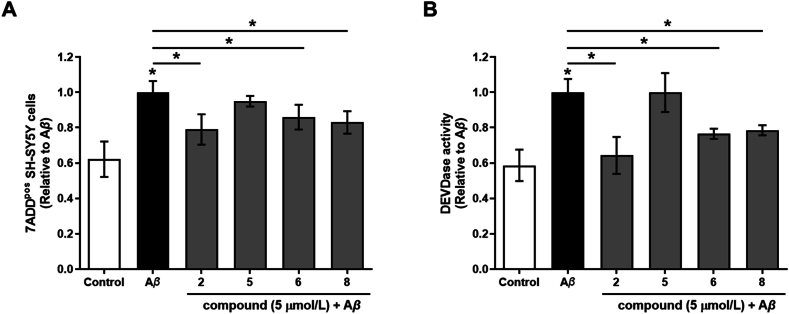
The effect of compounds on fibrillar A*β*_1–42_-induced toxicity in SH-SY5Y cells. SH-SY5Y cells were treated with pre-aggregated fibrillar A*β*_1–42_ (5 μmol/L), in the absence or presence of compounds (5 μmol/L). (A) After 48 h of treatment, neuroprotective effect was evaluated by flow cytometry analysis of 7AAD staining. The graph shows the results of quantitative analysis and indicates the percentage of dead cells, a fraction of 7AAD positive cells (7AAD^pos^). DMSO-treated cells were considered as control. Data are means ± SEM of three independent experiments, each performed in duplicate. (B) After 24 h treatment, caspase-3 activity in cell lysates was determined fluorometrically using the specific substrate for caspase 3, Ac-DEVD-AFC. DMSO-treated cells were considered as control. Data are presented as rates of fluorescence changes over time (Δ*F*/Δ*t*) with means ± SEM of three independent experiments, each performed in duplicate. ∗*P* ˂0.05.

Regarding safety, **8** had cytotoxic EC_50_ above 100 μmol/L in SH-SY5Y neuroblastoma cells (Supporting Information Figs. S7 and S8). Compounds **6** and **8** exerted a neuroprotective effect against A*β*_1-42_-induced toxicity in SH-SY5Y cells, as indicated by a significant reduction in 7-AAD-positive cells and caspase-3 activity (Fig. 4A and B, respectively), both of which were elevated following A*β*_1–42_ treatment. Furthermore, Western blot analysis revealed a consistent trend toward attenuation of tau phosphorylation at Thr181, suggesting an additional modulatory effect on tau signalling pathways (Supporting Information Fig. S9). These findings collectively support a multi-target neuroprotective action of the compounds on a cellular level. In a preliminary safety testing on mice with 1 mg/kg *p.o.* administered **8**, no effects were observed, whereas at 10 mg/kg **8** (10 × expected therapeutic dose), some adrenergic symptoms (marked vasodilation of tail and mucous veins, tachypnoea, tachycardia, increased activity, irritability) were observed 15–30 min after administration. This is consistent with *α*_2_ adrenoreceptor antagonism and CNS excitation mediated by liberated **5**^42^. After 4 to 5 h, all effects subsided. No acute cholinergic side effects, such as salivation or tremors that would be associated with peripheral AChE inhibition^66^, were observed. Needless to say, the liberated metabolite, atipamezole **5** is a registered drug with known safety profile.

### 2.4 Symptomatic cognitive improvement and disease-modifying effect demonstrated *in vivo*

**Figure 5:**
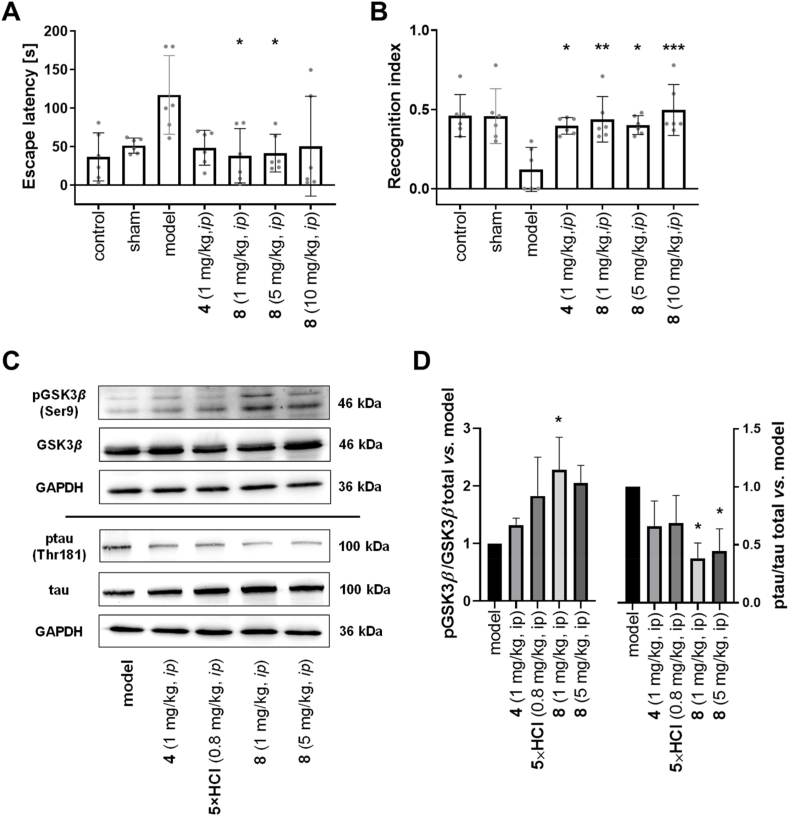
Behavioural assays and immunochemical analysis after *in vivo* application to mice. (A, B) The effects of compound **8** and rivastigmine **4** administration on cognitive impairment in an induced AD mice model. ICR mice were *icv*-injected saline (sham group) or A*β*_1–42_ peptide (10 μg), followed by either saline (model group), compound **4**, or compound **8** ip administration once a day from the third day onward in treatment groups. MWM task was conducted during days 9–14 and NOR test on Day 15. Values are expressed as means ± SD (*n* = 6; ∗∗∗*P* < 0.001, ∗∗*P* < 0.01, ∗*P* < 0.05 *vs.* model group). For more details, see Supporting Information. (A) The mice’ latency for the first escape to the platform in MWM test. (B) Recognition index in NOR test. For more information, see Figure S11. (C, D) The effects of compound **8** administration on tau hyperphosphorylation pathway in a transgenic APP/PS1 mice model. C57BL/6J-TgN(APP/PS1)ZLFILAS mice (6 months old) were treated with either saline (ip), **4** (1 mg/kg, ip), **5**×HCl (0.8 mg/kg, ip, this is *equimolar* to 1 mg/kg compound **8**), or compound **8** (1 or 5 mg/kg, ip) for 15 days, afterwards the animals were euthanized and their brain tissues were analysed. (C) Representative Western blots for determination of phosphorylated and total GSK3*β*, and phosphorylated and total tau levels in APP/PS1 mice brain homogenates obtained from different experimental groups. For raw images, see Supporting Information. (D) Ratios of phosphorylated and total form of either GSK3*β* or tau protein in APP/PS1 mice brain homogenates obtained from different experimental groups. The experiments were performed in triplicate and the ratios normalized *vs*. model group. Values are expressed as means ± SD (∗*P* < 0.05 *vs.* model).

The alleviation of cognitive impairment was evaluated *in vivo* in an induced AD murine model, where A*β*_1–42_ oligomers were intracerebroventricularly (icv) injected into the mice brain to induce AD-like pathological changes^67^, followed by multi-day intraperitoneal (ip) administration of tested compounds (Fig. 5A and B, Supporting Information Figs. S10 and S11). In Morris water maze (MWM) experiment, which evaluates effects on spatial working memory, short-term memory, and memory retrieval,^68^ the mice treated with compound **8** (1–5 mg/kg, ip) outperformed saline-treated mice (model) and were comparable to those that were treated with ChEI rivastigmine **4** (1 mg/kg, ip), which is currently the golden standard of AD therapy (Fig. 5A, Figs. S10 and S11). In novel object recognition task^69^ (NOR) compound **8** restored recognition memory comparable or better than **4** (Fig. 5B, Fig. S11), and it increased spontaneous alternation in the Y maze task, which suggests its positive impact on spatial working and reference memory^70^. Meanwhile, compound **7** was less effective in MWM test (Fig. S11).

Secondly, compound **8** was evaluated in a transgenic APP/PS1 mice model, and compared to its metabolite **5** (at equimolar dosage) and rivastigmine **4**. Although the high variability in the behavioural assays following a short treatment with the investigated compounds prevented demonstration of treatment effects on cognition (Supporting Information Fig. S12), the immunoassays on whole brain homogenates revealed that 15-day treatment with compound **8** increased phosphorylation of GSK3*β* (thus diminishing its activity) and also significantly lowered tau hyperphosphorylation (Fig. 5C and D). Of course, further studies of chronic, several months long treatment with compound **8** on transgenic AD model mice are needed to confirm the disease-modifying effects. However, this provides a preliminary mechanistic proof behind favourable *in vivo* effects of these multifunctional *α*_2A_-adrenoreceptor antagonists–butyrylcholinesterase inhibitors and demonstrates potential for their further development as both symptomatic and disease-modifying therapeutics for AD.

## 3 Conclusions

In summary, we report on a series of pleiotropic *N*-carbamoylazole prodrugs with a dual mechanism of action for the treatment of neurodegenerative diseases, such as AD. The first therapeutic target, hBChE, performs metabolic activation, becoming pseudo-irreversibly inactivated and causing symptomatic improvement of impaired cognitive functions until decarbamoylation is complete. Metabolic activation also liberates a low nanomolar selective *α*_2_ antagonist atipamezole, which binds to the second therapeutic target in the CNS, the *α*_2A_ adrenoreceptor, thereby blocking hyperphosphorylation of tau, and achieving the disease-modifying effect. This target combination, which has not been reported yet, offers a unique advantage in terms of merging both the symptomatic and disease-modifying treatment of the underlying AD aetiology, alleviating both the neurochemical and behavioural pathological changes in AD. The prodrug **8** was demonstrated to be perorally available in mice, liberating atipamezole **5** in contact with BChE present in plasma and brain tissue, leading to a prolonged BChE inhibition and *α*_2A_ antagonism on the scale of several days and hours, respectively. Subchronic administration of **8** in an induced A*β* mice model demonstrated its beneficial effects on spatial working, reference, and short-term memory, and memory retrieval. Furthermore, the improvement of cognitive deficits would originate not only from elevated ACh levels, but also from the blockade of A*β*-induced tau hyperphosphorylation, as was demonstrated in an APP/PS1 transgenic mice model. This approach clearly surpasses the symptomatic-only treatment of ChEIs, as it directly blocks an essential pathological cascade in AD, achieving disease-modifying effects. Therefore, these multifunctional *α*_2A_-adrenoreceptor antagonists–butyrylcholinesterase inhibitors, such as lead compound **8**, introduce an innovative, small molecule, symptomatic as well as disease-modifying treatment option for AD.

## 4 Experimental

### 4.1 Chemistry

#### 4.1.1 Chemistry—general information

The reagents and solvents were used as received from commercial suppliers. Tetrahydrofuran (THF) was distilled from sodium/benzophenone and stored under Ar over 4 Å molecular sieves prior to use. After extraction, organic phases were dried over anhydrous sodium sulfate. Reactions were monitored using analytical thin-layer chromatography (TLC) on silica gel 60 F_254_ Al plates. Developed plates were inspected under UV light and, if necessary, visualized with ninhydrin, vanillin/sulfuric acid, Dragendorff’s or potassium permanganate stains. Melting points were determined with Büchi 535 Melting Point Apparatus (uncorrected). Nuclear magnetic resonance spectra were recorded on a Bruker Avance III 400 MHz spectrometer at 400 MHz for ^1^H, 101 MHz for ^13^C, and 376 MHz for ^19^F nucleus, respectively, using CDCl_3_ with TMS as the internal standard as solvent. Chemical shifts are reported in parts per million (ppm), TMS peak was calibrated to 0 ppm or, alternatively, the central peak of the residual solvent resonance was used as the internal standard*—i.e.*, 7.27 ppm for ^1^H and 77.16 ppm for ^13^C, respectively. ^19^F spectra were not calibrated. The multiplicities are reported as follows: s (singlet), d (doublet), t (triplet), q (quartet), m (multiplet), dd (doublet of doublets), ddd (doublet doublet of doublets), td (triplet of doublets), qd (quartet of doublets), and br (broad), number of equivalent nuclei (by integration), coupling constants (*J*) quoted in Hertz (Hz). Mass spectra were recorded on Thermo Scientific Q Exactive Plus LC–MS/MS spectrometer and IR spectra on Thermo Nicolet FT-IR spectrophotometer. Column chromatography was performed on silica gel (Silica gel 60, particle size: 0.035–0.070 mm, Merck). UPLC analyses were performed on Thermo Scientific Dionex UltiMate 3000 modular system (Thermo Fisher Scientific Inc.). The general method used a Waters Acquity UPLC® HSS C18 SB column (2.1 mm × 50 mm, 1.8 μm) thermostated at 40 °C, with: injection volume, 5 μL; sample, 0.1–0.2 mg/mL in MeOH; flow rate, 0.4 mL/min; detector *λ*, 220 and 254 nm; mobile phase A: 0.1% TFA (*v*/*v*) in MilliQ water (Merck Millipore); mobile phase B: MeCN. Gradient: 0–5 min, 20%–100% B; 5–6 min, 20% B. For aqueous stability study, injection volume 5 μL and detection at 220 nm were used. The purities of the tested compounds were established to be ≥95%, as determined by UPLC.

General procedure—RP-CC: Compounds were purified by reversed-phase column chromatography (RP-CC) (Isolera Biotage One Flash Chromatography system, Biotage® Sfär C18 Duo 100 Å 30 μm column, 30 g, *V* = 45 mL) using a gradient of 0.1% TFA in deionized water and MeCN as eluent (gradient 0%–100% MeCN in 6 column volumes (300 mL); 100% MeCN for 2 column volumes (100 mL)). After the RP-CC, fractions containing the product were combined and organic volatiles were evaporated *in vacuo*. The remaining aqueous solution was made alkaline (pH 8) with 1 mol/L NaOH_(aq)_ and extracted with DCM (2 × 30 mL). The combined organic phase was dried over anhydrous sodium sulfate, filtered, and volatile components evaporated *in vacuo* to afford pure product.

#### 4.1.2 Atipamezole—4-(2-ethylindan-2-yl)-1H-imidazole (**5**)

Indane-2-carboxylic acid (5000 mg, 30.8 mmol) was dissolved in DMF (20 mL), potassium carbonate (3.0 eq., 12.9 g) and bromoethane (2.0 eq., 4.6 mL) were added, and the reaction mixture was vigorously stirred at room temperature for 12 h. Water (150 mL) was added, and the mixture extracted with diethyl ether (2 × 30 mL). The organic phase was dried with sodium sulfate, filtered, and solvent removed *in vacuo* to afford crude ethyl indane-2-carboxylate **4b**^71^, which was sufficiently pure to be used in the next step. Yield: 5842 mg (30.7 mmol, 99.7%) of brownish oil. ^1^H NMR (400 MHz, CDCl_3_) *δ* = 1.30 (t, *J* = 7.1, 3H), 3.18–3.39 (m, 5H), 4.20 (q, *J* = 7.1, 2H), 7.15–7.20 (m, 2H), 7.20–7.25 (m, 2H); ^13^C NMR (101 MHz, CDCl_3_) *δ* = 14.4, 36.3, 43.7, 60.7, 124.4, 126.7, 141.7, 175.4; *ν*_max_ 3024 (w), 2979 (w), 2854 (w), 1729 (vs), 1460 (m), 1371 (m), 1161 (s), 1024 (m), 861 (w), 740 (m) cm^–1^.

Diisopropylamine (1.25 eq., 4.6 mL) in anhydrous THF (20 mL) under Ar was cooled to –80 °C, *n*-butyllithium (1.2 eq., 12.5 mL, 2.5 mol/L in hexanes) was added dropwise, then the mixture was stirred at –10 °C for 10 min, cooled back to –80 °C, and ethyl indane-2-carboxylate (4944 mg, 26.0 mmol) in THF (10 mL) was added dropwise^72^. After stirring at –80 °C for 1 h, iodoethane (1.5 eq., 3.2 mL) was added, and the reaction mixture was left to warm up to room temperature during 4 h. 2 mol/L hydrochloric acid (25 mL) was added, the solvent removed *in vacuo*, the residue extracted with diethyl ether (2 × 30 mL), organic phase separated, dried with sodium sulfate, and the solvent removed *in vacuo* to afford crude ethyl 2-ethylindane-2-carboxylate, which was sufficiently pure to be directly used in the next step. Yield: 4779 mg (21.9 mmol, 84%) of brownish oil. ^1^H NMR (400 MHz, CDCl_3_) *δ* = 0.89 (t, *J* = 7.4, 3H), 1.26 (t, *J* = 7.1, 3H), 1.78 (q, *J* = 7.4, 2H), 2.90 (d, *J* = 16.0, 2H), 3.46 (d, *J* = 16.0, 2H), 4.16 (q, *J* = 7.1, 2H), 7.11–7.19 (m, 4H); ^13^C NMR (101 MHz, CDCl_3_) *δ* = 9.9, 14.4, 31.7, 41.8, 54.8, 60.7, 124.6, 126.6, 141.6, 177.0; *ν*_max_ 2966 (m), 2935 (w), 1726 (vs), 1460 (m), 1219 (m), 1177 (m), 1110 (m), 1032 (m), 862 (w), 739 (s) cm^–1^; ESI-HRMS: *m*/*z* = 219.1372 (MH^+^); C_14_H_19_O_2_ requires: *m*/*z* = 219.1380 (MH^+^).

Ethyl 2-ethylindane-2-carboxylate (3298 mg, 15.1 mmol) was dissolved in EtOH/H_2_O (1:1, 50 mL + 50 mL), sodium hydroxide (5.0 eq., 3021 mg) was added, and the mixture stirred at 80 °C for 48 h. The ethanol was removed *in vacuo*, the residue diluted with water (50 mL), extracted with diethyl ether (2 × 20 mL, discarded), acidified with concentrated hydrochloric acid to pH 1, and extracted with diethyl ether (2 × 30 mL). The organic phase was dried with sodium sulfate, filtered, and solvent removed *in vacuo* to afford crude 2-ethylindane-2-carboxylic acid^73^. Yield: 2635 mg (13.9 mmol, 92%) of brownish solid. ^1^H NMR (400 MHz, CDCl_3_) *δ* = 0.95 (t, *J* = 7.4, 3H), 1.83 (q, *J* = 7.4, 2H), 2.93 (d, *J* = 16.1, 2H), 3.50 (d, *J* = 16.1, 2H), 7.14–7.20 (m, 4H); ^13^C NMR (101 MHz, CDCl_3_) *δ* = 10.0, 31.5, 41.7, 54.8, 124.6, 126.7, 141.4, 183.7; *ν*_max_ 3022 (w), 2939 (w), 2852 (w), 2733 (w), 2643 (w), 1695 (vs), 1412 (m), 1338 (m), 1267 (s), 1183 (m), 942 (m), 733 (s) cm^–1^.

2-Ethylindane-2-carboxylic acid (2635 mg, 13.9 mmol) was azeotropically dried with toluene (30 mL), dissolved in DCM (10 mL), DMF (100 μL) was added, then oxalyl chloride (2.5 eq., 3 mL) was added dropwise under Ar, and the reaction mixture stirred at room temperature for 2 h. The volatiles were removed *in vacuo*, the residue dissolved in DCM (20 mL), cooled in an icebath, *N*,*O*-dimethylhydroxylamine hydrochloride (2.0 eq., 2702 mg) and triethylamine (3.5 eq., 6.8 mL) were added, and stirred at room temperature for 12 h. 2 mol/L hydrochloric acid (40 mL) was added, extracted with DCM (30 mL), aqueous phase discarded, organic phase extracted with 1 mol/L sodium hydroxide (20 mL, discarded), dried with sodium sulfate, filtered, and solvent removed *in vacuo*. 2-Ethyl-*N*-methoxy-*N*-methylindan-2-carboxamide was isolated by column chromatography on silica (1. PE/EtOAc = 5:1; 2. PE/EtOAc = 2:1). Yield: 2205 mg (9.45 mmol, 68%) of beige oil. ^1^H NMR (400 MHz, CDCl_3_) *δ* = 0.86 (t, *J* = 7.5, 3H), 1.76 (q, *J* = 7.5, 2H), 2.99 (d, *J* = 16.0, 2H), 3.24 (s, 3H), 3.47 (d, *J* = 16.1, 2H), 3.73 (s, 3H), 7.12–7.21 (m, 4H); *ν*_max_ 2963 (m), 2938 (m), 2878 (w), 1647 (vs), 1459 (s), 1364 (s), 1297 (m), 1175 (m), 1009 (m), 746 (s) cm^–1^.

2-Ethyl-*N*-methoxy-*N*-methylindane-2-carboxamide (2075 mg, 8.89 mmol) was azeotropically dried with toluene (30 mL), dissolved under Ar in anhydrous THF (10 mL), and cooled in an icebath. LiAlH_4_ (0.5 eq., 1.9 mL, 2.4 mol/L in THF) was added dropwise with stirring, and the stirring continued in an icebath for 1 h. 10% citric acid was added (30 mL), the solvent removed *in vacuo*, and the residue extracted with diethyl ether (2 × 20 mL). The organic phase was dried with sodium sulfate, filtered, and solvent removed *in vacuo* to afford crude 2-ethylindan-2-carbaldehyde^74^. Yield: 1350 mg (7.75 mmol, 87%) of beige oil, which was immediately used further in the next step. ^1^H NMR (400 MHz, CDCl_3_) *δ* = 0.90 (t, *J* = 7.5, 3H), 1.82 (q, *J* = 7.5, 2H), 2.85 (d, *J* = 16.0, 2H), 3.33 (d, *J* = 15.9, 2H), 7.13–7.20 (m, 4H), 9.60 (s, 1H); ^13^C NMR (101 MHz, CDCl_3_) *δ* = 9.7, 28.3, 38.5, 58.8, 124.7, 126.9, 141.1, 204.4.

2-Ethylindane-2-carbaldehyde (1430 mg, 8.21 mmol) was dissolved in absolute EtOH (8 mL), TosMIC (1.0 eq., 1602 mg) and NaCN (0.1 eq., 40 mg) were added, and the mixture stirred at room temperature for 12 h. The solvent was removed *in vacuo*, methanolic ammonia (25 mL, 4 mol/L), was added, and stirred at 80 °C for 48 h in a pressure tube^62^^,^^75^. The solvent was removed *in vacuo*, the residue dissolved in diethyl ether (30 mL), filtered, 5 mL of 2 mol/L ethereal HCl were added, the suspension sonicated for 2 min, the supernatant discarded, and the precipitate washed with diethyl ether (15 mL). 2 mol/L sodium hydroxide (10 mL) was added, extracted with DCM (2 × 30 mL), organic phase dried with sodium sulfate, the solvent removed *in vacuo*, and atipamezole **5**^55^^,^^73^^,^^76^, ^77^, ^78^, ^79^, ^80^, ^81^ isolated by column chromatography on silica (1. DCM/MeOH = 50:1; 2. DCM/MeOH = 15:1). Yield: 818 mg (3.86 mmol, 47%) of beige solid. mp 123.2–124.4 °C (lit.^76^ 122–126 °C; ^1^H NMR (400 MHz, CDCl_3_) *δ* = 0.83 (t, *J* = 7.4, 3H), 1.92 (q, *J* = 7.3, 2H), 3.11 (d, *J* = 15.6, 2H), 3.34 (d, *J* = 15.6, 2H), 6.82 (d, *J* = 0.9, 1H), 7.13–7.17 (m, 2H), 7.18–7.22 (m, 2H), 7.59 (d, *J* = 0.9, 1H), 11.25 (s, 1H); ^13^C NMR (101 MHz, CDCl_3_) *δ* = 9.9, 33.2, 44.6, 47.7, 117.0, 124.6, 126.3, 134.9, 142.5, 143.4;); *ν*_max_ 3069 (s), 2967 (s), 2914 (s), 2851 (vs), 2642 (br), 1668 (w), 1572 (w), 1473 (s), 1458 (vs), 1303 (s), 939 (m), 833 (s), 739 (s), 629 (s) cm^–1^; ESI-HRMS: *m*/*z* = 213.1378 (MH^+^); C_14_H_17_N_2_ requires: *m*/*z* = 213.1386 (MH^+^); Purity: UPLC (254 nm): *t*_r_ = 1.740 min, 99.0% total area.

#### 4.1.3 4-(2-Ethylindan-2-yl)-N,N-dimethyl-1H-imidazole-1-carboxamide (**6**)

Atipamezole **5** (104 mg, 0.49 mmol), DIPEA (5.0 eq., 427 μL) and *N*,*N*-dimethylcarbamoyl chloride (5.0 eq., 226 μL) in MeCN (5 mL) were stirred under Ar at 60 °C for 6 h. The solvent was removed *in vacuo* and 4-(2-ethylindan-2-yl)-*N*,*N*-dimethyl-1*H*-imidazole-1-carboxamide **6** isolated by column chromatography on silica (1. PE/EtOAc = 1:1; 2. EtOAc). Yield: 117 mg (0.41 mmol, 84%) of off-white solid. mp 121.1–122.2 °C; ^1^H NMR (400 MHz, CDCl_3_) *δ* = 0.74 (t, *J* = 7.4, 3H), 1.89 (q, *J* = 7.4, 2H), 3.00 (s, 6H), 3.03 (d, *J* = 15.8, 2H), 3.24 (d, *J* = 15.6, 2H), 6.89 (d, *J* = 1.2, 1H), 7.05–7.09 (m, 2H), 7.12–7.15 (m, 2H), 7.80 (d, *J* = 1.2, 1H); ^13^C NMR (101 MHz, CDCl_3_) *δ* = 9.8, 32.6, 38.3, 44.1, 48.4, 113.9, 124.4, 126.1, 136.5, 142.5, 148.8, 151.9; *ν*_max_ 3111 (w), 2976 (w), 2959 (w), 2929 (w), 1672 (vs), 1478 (m), 1401 (s), 1257 (m), 1243 (m), 1188 (s), 1011 (s), 742 (s) cm^–1^; ESI-HRMS: *m*/*z* = 284.1746 (MH^+^); C_17_H_22_N_3_O requires: *m*/*z* = 284.1758 (MH^+^); Purity: UPLC (254 nm): *t*_r_ = 2.073 min, 97.6%.

#### 4.1.4 N-Ethyl-4-(2-ethylindan-2-yl)-N-methyl-1H-imidazole-1-carboxamide (**7**)

Atipamezole **5** (539 mg, 2.54 mmol), sodium hydride (2.0 eq., 203 mg, 60 wt.% dispersion in mineral oil) and ethylmethylcarbamoyl chloride (2.0 eq., 612 μL) in THF (5 mL) were stirred under Ar at rt for 2 h. The solvent was removed *in vacuo* and *N*-ethyl-4-(2-ethylindan-2-yl)-*N*-methyl-1*H*-imidazole-1-carboxamide **7** isolated by column chromatography on silica (1. PE/EtOAc = 3:1; 2. PE/EtOAc = 1:1). Yield: 603 mg (2.03 mmol, 80%) of beige semisolid. ^1^H NMR (400 MHz, CDCl_3_) *δ* = 0.76 (t, *J* = 7.4, 3H), 1.20 (t, *J* = 7.2, 3H), 1.91 (q, *J* = 7.4, 2H), 2.98 (s, 3H), 3.05 (d, *J* = 15.6, 2H), 3.25 (d, *J* = 15.6, 2H), 3.38 (q, *J* = 7.1, 2H), 6.89 (d, *J* = 1.3, 1H), 7.07–7.12 (m, 2H), 7.14–7.19 (m, 2H), 7.80 (d, *J* = 1.3, 1H); ^13^C NMR (101 MHz, CDCl_3_) *δ* = 10.0, 12.5, 32.7, 35.6, 44.2, 45.3, 48.5, 114.0, 124.5, 126.2, 136.4, 142.6, 148.9, 151.7; *ν*_max_ 3116 (w), 2966 (w), 2876 (w), 1674 (vs), 1441 (m), 1401 (s), 1283 (m), 1185 (m), 1022 (m), 860 (w), 742 (m) cm^–1^; ESI-HRMS: *m*/*z* = 298.1910 (MH^+^); C_18_H_24_N_3_O requires: *m*/*z* = 298.1914 (MH^+^); Purity: UPLC (220 nm): *t*_r_ = 2.307 min, 98.5% total area.

#### 4.1.5 N,N-Diethyl-4-(2-ethylindan-2-yl)-1H-imidazole-1-carboxamide (**8**)

Atipamezole **5** (39 mg, 0.184 mmol), DIPEA (5.0 eq., 160 μL), and *N*,*N*-diethylcarbamoyl chloride (5.0 eq., 114 μL) in MeCN (3 mL) were stirred under Ar at 60 °C for 6 h. The solvent was removed *in vacuo* and *N*,*N*-diethyl-4-(2-ethylindan-2-yl)-1*H*-imidazole-1-carboxamide **8** isolated by column chromatography on silica (1. PE/EtOAc = 2:1; 2. PE/EtOAc = 1:1). Yield: 52 mg (0.167 mmol, 91%) of beige solid. mp 60.0–60.8 °C; ^1^H NMR (400 MHz, CDCl_3_) *δ* = 0.77 (t, *J* = 7.4, 3H), 1.18 (t, *J* = 7.1, 6H), 1.92 (q, *J* = 7.4, 2H), 3.05 (d, *J* = 15.6, 2H), 3.24 (d, *J* = 15.6, 2H), 3.34 (q, *J* = 7.1, 4H), 6.87 (d, *J* = 1.3, 1H), 7.07–7.12 (m, 2H), 7.14–7.18 (m, 2H), 7.79 (d, *J* = 1.3, 1H); ^13^C NMR (101 MHz, CDCl_3_) *δ* = 10.0, 13.2, 32.7, 42.6, 44.3, 48.6, 113.9, 124.5, 126.2, 136.2, 142.7, 148.9, 151.4; *ν*_max_ 3129 (w), 3107 (w), 2959 (m), 2929 (m), 1680 (vs), 1425 (s), 1360 (m), 1267 (s), 1218 (m), 1162 (m), 1027 (m), 952 (w), 855 (w), 752 (m), 744 (m), 674 (w), 629 (w) cm^–1^; ESI-HRMS: *m*/*z* = 312.2056 (MH^+^); C_19_H_26_N_3_O requires: *m*/*z* = 312.2070 (MH^+^); Purity: UPLC (220 nm): *t*_r_ = 2.507 min, 99.7% total area.

#### 4.1.6 N,N-Diethyl-4-(2-(ethyl-d_5_)indan-2-yl)-1H-imidazole-1-carboxamide (**8**-d_5_)

Diisopropylamine (1.2 eq., 3.75 mL) in anhydrous THF (20 mL) under Ar was cooled to –80 °C, *n*-butyllithium (1.15 eq., 10.3 mL, 2.5 mol/L in hexanes) was added dropwise, then the mixture was stirred at 0 °C for 10 min, cooled back to –80 °C, and ethyl indane-2-carboxylate (4244 mg, 22.3 mmol) in THF (10 mL) was added dropwise. After stirring at –80 °C for 1 h, bromoethane-*d*_5_ (1.2 eq., 2 mL, 99 atom % D) was added, and the reaction mixture was stirred at –80 °C for 2 h, and then left to warm up to room temperature during 4 h. 2 mol/L Hydrochloric acid (25 mL) was added, the solvent removed *in vacuo*, the residue extracted with diethyl ether (2 × 30 mL), organic phase separated, dried with sodium sulfate, and the solvent removed *in vacuo* to afford crude ethyl 2-(ethyl-*d*_5_)indane-2-carboxylate **5**-*d*_5_**a** (4660 mg) as yellowish oil, which was immediately used in the next steps as described for **5** to afford 2-(ethyl-*d*_5_)indan-2-carboxylic acid **5**-*d*_5_**b** (3499 mg), 2-(ethyl-*d*_5_)-*N*-methoxy-*N*-methylindan-2-carboxamide **5**-*d*_5_**c** (3035 mg), 2-(ethyl-*d*_5_)indan-2-carbaldehyde **5**-*d*_5_**d** (2875 mg), and 4-(2-(ethyl-*d*_5_)indan-2-yl)-1*H*-imidazole/atipamezole-*d*_5_ **5**-*d*_5_ (346 mg). **5**-*d*_5_: ^1^H NMR (400 MHz, CDCl_3_) *δ* = 3.11 (d, *J* = 15.6, 2H), 3.34 (d, *J* = 15.6, 2H), 6.81 (d, *J* = 1.0, 1H), 7.13–7.18 (m, 2H), 7.19–7.23 (m, 2H), 7.58 (d, *J* = 1.0, 1H), 11.86 (br s, 1H); ^13^C NMR (101 MHz, CDCl_3_) *δ* = 8.9 (hept, *J* = 19.1), 31.7–32.6 (m), 44.6, 47.5, 116.9 (br s), 124.6, 126.3, 134.9, 142.6, 143.5 (br s); *ν*_max_ 3065 (s), 2913 (s), 2847 (s), 2732 (w), 2646 (s), 2225 (m), 1572 (w), 1483 (s), 1474 (s), 1456 (s), 1436 (s), 1309 (s), 1224 (m), 1017 (m), 957 (m), 834 (s), 739 (s), 630 (s) cm^–1^; ESI-HRMS: *m*/*z* = 218.1700 (MH^+^); C_14_H_12_D_5_N_2_ requires: *m*/*z* = 218.1700 (MH^+^); Purity: UPLC (220 nm): *t*_r_ = 2.573 min, 99.1%.

Atipamezole-*d*_5_ **5**-*d*_5_ (106 mg, 0.488 mmol), NaH (1.5 eq., 29 mg, 60 wt.% dispersion in mineral oil), and *N*,*N*-diethylcarbamoyl chloride (1.5 eq., 93 μL) in MeCN (3 mL) were stirred under Ar at room temperature for 4 h. The solvent was removed *in vacuo* and *N*,*N*-diethyl-4-(2-(ethyl-*d*_5_)indan-2-yl)-1*H*-imidazole-1-carboxamide **8**-*d*_5_ isolated by column chromatography on silica (1. PE/EtOAc = 2:1; 2. PE/EtOAc = 1:1). Yield: 40 mg (0.126 mmol, 26%) of white solid. ^1^H NMR (400 MHz, CDCl_3_) *δ* = 1.20 (t, *J* = 7.1, 6H), 3.05 (d, *J* = 15.6, 2H), 3.25 (d, *J* = 15.6, 2H), 3.36 (q, *J* = 7.1, 4H), 6.87 (d, *J* = 1.3, 1H), 7.09–7.13 (m, 2H), 7.15–7.19 (m, 2H), 7.80 (d, *J* = 1.3, 1H); ^13^C NMR (101 MHz, CDCl_3_) *δ* = 13.3, 42.7, 44.3, 48.5, 113.9, 124.6, 126.3, 136.2, 142.8, 149.1, 151.6; *ν*_max_ 3130 (w), 3107 (w), 2972 (w), 2936 (w), 2896 (w), 2216 (m), 1679 (vs), 1472 (m), 1422 (m), 1359 (m), 1267 (m), 1217 (m), 1160 (m), 953 (m), 856 (m), 752 (m) cm^–1^; ESI-HRMS: *m*/*z* = 317.2379 (MH^+^); C_19_H_21_D_5_N_3_O requires: *m*/*z* = 317.2384 (MH^+^); Purity: UPLC (220 nm): *t*_r_ = 1.867 min, 98.2%.

#### 4.1.7 (4-(2-Ethylindan-2-yl)-1H-imidazol-1-yl)(piperidin-1-yl)methanone (**9**)

Atipamezole **5** (49 mg, 0.231 mmol), DIPEA (5.0 eq., 201 μL), and 3-methyl-1-(piperidine-1-carbonyl)-1*H*-imidazol-3-ium iodide (prepared *in situ* from (1*H*-imidazol-1-yl)(piperidin-1-yl)methanone and methyl iodide in MeCN; 1.04 mmol, 4.5 eq.) in MeCN (5 mL) were stirred under Ar at 60 °C for 4 h. The solvent was removed *in vacuo* and (4-(2-ethylindan-2-yl)-1*H*-imidazol-1-yl)(piperidin-1-yl)methanone **9** isolated by column chromatography on silica (1. PE/EtOAc = 2:1; 2. PE/EtOAc = 1:1). Yield: 29 mg (0.0897 mmol, 39%) of beige semisolid. ^1^H NMR (400 MHz, CDCl_3_) *δ* = 0.77 (t, *J* = 7.4, 3H), 1.57–1.64 (m, 4H), 1.64–1.71 (m, 2H), 1.91 (q, *J* = 7.4, 2H), 3.05 (d, *J* = 15.6, 2H), 3.25 (d, *J* = 15.6, 2H), 3.43–3.48 (m, 4H), 6.84 (d, *J* = 1.3, 1H), 7.08–7.12 (m, 2H), 7.14–7.19 (m, 2H), 7.77 (d, *J* = 1.3, 1H); ^13^C NMR (101 MHz, CDCl_3_) *δ* = 10.0, 24.3, 25.9, 32.7, 44.3, 47.6, 48.6, 114.0, 124.6, 126.3, 136.5, 142.7, 149.0, 151.2; *ν*_max_ 3114 (w), 2943 (w), 2902 (w), 2847 (w), 1683 (vs), 1428 (m), 1259 (m), 1163 (m), 742 (m) cm^–1^; ESI-HRMS: *m*/*z* = 324.2065 (MH^+^); C_20_H_26_N_3_O requires: *m*/*z* = 324.2070 (MH^+^); Purity: UPLC (220 nm): *t*_r_ = 2.653 min, 98.1% total area.

#### 4.1.8 N-Benzyl-4-(2-ethylindan-2-yl)-N-methyl-1H-imidazole-1-carboxamide (**10**)

Atipamezole **5** (68 mg, 0.32 mmol), NaH (1.5 eq., 19 mg, 60 wt.% dispersion in mineral oil) and benzyl(methyl)carbamic fluoride (2.0 eq., 107 mg) in MeCN (3 mL) were stirred under Ar at 60 °C for 6 h. The solvent was removed *in vacuo* and *N*-benzyl-4-(2-ethylindan-2-yl)-*N*-methyl-1*H*-imidazole-1-carboxamide **10** isolated by column chromatography on silica (1. PE/EtOAc = 3:1; 2. PE/EtOAc = 1:1). Yield: 28 mg (0.078 mmol, 24%) of beige semisolid. ^1^H NMR (400 MHz, CDCl_3_) *δ* = 0.76 (t, *J* = 7.4, 3H), 1.91 (q, *J* = 7.4, 2H), 2.98 (s, 3H), 3.03 (d, *J* = 15.6, 2H), 3.22 (d, *J* = 15.6, 2H), 4.57 (s, 2H), 6.93 (d, *J* = 1.3, 1H), 7.07–7.16 (m, 4H), 7.22–7.26 (m, 2H), 7.31–7.41 (m, 3H), 7.85 (d, *J* = 1.3, 1H). ^13^C NMR (101 MHz, CDCl_3_) *δ* = 10.0, 32.7, 36.5, 44.2, 48.6, 54.2, 114.1, 124.5, 126.3, 127.5, 128.2, 129.1, 135.6, 136.6, 142.6, 149.1, 152.3; *ν*_max_ 3119 (w), 2957 (m), 2930 (w), 2853 (w), 1673 (vs), 1470 (m), 1457 (m), 1425 (m), 1405 (m), 1257 (m), 1141 (w), 1080 (w), 1020 (w). 951 (w), 741 (m), 738 (m) cm^–1^; ESI-HRMS: *m*/*z* = 360.2063 (MH^+^); C_23_H_26_N_3_O requires: *m*/*z* = 360.2070 (MH^+^); Purity: UPLC (220 nm): *t*_r_ = 3.000 min, 97.0% total area.

#### 4.1.9 4-(2-Ethylindan-2-yl)-N,N-dimethyl-1H-imidazole-1-carbothioamide (**11**)

Atipamezole **5** (30 mg, 0.141 mmol), NaH (2.0 eq., 11 mg, 60 wt.% dispersion in mineral oil), and dimethylthiocarbamoyl chloride (2.0 eq., 35 mg) in MeCN (2 mL) were stirred under Ar at 60 °C for 4 h. The solvent was removed *in vacuo* and 4-(2-ethylindan-2-yl)-*N*,*N*-dimethyl-1*H*-imidazole-1-carbothioamide **11** isolated by column chromatography on silica (1. PE/EtOAc = 3:1; 2. PE/EtOAc = 1:1). Yield: 19 mg (0.0635 mmol, 45%) of yellowish semisolid. ^1^H NMR (400 MHz, CDCl_3_) *δ* = 0.78 (t, *J* = 7.4, 3H), 1.90 (q, *J* = 7.4, 2H), 3.05 (d, *J* = 15.6, 2H), 3.16–3.39 (m, 8H), 6.88 (d, *J* = 1.3, 1H), 7.08–7.12 (m, 2H), 7.15–7.19 (m, 2H), 7.83 (d, *J* = 1.3, 1H); ^13^C NMR (101 MHz, CDCl_3_) *δ* = 10.0, 32.7, 43.8, 44.2, 48.6, 115.4, 124.6, 126.3, 137.2, 142.6, 149.3, 179.8; *ν*_max_ 3116 (m), 2956 (m), 2944 (m), 2841 (m), 1539 (s), 1483 (m), 1397 (m), 1369 (m), 1252 (m), 1139 (m), 1046 (m), 952 (m), 865 (w), 742 (vs) cm^–1^; ESI-HRMS: *m*/*z* = 300.1518 (MH^+^); C_17_H_22_N_3_S requires: *m*/*z* = 300.1529 (MH^+^); Purity: UPLC (254 nm): *t*_r_ = 2.560 min, 96.1% total area.

#### 4.1.10 5-(2-Ethylindan-2-yl)-1-methyl-1H-imidazole (**12**)

**13** (97 mg, 0.217 mmol) was dissolved in MeOH, 50% NaOH (100 μL) was added, and the mixture stirred for 4 h at room temperature. The solvent was removed *in vacuo* and 5-(2-ethylindan-2-yl)-1-methyl-1*H*-imidazole **12** isolated by column chromatography on silica (1. DCM/MeOH = 20:1; 2. DCM/MeOH = 5:1). Yield: 37 mg (0.164 mmol, 75%) of colourless oil. ^1^H NMR (400 MHz, CDCl_3_) *δ* = 0.68 (t, *J* = 7.4, 3H), 1.73 (q, *J* = 7.4, 2H), 3.15 (d, *J* = 15.3, 2H), 3.31 (d, *J* = 15.3, 2H), 3.69 (s, 3H), 6.81 (s, 1H), 7.12–7.17 (m, 2H), 7.18–7.23 (m, 2H), 7.35 (s, 1H); ^13^C NMR (101 MHz, CDCl_3_) *δ* = 9.4, 31.1, 33.4, 44.0, 46.4, 124.6, 126.6, 127.9, 136.9, 139.5, 141.6; *ν*_max_ 3388 (br), 2962 (vs), 2932 (s), 2874 (m), 1637 (br), 1500 (vs), 1485 (s), 1458 (vs), 1378 (w), 1237 (s), 1120 (s), 912 (m), 816 (m), 743 (vs), 690 (m), 657 (s) cm^–1^; ESI-HRMS: *m*/*z* = 227.1531 (MH^+^); C_15_H_19_N_2_ requires: *m*/*z* = 227.1543 (MH^+^); Purity: UPLC (254 nm): *t*_r_ = 1.943 min, 97.2% total area.

#### 4.1.11 1-(Dimethylcarbamoyl)-4-(2-ethylindan-2-yl)-3-methyl-1H-imidazol-3-ium trifluoromethanesulfonate (**13**)

4-(2-Ethylindan-2-yl)-*N*,*N*-dimethyl-1*H*-imidazole-1-carboxamide **6** (83 mg, 0.293 mmol) was azeotropically dried with toluene (10 mL), dissolved under Ar in anhydrous DCM (4 mL), and cooled in an icebath. Methyl triflate (1.5 eq., 50 μL) was added and the mixture stirred at room temperature for 3 h. Diethyl ether (30 mL) was added, the mixture sonicated for 2 min, the supernatant decanted, the residue washed with diethyl ether (2 × 10 mL), and dried *in vacuo*. Yield: 116 mg (0.258 mmol, 89%) of beige semisolid. ^1^H NMR (400 MHz, CDCl_3_) *δ* = 0.73 (t, *J* = 7.3, 3H), 1.82 (q, *J* = 7.2, 2H), 3.09 (s, 6H), 3.21 (d, *J* = 15.6, 2H), 3.33 (d, *J* = 15.6, 2H), 4.03 (s, 3H), 7.13–7.22 (m, 4H), 7.45 (d, *J* = 1.3, 1H), 9.32 (s, 1H); ^13^C NMR (101 MHz, CDCl_3_) *δ* = 9.2, 30.5, 36.7, 38.7, 43.4, 46.5, 118.7, 119.1 (q, *J* = 319.7, 320.7), 124.7, 127.2, 138.8, 140.0, 140.6, 147.4; ^19^F NMR (376 MHz, CDCl_3_) *δ* = –78.49; *ν*_max_ 3587 (br), 2963 (m), 2855 (w), 1735 (vs), 1486 (w), 1460 (w), 1390 (m), 1258 (vs), 1161 (s), 1031 (s), 638 (m) cm^–1^; ESI-HRMS: *m*/*z* = 298.1901 (M^+^); C_18_H_24_N_3_O requires: *m*/*z* = 298.1914 (M^+^); Purity: UPLC (220 nm): *t*_r_ = 2.147 min, 95.1% total area.

#### 4.1.12 4-(2-Ethylindan-2-yl)-2H-1,2,3-triazole (**14**)

2-Ethylindane-2-carboxylic acid (3100 mg, 16.3 mmol) was azeotropically dried with toluene (50 mL), dissolved in DCM (10 mL), DMF (100 μL) was added. Oxalyl chloride (3.0 eq., 4.2 mL) was added dropwise with stirring under Ar at room temperature. After 2 h, the volatiles were removed *in vacuo*, the residue redissolved in DCM (20 mL), ammonium bicarbonate (6.0 eq., 7.7 g) was added (gas effervescence) and the mixture vigorously stirred at room temperature for 18 h. Water (50 mL) was then added, and 2-ethylindane-2-carboxamide extracted into DCM (2 × 30 mL). The organic phase was dried with sodium sulfate, filtered, and the solvent removed *in vacuo*.

The crude 2-ethylindane-2-carboxamide (2015 mg, 10.65 mmol) was azeotropically dried with toluene (50 mL), dissolved in DCM (15 mL), triethylamine was added (4.0 eq., 6.7 mL), the mixture cooled in an icebath under Ar, and trifluoroacetic anhydride (2.0 eq., 2.96 mL) was added dropwise. After stirring for 2 h at room temperature, water (50 mL) and DCM (10 mL) were added, the organic phase successively extracted with 2 mol/L hydrochloric acid (15 mL) and 1 mol/L sodium hydroxide (20 mL), the solvent was removed *in vacuo*, and 2-ethylindane-2-carbonitrile isolated by column chromatography on silica (1. PE; 2. PE/DCM = 1:1). Yield: 1220 mg (7.13 mmol, 44% over three steps) of yellowish oil. ^1^H NMR (400 MHz, CDCl_3_) *δ* = 1.17 (t, *J* = 7.4, 3H), 1.80 (q, *J* = 7.4, 2H), 3.06 (d, *J* = 15.7, 2H), 3.44 (d, *J* = 15.7, 2H), 7.20–7.24 (m, 4H); ^13^C NMR (101 MHz, CDCl_3_) *δ* = 10.3, 31.2, 43.7, 43.8, 124.7, 124.8, 127.3, 139.6; *ν*_max_ 3269 (m), 2971 (m), 2845 (w), 2234 (w), 1722 (vs), 1639 (vs), 1496 (vs), 1465 (s), 1441 (s), 1237 (vs), 1155 (vs), 1024 (s), 762 (s) cm^–1^.

Trimethylsilyldiazomethane (1.6 eq., 5.43 mL, 2 mol/L in hexanes) in anhydrous THF (5 mL) was cooled to –10 °C in a methanol-ice bath, *n*-butyllithium (1.6 eq., 4.3 mL, 2.5 mol/L in hexanes) was added dropwise, and the mixture stirred for 30 min at this temperature^82^. 2-Ethylindane-2-carbonitrile (1162 mg, 6.79 mmol) in THF (3 mL) was then added and the reaction mixture left to warm up to room temperature within 4 h. Methanol (20 mL), potassium bifluoride (500 mg), and water (10 mL) were added, and the mixture stirred vigorously for 24 h. The solvent was removed *in vacuo*, the product extracted into diethyl ether (2 × 20 mL), and 4-(2-ethylindan-2-yl)-2*H*-1,2,3-triazole **14** isolated by column chromatography on silica (1. PE/EtOAc = 5:1; 2. PE/EtOAc = 2:1). Yield: 1191 mg (5.59 mmol, 82%) of yellowish semisolid. ^1^H NMR (400 MHz, CDCl_3_) *δ* = 0.80 (t, *J* = 7.4, 3H), 1.94 (q, *J* = 7.4, 2H), 3.18 (d, *J* = 15.6, 2H), 3.36 (d, *J* = 15.6, 2H), 7.12–7.17 (m, 2H), 7.18–7.23 (m, 2H), 7.52 (s, 1H), 13.30 (s, 1H); ^13^C NMR (101 MHz, CDCl_3_) *δ* = 9.9, 33.5, 44.8, 47.1, 124.7, 126.6, 130.8, 142.1, 153.7; *ν*_max_ 3130 (s), 2962 (vs), 2935 (vs), 2844 (s), 1483 (m), 1458 (m), 744 (m) cm^–1^; ESI-HRMS: *m*/*z* = 214.1329 (MH^+^); C_13_H_16_N_3_ requires: *m*/*z* = 214.1339 (MH^+^); Purity: UPLC (220 nm): *t*_r_ = 2.667 min, 98.0% total area.

#### 4.1.13 4-(2-Ethylindan-2-yl)-N,N-dimethyl-2H-1,2,3-triazole-2-carboxamide (**15**)

4-(2-Ethylindan-2-yl)-2*H*-1,2,3-triazole **14** (689 mg, 3.23 mmol), *N*,*N*-dimethylcarbamoyl chloride (5 eq., 1487 μL), and DIPEA (6 eq., 3.38 mL) in MeCN (10 mL) were stirred at 80 °C for 2 h. The solvent was removed *in vacuo* and 4-(2-ethylindan-2-yl)-*N*,*N*-dimethyl-2*H*-1,2,3-triazole-2-carboxamide **15** was isolated by RP-CC as the first of two barely resolved peaks. Yield: 27 mg (0.0949 mmol, 2.9%) of beige oil. ^1^H NMR (400 MHz, CDCl_3_) *δ* = 0.79 (t, *J* = 7.4, 3H), 2.00 (q, *J* = 7.4, 2H), 3.12–3.19 (m, 5H), 3.31–3.39 (m, 5H), 7.11–7.15 (m, 2H), 7.16–7.20 (m, 2H), 7.85 (s, 1H); ^13^C NMR (101 MHz, CDCl_3_) *δ* = 9.9, 33.1, 38.6, 40.0, 44.7, 47.3, 122.0, 124.7, 126.6, 142.0, 149.6, 153.4; *ν*_max_ 3143 (w), 3022 (w), 2962 (m), 2934 (m), 2850 (w), 1704 (vs), 1482 (s), 1458 (s), 1405 (s), 1279 (m), 1254 (m), 1227 (m), 1155 (s), 1028 (s), 998 (s), 743 (s) cm^–1^; ESI-HRMS: *m*/*z* = 285.1697 (MH^+^); C_16_H_21_N_4_O requires: *m*/*z* = 285.1710 (MH^+^); Purity: UPLC (220 nm): *t*_r_ = 3.207 min, 95.8% total area.

#### 4.1.14 3-(2-Ethylindan-2-yl)-1H-1,2,4-triazole (**16**)

2-Ethylindan-2-carboxylic acid (648 mg, 3.4 mmol) was converted to carboxamide, as described under **10**. The crude 2-ethylindane-2-carboxamide and dimethylformamide dimethylacetal (DMFDMA, 10.0 eq., 4.53 mL) were stirred under Ar at 100 °C for 4 h. The volatiles were removed *in vacuo*, the residue was dissolved in glacial acetic acid (3 mL), hydrazine monohydrate (1.1 eq., 184 μL) was added, and the reaction mixture stirred at 60 °C for 12 h^83^. 3-(2-Ethylindan-2-yl)-1*H*-1,2,4-triazole **16** was isolated following RP-CC. Yield: 191 mg (0.896 mmol, 26% over four steps) of off-white solid. mp 127.5–128.7 °C; ^1^H NMR (400 MHz, CDCl_3_) *δ* = 0.80 (t, *J* = 7.4, 3H), 1.98 (q, *J* = 7.3, 2H), 3.18 (d, *J* = 15.8, 2H), 3.56 (d, *J* = 15.8, 2H), 7.10–7.18 (m, 4H), 7.94 (s, 1H), 13.48 (br s, 1H); ^13^C NMR (101 MHz, CDCl_3_) *δ* = 9.9, 33.3, 43.7, 48.5, 124.6, 126.7, 141.5, 147.8, 165.3; *ν*_max_ 3114 (w), 3046 (w), 2956 (vs), 2844 (s), 2667 (br), 1500 (m), 1270 (s), 988 (s), 869 (s), 751 (vs) cm^–1^; ESI-HRMS: *m*/*z* = 214.1330 (MH^+^); C_13_H_16_N_3_ requires: *m*/*z* = 214.1339 (MH^+^); Purity: UPLC (254 nm): *t*_r_ = 1.933 min, 95.3% total area.

#### 4.1.15 3-(2-Ethylindan-2-yl)-N,N-dimethyl-1H-1,2,4-triazole-1-carboxamide (**17**)

3-(2-Ethylindan-2-yl)-1*H*-1,2,4-triazole **16** (48 mg, 0.225 mmol), DIPEA (5.0 eq., 196 μL), and *N*,*N*-dimethylcarbamoyl chloride (5.0 eq., 104 μL) in MeCN (3 mL) were stirred under Ar for 6 h at 60 °C. The solvent was removed *in vacuo* and 3-(2-ethylindan-2-yl)-*N*,*N*-dimethyl-1H-1,2,4-triazole-1-carboxamide **17** isolated by column chromatography on silica (1. PE/EtOAc = 2:1; 2. EtOAc). Yield: 32 mg (0.113 mmol, 50%) of beige solid. mp 68.1–69.6 °C; ^1^H NMR (400 MHz, CDCl_3_) *δ* = 0.78 (t, *J* = 7.4, 3H), 1.97 (q, *J* = 7.4, 2H), 3.10 (d, *J* = 15.7, 2H), 3.17 (s, 6H), 3.58 (d, *J* = 15.7, 2H), 7.09–7.13 (m, 2H), 7.16–7.20 (m, 2H), 8.68 (s, 1H); ^13^C NMR (101 MHz, CDCl_3_) *δ* = 9.9, 32.9, 38.8 (br), 43.5, 49.4, 124.5, 126.4, 142.2, 146.7, 150.1, 170.1; *ν*_max_ 3123 (w), 2959 (m), 2923 (m), 2850 (w), 1709 (vs), 1516 (s), 1481 (s), 1394 (s), 1270 (s), 1193 (s), 1002 (s), 743 (s), 694 (s) cm^–1^; ESI-HRMS: *m*/*z* = 285.1698 (MH^+^); C_16_H_21_N_4_O requires: *m*/*z* = 285.1710 (MH^+^); Purity: UPLC (220 nm): *t*_r_ = 3.337 min, 96.7% total area.

### 4.2 Physicochemical properties

For the aqueous stability determination, UPLC analyses were performed as described under Chemistry–General Information. The method used a Waters Acquity UPLC® HSS C18 SB column (2.1 mm× 50 mm, 1.8 μm) thermostated at 40 °C, with: injection volume, 5 μL; flow rate, 0.4 mL/min; detector *λ*, 220 nm; mobile phase A: 0.1% TFA (*v*/*v*) in MilliQ water; mobile phase B: MeCN. Gradient: 0–5 min, 20%–100% B; 5–6 min, 20% B.

Compound stock solutions (100 mmol/L) were prepared in absolute EtOH, while the internal standard (caffeine) was dissolved in PBS (0.1 mg/mL). The aqueous stability solutions were prepared by mixing 1200 μL PBS with internal standard, 150 μL absolute EtOH, and 150 μL compound stock solution in a HPLC vial to give 10 mmol/L compound concentration in PBS/EtOH = 80:20, which was injected immediately and then at hourly intervals for 48 h. The analyte AUCs were normalized on internal standard AUCs and compared with the timepoint zero. For the rate calculation, pseudo-first order kinetics were assumed.

For investigation of compound **8** stability in an aqueous medium, its stock solution (10,000 mg/L) was prepared in EtOH. 15 μL of the stock solution was mixed with 60 μL of caffeine (2500 mg/L in EtOH) and 1425 μL of the respective aqueous solution/buffer to achieve final concentration of 100 mg/L with 5% ethanol content. The UPLC analysis was carried as described above.

Thermodynamic solubility measurements were done in triplicates: compound **8** (approx. 5 mg) was weighed into Eppendorf tubes, 1 mL of the investigated solvent was added, and the suspension was shaken at room temperature for 24 h, followed by centrifugation at room temperature, 18,000 × *g* for 20 min. The supernatants were diluted 1:5 with 50% MeCN or used directly for UPLC analysis (calibration curve was constructed from 0.5–100 mg/L standard solutions of compound **8**).

Shake-flask partition coefficient measurement was done according to OECD guideline 107 (27. 7. 1995) and EPA OPPTS 830.7550 guideline (1996). Briefly, PBS pH 7.4 buffer (50 mL) and *n*-octanol (50 mL) were thoroughly shaken to ensure saturation of both phases and left to separate. Compound **8** stock solution in *n*-octanol (approx. 1000 mg/L) was accurately pipetted into Eppendorf tubes (500, 1000, 500 μL, respectively) and *n*-octanol-saturated PBS pH 7.4 buffer (500, 500, 1000 μL, respectively) was added. Each solvent ratio was done in duplicates. The Eppendorf tubes were thoroughly shaken to ensure partition and centrifuged (5000 × *g*, 10 min) to separate the phases. Water phase (carefully sampled using needle and syringe) was transferred to HPLC vials directly, whereas *n*-octanol phases were diluted with MeCN (10–50×) prior to analysis. Additional dilutions were carried out, if needed. Based on the concentrations of **8** in both phases, average log*P* was calculated. For Log*D* determinations, the above procedure was repeated using PBS buffers that were adjusted with 10% hydrochloric acid or 10% sodium hydroxide to pH 1, 2, 3, 4, 6, and 10 (the actual pH of the buffers was measured again after *n*-octanol saturation).

### 4.3 Computational studies

Computational experiments were performed on workstations at the Department of Pharmaceutical Chemistry, Faculty of Pharmacy and on Ažman HPC center at the National Institute of Chemistry in Ljubljana using Schrödinger Small Molecule Discovery Suite Release 2021-1 (Schrödinger, LLC., NY, USA, 2021) and Desmond/Maestro Non-commercial Distribution (Desmond v6.5, D. E. Shaw Research, New York, NY, USA, 2021).^84^ The 6KUX (resolution 2.7 Å, downloaded from OPM database^85^^,^^86^) antagonist-bound *α*_2A_ adrenoreceptor^25^ and 8QTX (resolution 2.1 Å) hBChE crystal structures were prepared using Protein Preparation Wizard^87^: bond orders were assigned using CCD database, missing hydrogens were added, disulfide bonds were created, termini were capped, missing side chains and loops were modelled with Prime^88^, and het protonation states (pH 7.0±2.0) were modelled with Epik^89^. All hets, cosolvent molecules, and fusion partners, except the co-crystallized ligands were removed. The waters present in the crystal structure were retained. Hydrogen bonds were automatically assigned and optimized using PROPKA^90^ (pH 7.0).

#### 4.3.1 Noncovalent molecular docking

For noncovalent docking calculations, the two prepared crystal structures defined above were used for each respective target. Additionally, all water molecules and ions were removed. The receptor grids were generated using Receptor Grid Generation with van der Waals radii scaling by 1, partial charge cut-off 0.25 (default settings), and OPLS_2005 forcefield^91^^,^^92^, while the co-crystallized ligand defined the centroid and size of the active site. For the first receptor grid, used for docking of compounds to hBChE, the innerbox of 12 Å^3^ and outerbox of 32 Å^3^ were defined. For the second grid, used to prepare pre-reaction poses of carbamoylazoles in hBChE, the settings were kept the same, except amide nitrogens of Gly116, Gly117, and Ala199 were also defined as hydrogen donors, and Ser198 was mutated to Ala198 to alleviate steric clash. The third grid, used to dock ligands to the *α*_2A_ adrenoreceptor, had the innerbox of 10 Å^3^ and the outerbox of 25.9 Å^3^.

Ligand structures were prepared with LigPrep and ionized with Epik^89^ (pH 7.0±2.0) using OPLS4 force field^93^. Docking was performed using Glide XP^94^ software by applying the default settings in docking of all compounds. No constraints were used, except for carbamoylazole pre-reaction pose prediction, where the constraint of at least 2 hydrogen bonds being formed with the oxyanion hole residues (as defined in the grid 2) had to be satisfied. The output poses were visualized and analysed with Maestro software.

#### 4.3.2 Covalent molecular docking

The CovDock protocol^95^ was used for covalent docking of synthesized carbamoylazoles in hBChE active site. In the above defined crystal structure, His438 residue was manually modified to the charged Hip438 to ensure a proper postreaction structure, and Ser198 was negatively ionized. Ser198 was selected as the reactive residue, and the box was defined around the centroid of the co-crystallized ligand with the size defined by the ligand (innerbox of 10.0 Å^3^ and outerbox of 24.1 Å^3^). Reaction type was customized using a custom chemistry file (see below) to produce a negatively charged tetrahedral intermediate. No constraints were imposed and the thorough pose prediction docking mode was used. The obtained docking poses with the “ex-carbonyl” anionic oxygen located outside the oxyanion hole were removed, and the remaining poses ranked by score and favourable interactions.

**Table:** CovDock custom chemistry file

REACTION_NAME	Custom
CUSTOM_CHEMISTRY	('<1>', ('charge', 0, 1))
CUSTOM_CHEMISTRY	('<1>|<2>', ('bond', 1, (1, 2)))
CUSTOM_CHEMISTRY	('<2>[O,S]', ('charge', -1, 2))
LIGAND_SMARTS_PATTERN	1,[C,c] = [O,S]
RECEPTOR_SMARTS_PATTERN	2,[CH2X4]-[O]

#### 4.3.3 Molecular dynamics simulations

The docking poses in hBChE were transferred back to the original crystal structure with crystal waters present (the pre-grid generation crystal structure, same alignment), while poses in *α*_2A_ were used directly. The systems for molecular dynamics (MD) simulations were prepared with System Builder: TIP4P^96^ water molecules were added up to 10 Å from the protein surface to solvate the protein in a orthorombic box, Na^+^ and Cl^–^ ions were added to neutralize the system and produce the final 0.15 mol/L concentration, and OPLS_2005 force field^91^^,^^92^ was used for parametrization of the macromolecule as well as the ligand. For *α*_2A_ 6KUX-derived structures, the protein was first placed automatically^86^ within a 1-palmitoyl-2-oleoyl-sn-glycero-3-phosphocholine (POPC) bilayer.

The default Desmond relaxation protocol (desmond_npt_relax.msj) was used for the equilibration stage of the ligand–hBChE complexes: (1) 100 ps of Brownian Dynamics NVT, 10 K, small timesteps, with restraints on the solute heavy atoms, (2) 12 ps NVT, 10 K, with small timesteps and restraints on the solute heavy atoms, (3) 12 ps NPT, 10 K, and restraints on the solute heavy atoms, (4) 24 ps unrestrained NPT; followed by the production stage. The default Desmond relaxation protocol for membrane-containing systems was used for the equilibration of ligand–*α*_2A_ complexes: (1) 50 ps of Brownian Dynamics NVT, 10 K, with restraints on the solute (50 kcal/mol/Å^2^); (2) 20 ps of Brownian Dynamics NVT, 100 K, pressure 1000 bar, with restraints on the solute and membrane heavy atoms (50 kcal/mol/Å^2^); (3) 100 ps of NP*γ*T, 100 K, pressure 1000 bar, with restraints on the solute heavy atoms (10 kcal/mol/Å^2^) and on the membrane N and P atoms (only z direction, 2 kcal/mol/Å^2^); (4) 150 ps of NP*γ*T, heating from 100 to 300 K, pressure 100 bar, starting with restraints on the solute heavy atoms (10 kcal/mol/Å^2^) and on the membrane N and P atoms (only in the z direction, 2 kcal/mol/Å^2^), and gradually reducing the restraints force constants to 0; (5) 50 ps of NVT, 300 K, with restraints on the protein backbone and ligand heavy atoms (5 kcal/mol/Å^2^); (6) 50 ps of NVT, 300 K, without restraints; followed by the production stage.

The following setup was used for the MD production stage: 1.2 ps interval for energy, RESPA (reference system propagator algorithm) integrator with 2 fs time step, cutoff scheme at 9.0 Å, random seed, isothermal-isobaric NPT ensemble at 300 K and 1.013 bar pressure with Nose-Hoover chain thermostat and Martyna-Tobias-Klein barostat (1 and 2 ps relaxation time, respectively, isotropic coupling) for BChE structures and NP*γ*T ensemble^97^ with surface tension of 4000 bar Å^–1^ for *α*_2A_ structures. The simulation times were 20–1000 ns with 1000 frames per trajectory saved. The simulation results were analyzed using the built-in Desmond tools.

### 4.4 Biology

#### 4.4.1 ChE–IC_50_ determination and binding kinetics

The inhibitory potencies of the compounds against the ChEs were determined using the method of Ellman following the procedure described previously^21^. Briefly, compound stock solutions in DMSO were incubated with Ellman’s reagent and ChEs (final concentrations: 370 μmol/L Ellman’s reagent, approximately 1 nmol/L or 50 pmol/L hBChE or hAChE, respectively) in 0.1 mol/L sodium phosphate pH 8.0 for 5 min at 20 °C. For time-dependency measurements, the pre-incubation time was varied (1, 5, 15, 30 or 60 min). The reactions were started by the addition of the substrate (final concentration, 500 μmol/L butyrylthiocholine iodide or acetylthiocholine iodide for hBChE and hAChE, respectively). The final content of DMSO was always 1%. The increase in absorbance at 412 nm was monitored for 2 min using a 96-well microplate reader (Synergy HT, BioTek Instruments, VT, USA). The initial velocities in the presence (*v*_*i*_) and absence (*v*_0_) of the test compounds were calculated. The inhibitory potencies were expressed as the residual activities, according to Eq. (1):

(1) RA = *v*_*i*_ / *v*_0_

For IC_50_ determinations, at least seven different concentrations for each compound were used. The IC_50_ values were obtained by plotting the residual ChE activities against the applied inhibitor concentrations, with the experimental data fitted to a four-parameter logistic function (GraphPad Prism 9.4). Tacrine and donepezil were used as positive controls.

The progress curves for the time courses of product formation for the hydrolysis of butyrylthiocholine iodide were measured on an Agilent Cary 3500 UV–VIS spectrophotometer with Compact Peltier (thermostated at 25 °C) at 412 nm using a 0.6 mL cuvette (optical path–0.5 cm). 30 mmol/L Tris buffer pH 7.0 with 1 mg/mL BSA and 0.02% NaN_3_ was used and all dilutions were done in glass vessels (note: dilute hBChE solutions rapidly lose activity, presumably due to adsorption to plastic, preventing meaningful decarbamoylation assays. In our hands, under the above conditions only, the diluted and stock solutions of hBChE were stable for at least a month at room temperature.). Total volume in the cuvette was always 500 μL, and enzyme solutions that gave upon dilution *v*_0_ = (d*A*/d*t*) ≈ 0.5 (for carbamoylation) and ≈ 1 for (for decarbamoylation) were used. For carbamoylation experiments, 0.5–1 μL of enzyme stock, 5 μL of compound solution, and 484 μL of buffer were preincubated for the specified time, then 10 μL of the mixture of 25 mmol/L Ellman’s reagent (DTNB) and 20 mmol/L butyrylthiocholine (large excess to prevent substrate depletion) were added, quickly mixed, and measurement started. For decarbamoylation experiments, 50 μL of the enzyme stock solution and 0.25–0.5 μL of the compound solution in MeOH (concentration chosen to achieve 85%–95% inhibition, typically 0.5–1 mmol/L) were incubated for 1 h at room temperature, then the mixture was diluted 1:1000 with the buffer, and 500 μL aliquots were drawn at different timepoint, mixed with 10 μL of the mixture of 25 mmol/L Ellman’s reagent (DTNB) and 20 mmol/L butyrylthiocholine iodide, and hBChE activity assayed. The data analysis followed the literature^63^^,^^98^.

#### 4.4.2 α_2A_ binding assay

The ligand binding assay on human *α*_2A_ adrenoreceptor from CHO cells using antagonist radioligand [^3^H]RX821002 was outsourced to Eurofins (assay number 13, study ID FR095-0034701). In each experiment the respective reference compound (yohimbine) was tested concurrently with the test compounds, and the data were compared with historical values determined at Eurofins. The experiment was accepted in accordance with Eurofins validation Standard Operating Procedure. Inhibition higher than 50% is considered to represent significant effects of the test compounds. The inhibition constants (*K*_i_) were calculated using the Cheng-Prusoff equation.

#### 4.4.3 *In vitro* cellular assays

*Cell culture and treatments* The human neuroblastoma SH-SY5Y cell line was purchased from American Type Culture Collection (Manassas, VA, USA). Cells were cultured in Advanced Dulbecco’s modified Eagle’s medium (Gibco, Thermo Fisher Scientific, Waltham, MA, USA) supplemented with 10% fetal bovine serum (FBS, Gibco), 2 mmol/L l-glutamine, 50 U/mL penicillin and 50 μg/mL streptomycin (Sigma, St. Louis, MO, USA) in a humidified atmosphere of 95% air and 5% CO_2_ at 37 °C, and grown to 80% confluence. Prior to cell treatment, complete medium was replaced with serum-reduced medium (*i.e.*, with 2% FBS). Compounds were prepared as a stock solution of 50 mmol/L in DMSO.

*MTS assay* SH-SY5Y cells were seeded in 96-well plates (10^4^/well) and assessed by MTS ([3-(4,5-dimethylthiazol-2-yl)-5-(3-carboxymethoxyphenyl)-2-(4-sulfophenyl)-2*H*-tetrazolium, inner salt) assay for their response to compounds. Cells were treated with increasing concentrations of compounds (0.1–10 μmol/L for **5**, 1–100 μmol/L for **6**–**8**) in a serum-reduced medium, and metabolic activity was assessed after 24 h (**5**) or 48 h (**6**–**8**) using the CellTiter 96® Aqueous One Solution Cell Proliferation Assay (Promega, Madison, WI, USA), in accordance with the manufacturer’s instructions. Absorbance was measured with an automatic microplate reader (Tecan Safire^2^, Switzerland) at 492 nm. Results are presented as a percentage of the control (DMSO).

*Toxicity assay* The toxic profile of **6** was determined with the fluorescent intercalator 7-aminoactinomycin D (7-AAD) assessed by flow cytometry. SH-SY5Y cells were seeded in 24-well culture plates (2 × 10^4^/well) and on the next day treated with **6**. After 48 h, cells were harvested and washed in cold phosphate-buffered saline (PBS), and labelled with 7AAD (2 μg/mL; Sigma–Aldrich) for 10 min at room temperature. Cells were then analysed for cytotoxicity by flow cytometry on Attune NxT flow cytometer (Thermo Fisher Scientific). The percentage of 7AAD positive (7AAD^pos^) cells was evaluated using FlowJo software (FlowJo, LLC., Ashland, OR, USA) and recorded as relative to control cells.

*Neuroprotection assay* The neuroprotective effect of compounds on cytotoxic effect of A*β*_1–42_ was determined with the fluorescent intercalator 7-aminoactinomycin D (7-AAD) assessed by flow cytometry. Prior to cell treatment, the peptide A*β*_1–42_ (Merck Millipore, Darmstadt, Germany) was dissolved in DMSO at a concentration of 1 mmol/L and was incubated at 100 μmol/L in 1 mol/L HCl at 37 °C for 24 h to induce A*β* aggregation. SH-Y5Y cells were seeded in 24-well culture plates (2 × 10^4^/well) and next day pre-treated with compounds at concentration 5 μmol/L for 1 h in serum-reduced medium, followed by exposure to aggregated 5 μmol/L A*β*_1–42_. After 48 h treatment, cells were harvested and washed in cold phosphate-buffered saline (PBS), and labelled with 7AAD (2 μg/mL; Sigma–Aldrich) for 10 min at room temperature. Cells were then analysed for cytotoxicity by flow cytometry on Attune NxT flow cytometer (Thermo Fisher Scientific). The percentage of 7AAD positive (7AADpos) cells was evaluated using FlowJo software (FlowJo, LLC., Ashland, OR, USA).

*Caspase-3 activity and Western blot analysis* The activity of caspases-3 was determined in total cell lysates of the SH-SY5Y cells treated as described. Cells were seeded into 6-well culture plates at a density of 5 × 10^5^ cells per well. Following cell treatment for 24 h, cell lysates were prepared, and the caspase-3 activity was assessed using the fluorescent peptide substrate *N*-acetyl-l-*α*-aspartyl-l-*α*-glutamyl-l-valyl-*N*-[2-oxo-4-(trifluoromethyl)-2*H*-1-benzopyran-7-yl]-l-*α*-asparagine (Ac-DEVD-AFC; Bachem). Cell lysates (20 μg of protein) were incubated for 30 min at 37 °C in caspase reaction buffer (20 mmol/L PIPES, pH 7.2, 10% sucrose, 0.1% CHAPS, 1 mmol/L EDTA, 100 mmol/L NaCl) followed by the addition of 100 μmol/L Ac-DEVD-AFC peptide substrate. Immediately after the addition of the substrate, fluorescence was monitored continuously for 30 min at an excitation wavelength of 405 nm and an emission wavelength of 535 nm using a Tecan Safire^2^ fluorescence microplate reader. Data are presented as changes in fluorescence over time (Δ*F*/Δ*t*), with caspase-3 activity expressed as DEVDase activity relative to control cells treated with DMSO alone.

Likewise, protein levels of phosphorylated and total protein levels of Tau were determined by Western blotting. SH-SY5Y cells were seeded into 6-well culture plates at a density of 5 × 10^5^ cells per well. Following cell treatment, cell lysates were prepared using cell lysis buffer (50 mmol/L HEPES (pH 6.5), 150 mmol/L NaCl, 1 mmol/L EDTA, 1% Triton X-100) supplemented with a cocktail of protease and phosphatase inhibitors. Equal concentrations of protein from whole cell lysates (1 μg/μL) were denatured by the addition of SDS-PAGE buffer, heated for 5 min, and resolved by SDS-PAGE on 12% Tris-glycine gels. The proteins were then transferred to PVDF membranes using iBlot (Thermo Fisher Scientific). Membranes were blocked in 5% (*w*/*v*) non-fat dried milk powder in Tris-buffered saline with Tween 20 (TBST; 20 mmol/L Tris/HCl, 137 mmol/L NaCl, pH 7.4, and 0.1% Tween 20) at RT for 1 h. Subsequently, the membranes were incubated overnight at 4 °C with primary rabbit anti-phosphorylated Tau (1:500; 28866-1-AP; ProteinTech) and after with the stripping buffer (100 mmol/L 2-mercaptoethanol, 2% SDS, and 62.5 mmol/L Tris/HCl, pH 5.7) for 1 h at 65 °C with rabbit-Tau (1:2000; 10274-1-AP; ProteinTech) in TBST containing 3% (*w*/*v*) bovine serum albumin (A2153; Sigma–Aldrich). After washing, secondary horseradish peroxidase-conjugated anti-rabbit (1:5000; 111-035-045; Jackson ImmunoResearch) antibodies were added in TBST containing 5% (*w*/*v*) non-fat dried milk powder for 1 h. After washing, protein bands were visualized with enhanced chemiluminescence detection kits (34075 or 34095; Thermo Fisher Scientific) and recorded with a G: Box imager (Syngene, UK). Band intensities were quantified using Gene Tools software (Sygene). Values are expressed as a ratio of phosphorylated to total protein level and are normalized to the control sample.

#### 4.4.4 Acute *in vivo* toxicity

All mice used were 9–15 weeks old wild-type BALB/c mice bred in the Laboratory of Animal Genomics at the Faculty of Veterinary Medicine, University of Ljubljana. For intravenous administration, 4 males and one female were additionally purchased from Envigo RMS Holding Corp. Mice were conventionally bred in open polycarbonate cages (Tecniplast, Buguggiate, Italy) and provided with wood chip bedding (Lignocel®, Rettenmaier and Sons, Germany). Mice were kept under standard conditions, *i.e.*, a 12:12 dark–light cycle (LED, CCT 3000 K, ≤ 25 lux above the cage), at temperature 21–24 °C, and 50%–60% humidity. They were fed irradiated rodent diet with minimal levels of phytoestrogens (Teklad global 16% protein rodent diet®, BN 2916, Envigo, Udine, Italy) and had access to drinkable tap water ad libitum. The *in vivo* study was evaluated by the National Ethics Committee for Animal Experiments and approved by the Veterinary Administration of the Republic of Slovenia; license number document no. U34401-10/2021/8, U34401-10/2021/13, and U34401-10/2021/1.

According to the OECD guidelines for acute toxicity testing^99^, compound **8** was initially tested at 10 mg/kg dosage (10-fold the intended therapeutic dose (1 mg/kg)) in 3 female mice and repeated on 3 additional animals. Compound **8** was dissolved in DMSO at 100 mg/mL and then just prior to the administration diluted 100-fold in 1% Tween® 20 to final concentration of 1 mg/mL. Four hours before the administration of **8**, the animals had no access to food while water was available. After this fasting period, the animals were weighed and compound **8** was administered by oral gavage at 200 μL/20 g body weight using flexible plastic feeding tubes (20G × 30 mm, Instech Laboratories, USA). After administration, the animals were not fed for an additional hour. The mice were then observed regularly for the first 24 h, with particular attention paid to the first 4 h, and then daily for a total of 14 days, with attention paid to the changes in respiratory rate, heart rate, skin and fur, eyes and mucous membranes, behavioural patterns, and especially to the acute CNS toxicity symptoms (tremors, twitching, salivation, diarrhoea, lethargy, sleep, coma). At the end of the 14-day observation period, all experimental animals were euthanized with CO_2_ and cervical dislocation.

In the first 15–30 min after administration, the tail veins showed marked vasodilation, which gradually extended to all mucous membranes, including the nasal, vaginal, anal, and ear veins. This vasodilation is consistent with *α*_2_ adrenoreceptor antagonism mediated by atipamezole liberated from **8**. Additionally, tachypnea and tachycardia were observed, and there was a striking change in the behaviour, *i.e.*, an increased activity and irritability, as evidenced by running, climbing on poles, excessive burrowing, and grooming behaviour. As soon as food was made available to the mice, they fed immediately. This behaviour is most likely due to the CNS excitation due to atipamezole^42^^,^^100^. After 4 to 5 h, all effects subsided. Since **8** does not inhibit hAChE, the observed vasodilation and hyperreactivity are most likely caused by atipamezole alone. No other acute cholinergic side effects, such as salivation or tremors, were observed^66^. All animals survived the experiment. In addition, no such effects were observed at 1 mg/kg dose.

#### 4.4.5 Cholinesterase inhibition upon repeated dosing

The 12 mice used in this pilot study were 9–14 months old male wild-type C57BL/6JRccHsd mice bred in the Laboratory of Animal Genomics at the Faculty of Veterinary Medicine, University of Ljubljana, or purchased from Envigo RMS Holding Corp. Mice were conventionally bred in open polycarbonate cages (Tecniplast, Buguggiate, Italy) and provided with wood chip bedding (Lignocel®, Rettenmaier and Sons, Germany). Mice were kept under standard conditions, including a 12:12 dark–light cycle (LED, CCT 3000 k, with no more than 25 lux above the cage), a temperature range of 21–24 °C, and humidity between 50%–60%. They were fed irradiated rodent diet (Teklad global 16% protein rodent diet®, BN 2916, Envigo, Udine, Italy) and had access to drinkable tap water ad libitum. Due to the nature of this pilot study, the mice were housed individually. The experimental design was evaluated by the National Ethics Committee for Animal Experiments and approved by the Veterinary Administration of the Republic of Slovenia (document no. U34401-10/2021/8, with amendments U34401-10/2021/13 and U34401-10/2021/1).

Before the start of study, blood was drawn from the submandibular vein (facial vein) of the animals under isoflurane inhalation anesthesia (Isoflurin®, VetPharma, Spain) using a 5 mm lancet (Goldenrod Animal Sterile, MEDIpoint, Inc., NY, USA), collected in a microtube (Eppendorf, Safe-Lock tube, 0.5 mL, Eppendorf AG, Germany) and allowed to clot. After approximately 1 h, the blood was centrifuged at 6000 × *g* for 15 min at 4 °C to obtain the sera, which were stored at –80 °C until analysis.

Seven animals received the 1 mg/kg dose of compound **8**, and five animals received the 5 mg/kg dose of compound **8**. Compound **8** was prepared as a stock solution in 100% MeCN (5 and 10 mg/mL) and kept refrigerated for the duration of the study. The dosages were prepared as follows: based on the animal’s body weight, a calculated volume of compound **8** stock solution was accurately pipetted onto a cubic piece (approx. 50 mg) of sterilized toast (Ölz multigrain toast, Rudolf Ölz Meisterbäcker GmbH & Co KG, Austria), and left to dry at room temperature for 5 min. Subsequently, 25 μL of 50% sucrose in distilled water were added at the same site to mask the bitter taste of compound **8**. These dosage units were prepared twice a week during the study and stored in a closed glass petri dish at room temperature until use. For administration, the toast pieces were placed on the floor of the cage at the same time each day, and left for the animal to eat it voluntarily (the consumption of the dosage was confirmed by observation). Compound **8** was administered in this way for 27 days, eliminating the need for repeated handling and injection. The animals were observed daily for general activity level, hair coat quality or other abnormal behavior or clinical signs. No adverse effects were observed at either dosage.

24 h after the last dose was administered, the mice were humanly euthanized under isoflurane inhalation anesthesia (Isoflurin®, VetPharma, Spain) and the blood was withdrawn from the left ventricle using a syringe, collected in a microtube (Eppendorf, Safe-Lock tube, 0.5 mL, Eppendorf AG, Germany) and allowed to clot. After approximately 1 h, the blood was centrifuged at 6000 × *g* for 15 min at 4 °C to obtain the sera, which were stored at –80 °C until analysis. Cholinesterase activities in murine plasma after repeated dosing of compound **8** were determined using modified literature procedure^101^^,^^102^: initial velocities for hydrolysis of butyryl- or acetylthiocholine iodide were measured on an Agilent Cary 3500 UV–Vis spectrophotometer with Compact Peltier (thermostated at 25 °C) at 412 nm using a 1.6 mL cuvette (optical path–1.0 cm). Animal sera (5–10 μL) were diluted in 0.5 mmol/L DTNB in 0.1 mol/L sodium phosphate buffer pH 7.4 (total volume 1000 μL), substrate (5 μL of 100 mmol/L, final concentration 0.5 mmol/L) was added, and the increase in absorbance monitored for 3 min. BChE activity was determined using butyrylthiocholine iodide, while AChE activity was determined on isoOMPA-inhibited sera (buffer-diluted sera were preincubated for 25 min with 1 μL of 100 mmol/L isoOMPA–0.1 mmol/L final concentration) using acetylthiocholine iodide (final concentration 0.5 mmol/L). Measurements were performed in triplicate and pairwise compared before and after treatment for each subject (*n* = 5 per group). For calculation, extinction coefficient of 14150 L mol^–1^ cm^–1^ was used and activity given in U/mL (1 U = 1 μmol of butyryl- or acetylthiocholine iodide hydrolysed per min).

#### 4.4.6 Pharmacokinetics in mice and in human plasma

*Animals* All mice used were 9–15 weeks old wild-type BALB/c mice bred in the Laboratory of Animal Genomics at the Faculty of Veterinary Medicine, University of Ljubljana. For intravenous administration, 4 males and one female were additionally purchased from Envigo RMS Holding Corp. Mice were conventionally bred in open polycarbonate cages (Tecniplast, Buguggiate, Italy) and provided with wood chip bedding (Lignocel®, Rettenmaier and Sons, Germany). Mice were kept under standard conditions, *i.e.*, a 12:12 dark–light cycle (LED, CCT 3000 K, ≤ 25 lux above the cage), at temperature 21–24 °C, and 50%–60% humidity. They were fed irradiated rodent diet with minimal levels of phytoestrogens (Teklad global 16% protein rodent diet®, BN 2916, Envigo, Udine, Italy) and had access to drinkable tap water ad libitum. The *in vivo* study was evaluated by the National Ethics Committee for Animal Experiments and approved by the Veterinary Administration of the Republic of Slovenia; license number document Nos: U34401-10/2021/8, U34401-10/2021/13, and U34401-10/2021/1. All animal experiments and studies were compliant with and followed the ARRIVE guidelines, U.K. Animals (Scientific Procedures) Act, 1986 and associated guidelines, EU Directive 2010/63/EU for animal experiments, National Research Council's Guide for the Care and Use of Laboratory Animals, and other relevant laws and institutional guidelines.

*Plasma and brain distribution profile* To measure plasma and brain distribution profile after intravenous and peroral administration of compound **8**, mice were sacrificed at six time points (0.25, 0.5, 1, 2, 4, and 12 h) after administration. For each time point, 4 mice, two females and two males, were used. A total of 24 animals were used (*n* = 24) for each route of administration. The compound **8** (1 mg/mL) was prepared and administered as described under 4.4.4. Peroral administration was performed *via* flexible plastic feeding tubes (20G × 30 mm, Instech Laboratories, USA) by gavage, with volume dose of 200 μL/20 g body weight. Intravenous administration into the tail vein was performed under isoflurane inhalation anesthesia (Isoflurin®, VetPharma, Spain) using a syringe (INSULIN100, 29G × 1/2″, 12.7 mm, CHINS00329, Chirana T. Injecta, a.s., Slovakia) with a volume dose of 150 μL. At each timepoint, mice were anesthetized and euthanized with isoflurane (Isoflurin®, VetPharma, Spain), and blood was collected by syringe from the left ventricle in a heparinized tube (Microtube 1.3 mL, Sarstedt AG & Co. KG, Germany), centrifuged at 3000 × *g* for 15 min at 4 °C, and plasma was separated. The bodies were perfused with PBS buffer (PBS tablet, Sigma–Aldrich Chemie GmbH, Germany), the brains removed, and flash frozen in liquid nitrogen. Both plasma and brain samples were then stored at –80 °C until analysis.

LC–HRMS analyses were performed on Thermo Scientific Q Exactive Plus LC–MS/MS and Thermo Scientific Dionex UltiMate 3000 modular system (Thermo Fisher Scientific Inc.), using a Waters Acquity UPLC® BEH C18 column (2.1 mm × 50 mm, 1.7 μm) and Waters Vanguard UPLC® BEH C18 precolumn (2.1 mm× 5 mm, 1.7 μm), thermostated at 40 °C, with: flow rate, 0.4 mL/min; autosampler temperature: 5 °C, mobile phase A: 0.1% formic acid in MQ water; B: 0.1% formic acid in MeCN. Compound stock solutions (10 mmol/L or 1000 mg/L) were prepared in MeOH. Five replicates of different standard concentrations were injected to check the precision and accuracy was checked by injecting 2 samples of known analyte concentration. For compound pair **5** and **8**: detector: *t*-SIM (runtime 1.4–3.4 min, positive mode, resolution: 70,000; AGC target: 5e4; maximum IT: 200 ms; isolation window: 2 *m*/*z*), injection volume: 10 μL, gradient: 0–3.8 min, 20%–95% B; 3.8–4.0 min 95% B; 4.0–4.1 min 95%–20% B; 4.1–6.0 min 20% B. Processing: ICIS peak detection algorithm, analyte **5**, expected retention time: 1.76 min, 30 s window, mass range 213.13862; analyte **5**-*d*_5_–expected retention time: 1.78 min, 30 s window, mass range 218.17001; analyte **8**, expected retention time: 2.88 min, 30 s window, mass range 312.20704; analyte **8**-*d*_5_, expected retention time: 2.88 min, 30 s window, mass range 317.23842. See Supporting Information Tables S4.

To monitor the progress of hydrolysis of **8** in human plasma, human plasma (499 μL, anticoagulant: K_2_EDTA) was treated with 1 μL of 10,000 μg/L **8** solution to produce final concentration of 20 μg/L, and incubated at 37 °C for 24 h. 50 μL aliquots were drawn successively at different timepoints, 1 μL of 2 500 μg/L of **5**-*d*_5_ and **8**-*d*_5_ internal standard solution and 199 μL MeOH were added, vortexed, centrifuged at 4 °C, 10,000 × *g* for 15 min, the supernatant transferred to vials, and injected. Calibration curve was constructed from standard solutions of **5** and **8** in human plasma (0.01–30 μg/L), prepared as described above.

Plasma from treated mice was thawed at room temperature, 50 μL aliquots were taken, 1 μL of internal standard solution (1000 μg/L **5**-*d*_5_ and **8**-*d*_5_) and 199 μL MeOH were added, vortexed, centrifuged at 4 °C, 10,000 × *g* for 15 min, the supernatants transferred to vials, and injected. For plasma calibration curve samples, isoOMPA (20 mmol/L DMSO solution, final concentration: 100 μmol/L), was first added to plasma from untreated mice and left at room temperature for 15 min. The highest concentration **5** and **8** standard solution (100 μg/L) was then prepared in isoOMPA-inhibited plasma, and then serially diluted with isoOMPA-inhibited plasma to produce calibration standard samples (0.001–100 μg/L). 50 μL of the respective plasma sample and 1 μL of internal standard solution (1000 μg/L **5**-*d*_5_ and **8**-*d*_5_) were then diluted with 199 μL MeOH, vortexed, centrifuged at 4 °C, 10,000 × *g* for 15 min, the supernatant transferred to vials, and injected. To determine concentrations of **5** and **8** in mouse brain tissue, whole mouse brains were weighed frozen, thawed on room temperature, and homogenized using razor blade and spatula. Aliquots (approx. 100 mg) were weighed, 1000 μL of 100 mmol/L NaOAc buffer (pH 5) were added, followed by 5 μL of 20 mmol/L isoOMPA in DMSO, and 1 μL of internal standard solution (1000 μg/L **5**-*d*_5_ and **8**-*d*_5_), vortexed for 15 seconds, sonicated for 30 min, and centrifuged at 4 °C, 10,000 × *g* for 15 min. The supernatants were loaded onto Strata™-X-Drug B (30 mg, 33 μm, cation mixed-mode polymer sorbent) SPE column, slowly passed through, the columns successively washed with 2 × 1000 μL of 100 mmol/L NaOAc buffer (pH 5), dried under vacuum for 15 min, and eluted with 2 × 400 μL of elution buffer (DCM:4.2 mol/L Me_3_N in EtOH = 9:1). The solvent was removed under an air stream at ambient temperature, the samples were reconstituted in 100 μL of MeCN, loaded into vials, and injected. For brain calibration curve samples (0.1–100 μg/L), approx. 100 mg of untreated mouse brain tissue was weighed, spiked with appropriate amounts of **5** and **8** standard solutions, and treated in the same way as above.

#### 4.4.7 *In vivo* behavioural studies

All animal experiments were conducted in accordance with the regulations of Animal Ethics Committee of Chinese Academy of Medical Sciences and Materia Medica Research Institute. Animal studies were conducted under the guidance of the National Institutes of Health’s Guidelines for the Care and Use of Laboratory Animals and were approved by the Center for New Drug Evaluation and Research, China Pharmaceutical University (Nanjing, China), under approval number SYXK(Su)2023-0018. For the statistical analysis, Student’s *t*-test and One-way analysis of variance (ANOVA) with Tukey’s multiple comparison test were used, and the results reported as means±SD.

*Mice model with icv-injection of Aβ*_*1–42*_ The experiments were performed using ICR male mice (8–10 weeks old, weight 18–20 g), which were purchased from Shanghai Bikai Animal Breeding Factory (Shanghai, China). Animals were housed in cages (6 mice per cage) at a constant temperature of 25±2 °C, humidity of 55%±10% and a light/dark (12:12) cycle. The mice had unlimited access to food and water prior to the experiment. Specified conditions for the maintenance of mice were ensured throughout the experiments, including tree bedding (Yizheng Anlimao Biotechnology Co., Ltd., China) and lab feed (Zimao, China). For behavioural tests, mice were selected randomly, each group consisted of 6 mice. The experiments were performed between 9 am and 3 pm.

According to the literature procedure^103^, recombinant human hexafluoroisopropanol-pretreated A*β*_1–42_ from Merck Millipore (Darmstadt, Germany) was dissolved in 1% DMSO, diluted with saline to the final concentration of 2 mg/mL, and incubated for 72 h to form oligomers. Rivastigmine tartrate, compounds **7** and **8** were dissolved in DMSO (with sonication), diluted 50× in PEG400 (with sonication), and finally diluted 2× in saline (with sonication) prior to administration.

ICR mice were divided into 6 groups with *n* = 4 per group: (1) control group mice were not operated on and were just treated with normal saline (ip), (2) model group mice received oligomeric A*β*_1–42_ (10 μg/mouse, icv) and were treated with saline (ip), (3) sham operation group received saline (icv) and was treated with saline (ip), (4) positive control group received oligomeric A*β*_1–42_ (10 μg/mouse, icv) and was treated with rivastigmine **4** (1 mg/kg, ip), (5) experimental group that received oligomeric A*β*_1–42_ (10 μg/ mouse, icv) and was treated with compound **8** (10 mg/kg, ip), (6) experimental group that received oligomeric A*β*_1–42_ (10 μg/ mouse, icv) and was treated with compound **7** (10 mg/kg, ip).

During 17 days of once-a-day ip administration, behavioural experiments assaying cognitive function were carried out–Morris Water Maze experiment on Days 9–14, novel object recognition experiment on Day 15, and Y maze test on Day 16–one trial per day. Finally, the mice were sacrificed on Day 17, and sera and brain tissues were collected for subsequent analysis.

*Transgenic APP/PS1 mice model* The experiments were performed using C57BL/6J-TgN(APP/PS1)ZLFILAS male mice (6 months old, weight 20–22 g), which were purchased from Beijing HFK Bioscience Co., Ltd. (Beijing, China). This APP/PS1 double transgenic AD mice model expresses the Swedish mutation of human amyloid precursor protein (APP) and mutated human presenilin 1 (PS-1), and was established at the Institute of Laboratory Animal Sciences, Chinese Academy of Sciences by crossing PrP-hAPPK595N/M596L(Swe) single transgenic dementia model mice and PrP-hPS1dE9 single transgenic dementia model mice. Compared to APP single transgenic AD mice, APP/PS1 double transgenic AD mice have an earlier onset of A*β* deposition in the brain, higher A*β* load, more neurodegeneration, synaptic loss, and age-related neurological and behavioural functional disorders. The C57BL/6J-TgN(APP/PS1)ZLFILAS double transgenic dementia mice exhibit cognitive and behavioural changes starting at 3 months of age, senile plaques at 5 months of age, and many senile plaques at 12 months of age.

Animals were housed in cages (6 mice per cage) at a constant temperature of 25±2 °C, humidity of 55%±10% and a light/dark (12:12) cycle. The mice had unlimited access to food and water prior to the experiment. Specified conditions for the maintenance of mice were ensured throughout the experiments, including tree bedding (Yizheng Anlimao Biotechnology Co., Ltd., China) and lab feed (Zimao, China). For the experiments, mice were selected randomly.

APP/PS1 mice were divided into 5 groups: model group (*n* = 6, normal saline, ip), rivastigmine group (*n* = 6, 1 mg/kg rivastigmine, ip), atipamezole **5** group (*n* = 5, 0.8 mg/kg atipamezole hydrochloride–equimolar to 1 mg/kg **8**, ip), compound **8** groups (*n* = 7, 1 mg/kg of compound **8**, ip) and (*n* = 3, 5 mg/kg of compound **8**, ip). The experiments were performed between 9 am and 3 pm. During 15 days of once daily ip administration, behavioral experiments assaying cognitive function were carried out: Morris Water Maze experiment on Days 7–12, Y maze test on Day 13, and novel object recognition experiment on Day 14. After *in vivo* tests, the animals were immediately euthanized on Day 15, and sera and brain tissues were collected for subsequent analysis.

*Behavioural assays* The Morris water maze was placed in a lighted room at 25 °C, and it was divided into four equal quadrants. In the third quadrant of the water maze (120 cm in diameter and 60 cm in height) with a water depth of 40 cm, an escape platform (10 cm in diameter, 1 cm submerged under the water surface) was placed. One trial was performed per day, and the study consisted of five days of memory training, followed by the test with escape platform removed on the sixth day. The time spent searching for the missing platform was recorded by Xeye Aba image processing system (Beijing Zhongshi Dichuang Technology Development Co., Ltd.) and analysed by GraphPad Prism 5.

Novel object recognition experiment was performed in a maze with dimensions 50 cm×50 cm×40 cm, with black cuboid (4 cm×4 cm×5 cm) as the old object, and white cylinder (2*r* = 5 cm, *h* = 5 cm) as the new object. The experiment was divided into exploration stage and test stage, separated by 30 min interval. In the exploration phase, two objects were placed diagonally on the interior of the open field in an open space 5 cm from the wall. Then the mice were placed in the centre of the open field and allowed to explore in the open field for 10 min. At the end of the exploration, the mice were placed back in their cages. The environment and objects inside the open field were cleaned with 70% ethanol and wiped with paper towels to minimize olfactory cues and odour residues. The two familiar objects were then replaced with a duplicate object (making sure there were no remaining olfactory cues on the previously used object) and the other object was replaced with a new object, and placed in the same position, 5 cm away from the wall. Once again, the mice were placed in the centre of the open field and allowed to explore in the open field for 3 min. Time spent exploring new and old objects was recorded using ANY-maze software and processed using GraphPad Prism 5.

Spontaneous alternation (Y maze) test was used to assess working memory in mice. The dimensions of the maze were 50 cm arm length, height 20 cm, and center regular hexagonal side length 10 cm. The mice were placed in the middle of the three arms. The mice were allowed to shuttle freely within the three arms for 3 min. The experimental data recorded the number of times the animal entered the three arms in a row. Note that the spontaneous continuous alternate selection experiment is based on the novelty of the maze, and the experimental animals do not need to be familiar with the environment in advance. After each experimental animal completed the test, the insides of Y maze were cleaned with 70% ethanol and dried with paper towels to remove the odor of the previous animal subject. The behavior of mice in the Y maze was recorded by Xeye Aba and processed by GraphPad Prism 5.

*Western blotting* The Western blot experiments were performed as previously reported^103^: mice brains were combined, mixed with RIPA Buffer (NCM WB3100, containing protease phosphatase inhibitors, NCM P002), and ground into homogenate with a freezing grinder, and then centrifuged at 12,000 × *g* at 4 °C for 10 min. Supernatants were collected and stored at −80 °C until analysis. The protein concentration of the whole lysates was measured by the BCA assay (NCM WB6501) with a Varioskan Flash (Thermo, Waltham, MA) at 562 nm. The protein samples (containing 80 μg of proteins) were denatured with loading buffer (Beyotime P0015L), heated at 100 °C for 3–5 min, and then separated by SDS−PAGE (NCM P2012) and transferred to Immobilon-P Transfer membrane (Millipore, USA) by wet transfer system. After blocking with Blot Blocking buffer (NCM P30500) for 40 min, the membranes were incubated with primary antibodies in TBST (20 mmol/L Tris hydrochloride, 137 mmol/L NaCl, pH 7.4, and 0.1% Tween 20) at 4 °C overnight. After that, they were washed 3-times with TBST for 5 min, and then reacted with a horseradish peroxidase (HRP)-conjugated secondary antibody in TBST at 37 °C for 90 min. The primary antibodies used were anti-pTau (Thr181) (polyclonal, 28866-1-AP, Proteintech, IL, USA), anti-Tau (monoclonal, 66499-1-Ig, Proteintech, IL, USA), anti-pGSK3*β* (Ser9) (monoclonal, 5558T, Cell Signaling Technology, MA, USA), anti-GSK3*β* (monoclonal, 9315T, Cell Signaling Technology, MA, USA), and anti-GAPDH (AB56131, bioworlde, China). The secondary antibodies used in this study were goat anti-rabbit IgG (H+L) HRP (BS13278 bioworlde, China) and goat anti-mouse IgG (H+L) HRP (BS12478, bioworlde, China). After washing, the membranes were screened with ECL Western blotting detection reagent (NCM P10200) and visualized with the Tianneng Imaging Systems. For stripping between different antibodies, the membranes were submerged in stripping buffer (NCM WB6500) and incubated at room temperature with shaking for 10 min. The buffer was decanted, the membranes submerged in TBST washing buffer and incubated for 5 min with shaking at room temperature. The washing process was repeated 3 times. The images were analyzed using ImageJ (Subtract Background (settings: rolling ball radius, 50 px, no smoothing, sliding paraboloid) and Gels tools), ratios of phosphorylated and total protein AUCs were calculated, normalized against control sample, and plotted in GraphPad Prism 8.

## Author contributions

Anže Meden–conceptualization, methodology, formal analysis, investigation, writing–original draft, visualization; Neža Žnidaršič–methodology, formal analysis, investigation, writing–original draft; Damijan Knez–conceptualization, methodology, formal analysis, investigation, writing–original draft; Yuanyuan Wang–formal analysis, investigation, writing–original draft; Ziwei Xu–investigation; Huajing Yang–investigation; Weiting Zhang–investigation; Anja Pišlar–methodology, investigation, formal analysis, investigation, writing–original draft; Andrej Perdih–methodology, software, writing–original draft; Simona Kranjc Brezar–investigation, resources; Neža Grgurevič–methodology, formal analysis, investigation, writing–original draft; Stane Pajk–methodology, formal analysis, investigation, resources; Haopeng Sun–methodology, writing–original draft, supervision, project administration, funding acquisition; Stanislav Gobec–writing–original draft, supervision, project administration, funding acquisition. All authors have approved the final version of the article.

## Conflicts of interest

The authors declare no conflicts of interest.
