# Supplementary material for: Pleiotropic prodrugs for both symptomatic and disease-modifying treatment of Alzheimer’s disease
Source: Acta Pharm Sin B. 2025 Jul 7;15(9):4807–28. doi: 10.1016/j.apsb.2025.07.005 (PMC12491715; doi:10.1016/j.apsb.2025.07.005)

Supporting Information for

Original article

Pleiotropic prodrugs for both symptomatic and disease-modifying treatment of Alzheimer's disease

Anže Meden[a,†], Neža Žnidaršič[b,†], Damijan Knez[a], Yuanyuan Wang[c], Ziwei Xu[c], Huajing Yang[c], Weiting Zhang[c], Anja Pišlar[a], Andrej Perdih[a,d], Simona Kranjc Brezar[e], Neža Grgurevič[b], Stane Pajk[a], Haopeng Sun[c], Stanislav Gobec[a,*]

[a]*University of Ljubljana, Faculty of Pharmacy, Ljubljana SI-1000, Slovenia*

[b]*University of Ljubljana, Faculty of Veterinary Medicine, Ljubljana SI-1000, Slovenia*

[c]*China Pharmaceutical University, Nanjing 210038, China*

[d]*National Institute of Chemistry, Ljubljana SI-1000, Slovenia*

[e]*Department of Experimental Oncology, Institute of Oncology, Ljubljana SI-1000, Slovenia*

Received 2 December 2024; received in revised form 8 May 2025; accepted 6 June 2025

[*]Corresponding author.

E-mail address: stanislav.gobec@ffa.uni-lj.si (Stanislav Gobec)

[†]These authors made equal contributions to this work.

Supporting Schemes

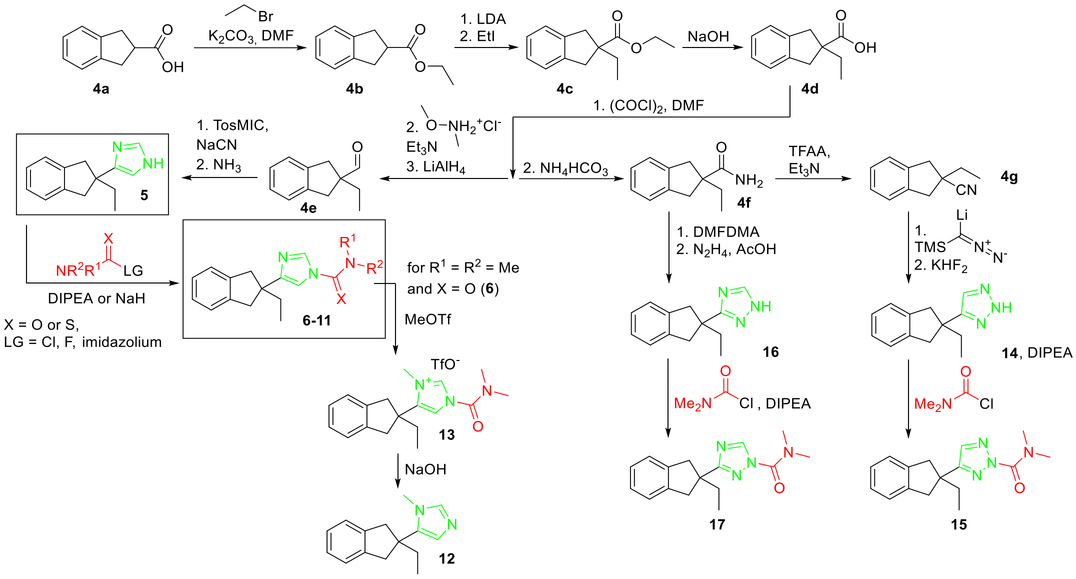

Scheme S1 The synthesis routes to compounds **5–17**. The azole parts of leaving groups are coloured green and the *N*-carbamoyl transferrable moieties in red. Commercially available indane-2-carboxylic acid **4a** was first esterified by *O*-ethylation, then the enolate of the ester **4b** was *C*-alkylated with iodoethane at low temperatures to afford **4c**. Direct diisobutylaluminum hydride (DIBAL-H) reduction of **4c** to **4e** afforded a mixture of the aldehyde **4e**, alcohol, and the starting compound **4c**, therefore another route to **4e** was sought. Since the attempts to aminolyze sterically congested **4c** with ammonia at forcing conditions or with dimethylaluminum *N*-methoxy-*N*-methylamide (generated *in situ* from trimethylaluminum and *N*,*O*-dimethylhydroxylamine hydrochloride) were unsuccessful, the ester **4c** was first hydrolysed to the carboxylic acid **4d**. The following DMF-catalysed activation with oxalyl chloride, conversion to Weinreb amide **4ea**, and the reduction of the latter to aldehyde **4e** by lithium aluminium hydride were facile. The aldehyde **4e** was then converted to atipamezole **5** in a two-step van Leusen reaction with *p*-toluenesulfonylmethyl isocyanide (TosMIC) and ammonia in acceptable yields. **5** was easily carbamoylated using different dialkyl(thio)carbamoyl chlorides and their surrogates to afford *N*-dialkylcarbamoylazoles **6–11**. Alkylation of **6** with methyl triflate afforded N^3-methylimidazolium salt **13** and, after alkaline hydrolysis, also its leaving group **12**. Primary amide **4f**, prepared from **4d** acyl chloride and ammonium bicarbonate, was dehydrated with trifluoroacetic anhydride and triethylamine to nitrile **4g**. The latter was converted with diazo(trimethylsilyl)methyllithium to trimethylsilylated 1,2,3-triazole, the TMS protection was removed by potassium bifluoride to afford 1,2,3-triazole **14**, which was

carbamoylated with dimethylcarbamoyl chloride to yield *N*-dimethylcarbamoyl-1,2,3-triazole **15**. Additionally, **4f** was converted into 1,2,4-triazole **16** through hydrazinolysis of the intermediate *N′*-acyl-*N*,*N*-dimethylformamidine, and further carbamoylated to **17**.

Supporting Tables

Table S1 The stability of the investigated compounds in an aqueous solution.

Medium	Compound	Concentration	$t_{1/2}$	k [h^{-1}]	Parent azole pK_{a1}, pK_{a2}[c]
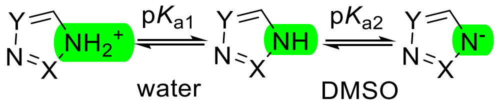					
PBS:EtOH = 80:20	**13**	10 mmol/L	13.2 h	0.052361	7.4, –
	**17**	10 mmol/L	~46 days[a]	0.000628	2.2, 14.8
	**15**	10 mmol/L	~29 days[a]	0.000982	1.2, 13.9
	**8**	10 mmol/L	stable during 48 h[b]		7.0, 18.6
	**6**	10 mmol/L	stable during 48 h[b]		7.0, 18.6
			% left after 24 h		
PBS:EtOH = 95:5	**8**	100 mg/L	100.0		
pH 2:EtOH = 95:5	**8**	100 mg/L	99.9		
pH 4:EtOH = 95:5	**8**	100 mg/L	100.0		
pH 6:EtOH = 95:5	**8**	100 mg/L	100.0		
pH 10:EtOH = 95:5	**8**	100 mg/L	97.7		

[a]Since significant decomposition did not yet occur, the calculated half-lives are only approximates.
[b]Difference between the last and the first timepoint was smaller than the experimental error.
[c]Source: Bordwell FG. Acc Chem Res 1988;21:456–63.

Table S2 The physicochemical properties of compound **8**.

Solubility[a]	[mg/L]
water	115±26
PBS	100±20
pH 2	2416±108
pH 4	166±12
pH 6	136±1
pH 10	103±17
1% Tween 20/water	615±172
pH	logD[b]
0.91	0.30±0.06
1.99	1.18±0.10
3.07	2.17±0.25
4.44	3.05±0.10
6.02	3.15±0.14
7.4	3.33±0.11
9.55	3.52±0.39
logP[c]	3.82
CLogP[c]	4.06
pK_a[d]	4.1

[a]Measured in triplicates, given as means±SD. [b]Measured as duplicates in three different volume ratios, given as means±SD. [c]Predicted by ChemDraw 23.0.1.10 (Revvity Signals Software, Inc., 2024). [d]Determined from solubility and partition measurements. The pK_a value was located at the break in the log*P* *vs.* pH and log(solubility) *vs.* pH plot.

Table S3 Cholinesterase activities in sera of C57BL/6JRccHsd mice (n = 5) undergoing repeated dosing of compound **8**. Activities were measured in triplicates (given as means±SD) and compared intra-subject before and after treatment. In some cases, not enough sera were obtained to measure activities of both ChEs.

ChE	Dosage group [mg/kg]	Activity [U/mL] **BEFORE** treatment	Activity [U/mL] **AFTER** treatment	Residual activity
**BChE**	1	0.7920±0.0795	0.2139±0.0118	27.0%±4.2%
	1	1.0059±0.0414	0.1602±0.0041	15.9%±1.1%
	1	0.7633±0.0324	0.1484±0.0071	19.4%±1.8%
	1	1.1237±0.0972	0.2261±0.0071	20.1%±2.4%
	1	1.0883±0.0552	0.2214±0.0082	20.3%±1.8%
	1		**AVERAGE:**	**20.6%±5.5%**
	5	1.0459±0.0989	0.1060±0.0071	10.1%±1.6%
	5	0.9305±0.0825	0.0989±0.0071	10.6%±1.7%
	5	1.1555±0.1049	0.0801±0.0268	6.9%±2.9%
	5	0.5654±0.0374	0.0683±0.0108	12.1%±2.7%
	5	0.8504±0.1631	0.0919±0.0000	10.8%±2.1%
	5		**AVERAGE:**	**10.1%±5.1%**
**AChE**	1	1.0459±0.1199	0.9541±0.2227	91.2%±31.8%
	1	0.9965±0.0300	1.0365±0.0653	104.0%±9.7%
	1	0.7915±0.1348	0.8481±0.0324	107.1%±22.3%
	1		**AVERAGE:**	**100.8%±40.0%**
	5	1.0318±0.1199	0.9258±0.0430	89.7%±14.6%
	5	1.1779±0.0294	0.7420±0.1450	63.0%±13.9%
	5	0.7868±0.0588	0.9517±0.0657	121.0%±17.4%
	5		**AVERAGE:**	**91.2%±26.6%**

Table S4 Characteristics of the HPLC-HRMS method used.

Analyte–matrix	**5**–human plasma		**8**–human plasma	
Range [µg/L]	0.01–30		0.01–30	
Method	Internal standard		Internal standard	
k	0.020896		0.017863	
n	–0.004022		0.000624	
R^2	0.997837		0.997662	
Concentration [µg/L]	0.3	3	0.3	3
Accuracy	165%	86%	157%	92%
Concentration [µg/L]	1	10	1	10
Precision (RSD)	0.86%	0.67%	3.45%	3.27%
Analyte–matrix	**5**–mice plasma		**8**–mice plasma	
Range [µg/L]	0.05–50		0.01–10	
Method	Internal standard		Internal standard	
k	0.0225021		0.036856	
n	0.031638895		0.001156	
R^2	0.999975		0.999692	
Concentration [µg/L]	0.5	3	0.2	2
Accuracy	99.1%	103.4%	88.3%	104.1%
Concentration [µg/L]	1	10	0.1	10
Precision (RSD)	1.78%	1.36%	2.93%	1.70%
Analyte–matrix	**5**–mice brain		**8**–mice brain	
Range [µg/L]	0.1–50		0.1–100	
Method	Internal standard		Internal standard	
k	0.123685925		0.145103506	
n	–0.04452303		0.140266161	
R^2	0.996690		0.995988	
Concentration [µg/L]	2	20	2	20
Accuracy	94.1%	136.9%	117.8%	123.7%
Concentration [µg/L]	1	10	1	10
Precision (RSD)	3.95%	2.08%	1.68%	2.11%

Table S5 α_{2A}(*h*) Binding assay data.

Compound	Concentration [mol/L]	Measurement	Value
**5**	1.0e-09	% Inhibition	38.2155
**5**	1.0e-08	% Inhibition	82.9921
**5**	1.0e-07	% Inhibition	97.5191
**5**	1.0e-06	% Inhibition	100.628
**5**	3.0e-06	% Inhibition	99.9701
**5**		Bottom	0
**5**		IC_{50} [mol/L]	1.73e-09
**5**		K_i [mol/L]	7.70e-10
**5**		nH	0.88681
**5**		Top	100.445
**6**	1.0e-08	% Inhibition	9.042
**6**	1.0e-07	% Inhibition	–6.56e+00
**6**	1.0e-06	% Inhibition	5.99313
**6**	3.0e-06	% Inhibition	19.8625
**6**	1.0e-05	% Inhibition	44.9709
**12**	1.0e-09	% Inhibition	–4.04e-01
**12**	1.0e-08	% Inhibition	4.67793
**12**	1.0e-07	% Inhibition	–3.93e+00
**12**	1.0e-06	% Inhibition	14.8408
**12**	3.0e-06	% Inhibition	20.3408
**14**	1.0e-08	% Inhibition	3.66164
**14**	1.0e-07	% Inhibition	–5.23e-01
**14**	1.0E-06	% Inhibition	6.05291
**14**	3.0e-06	% Inhibition	2.34644
**14**	1.0e-05	% Inhibition	8.50396
**16**	1.0e-08	% Inhibition	4.37902
**16**	1.0e-07	% Inhibition	–4.47e+00
**16**	1.0e-06	% Inhibition	2.10731
**16**	3.0e-06	% Inhibition	–9.25e+00
**16**	1.0e-05	% Inhibition	2.70513
Yohimbine		Concurrent IC_{50}	4.87e-09
Yohimbine		Concurrent K_i	2.17e-09
Yohimbine		nH	1.14856

Supporting Figures

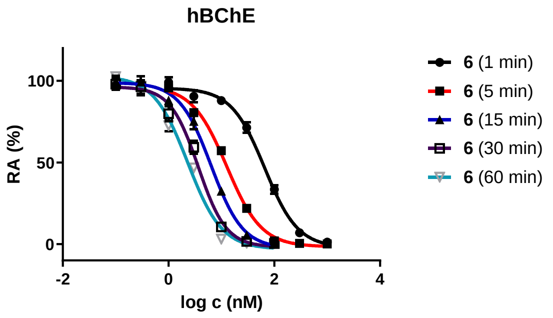

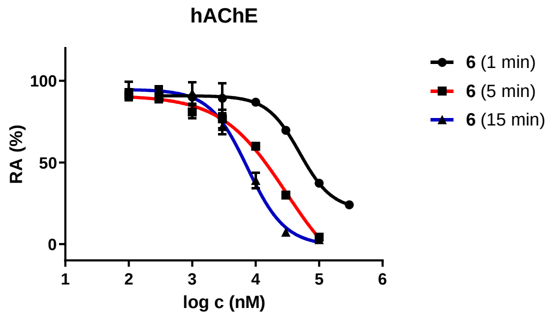

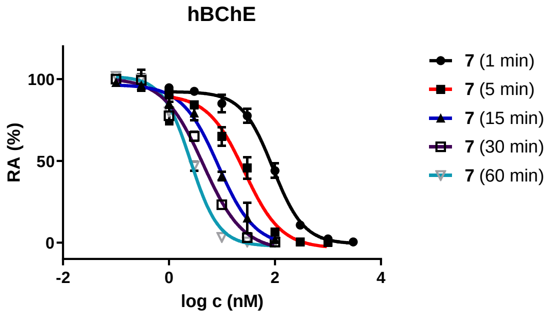

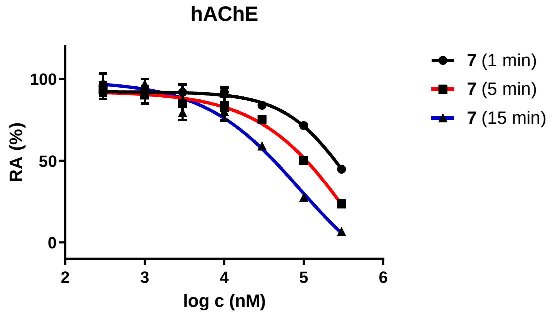

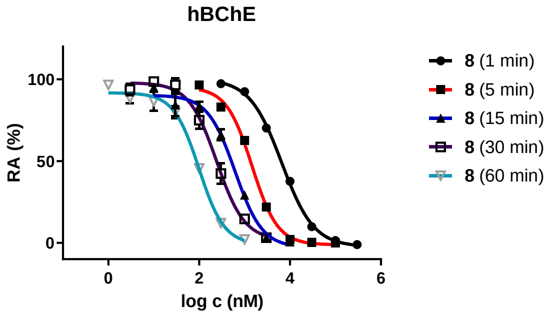

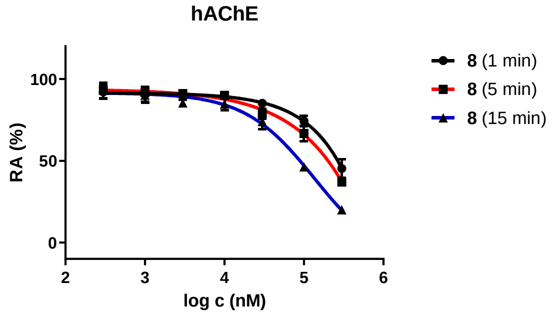

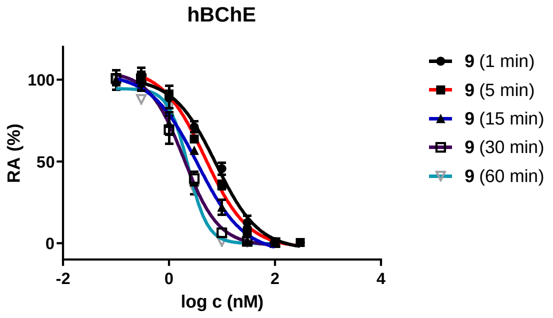

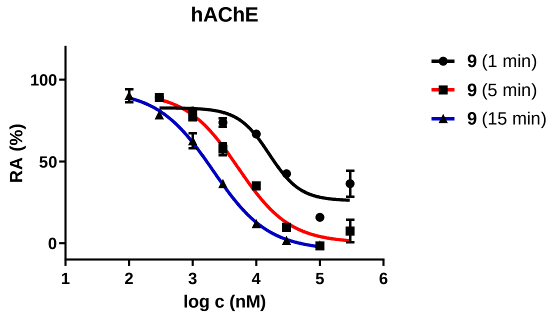

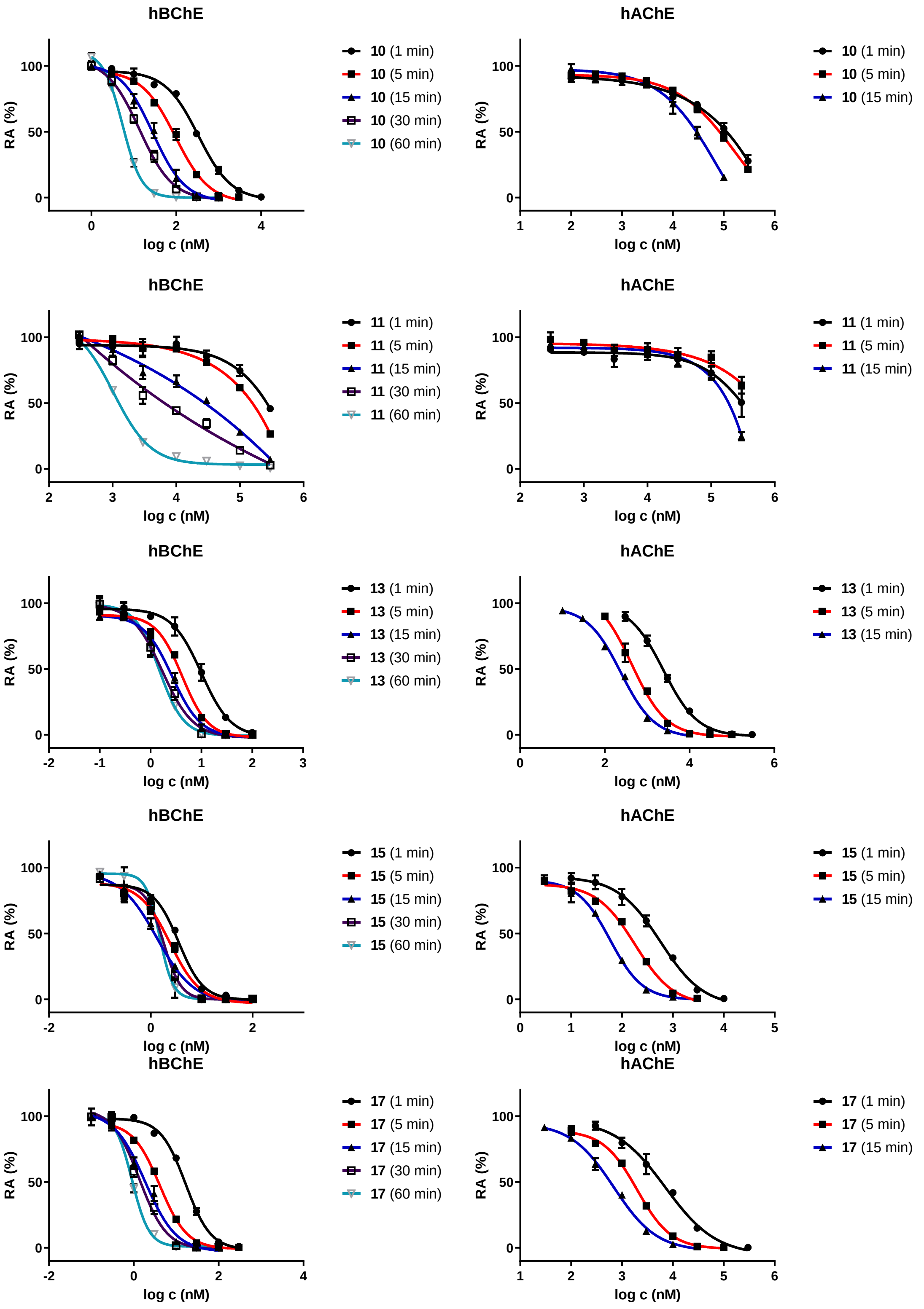

Figures S1–S2 Time-dependency experiments on hBChE and hAChE for *N*-carbamoylazoles **6**–**11**, **13**, **15**, and **17**. The residual activities are plotted against log-concentration. The leftward shift, *i.e.*, the decreasing IC_{50} values with longer preincubation times indicates the covalent mechanism of inhibition.

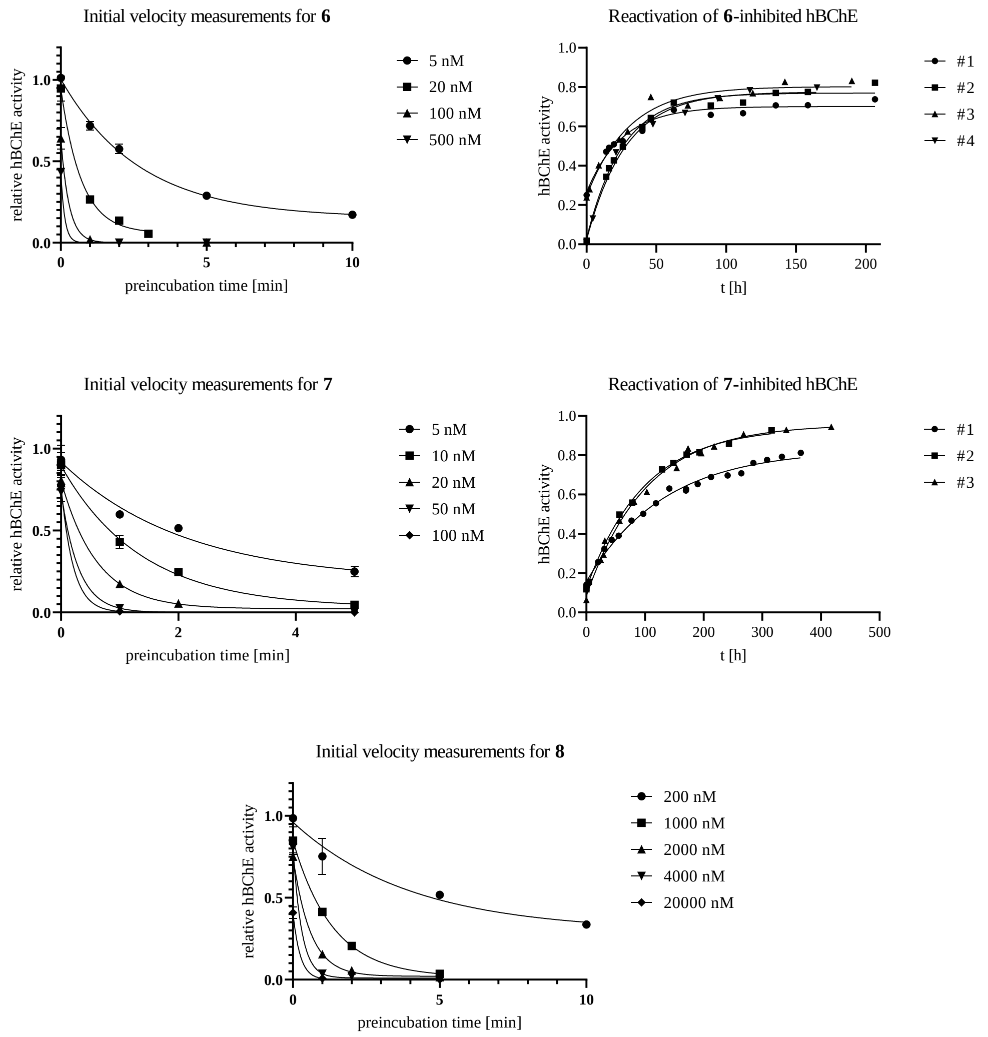

Figure S3 Plots for determination of kinetic parameters for hBChE inhibition and reactivation at 25 °C, obtained by initial velocity measurements, values are given as means±SEM (if available).

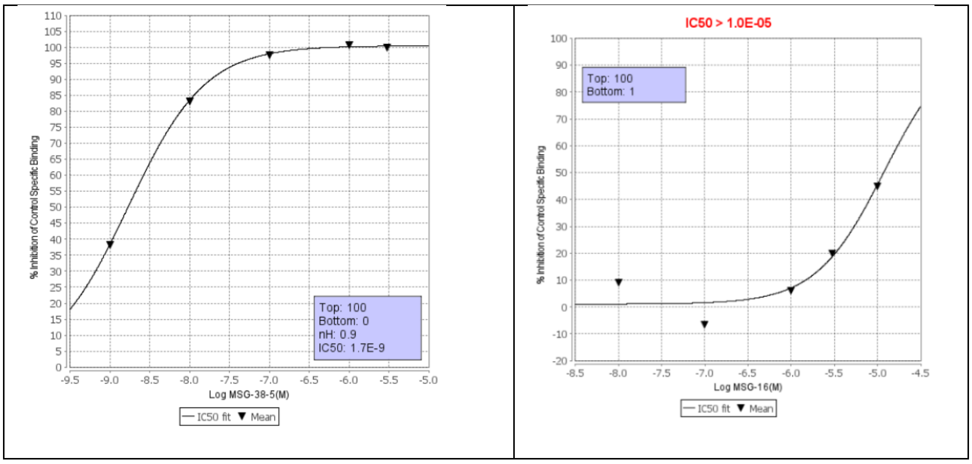

Figure S4 α_{2A} binding assay – concentration-response curve for **5** (left) and **6** (right).

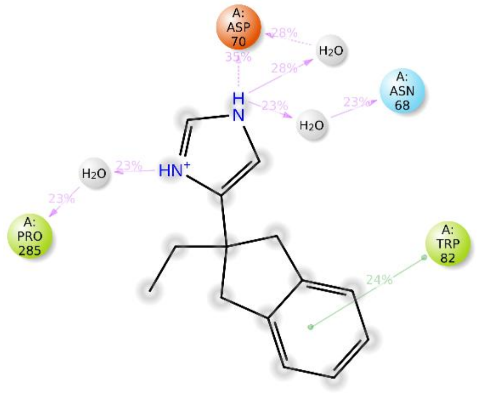

A

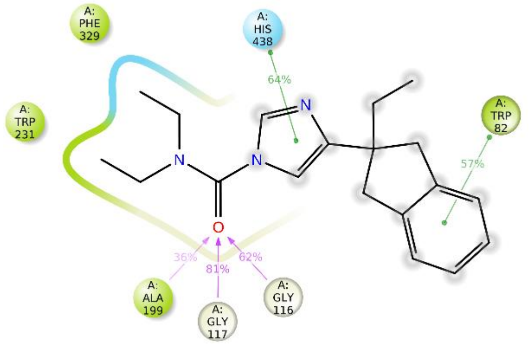

B

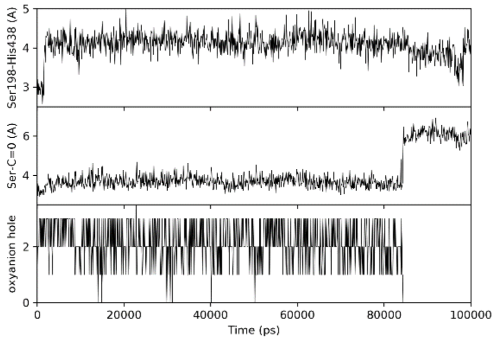

C

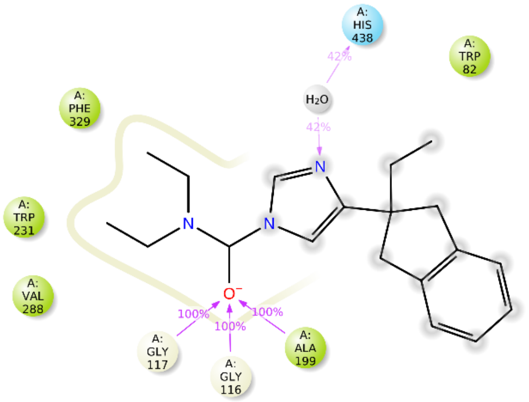

D

Figures S5 (A) Ligand interaction diagram based on 100 ns MD simulation of compound **5** in hBChE active site. Protein-ligand contacts and interactions that occur for more than 20% of the MD simulation time are shown: the *π*–*π* interactions as green lines, the cation–*π* interactions as red lines, hydrogen bonds are shown in blue and ionic interactions in magenta. Grey circles denote solvent exposure. (B, C) 100 ns MD simulation of **8** in hBChE: (B) Ligand interaction diagram; (C) Top, time-dependence plot of interatomic distance between Ser198's *Oγ* and His438's *Nε* (in Å, indicating the stability of the catalytic triad). Middle, the time-dependence plot of interatomic distance between Ser198's *Oγ* and **8**'s carbonyl carbon (in Å, indicating the propensity for covalent bond formation). Bottom, the number of hydrogen bonds formed with the oxyanion hole residues (Gly116, Gly117, Ala199). (D) Ligand interaction diagram based on 100 ns MD simulation of **8**'s tetrahedral intermediate (post-reaction pose) in the hBChE active site.

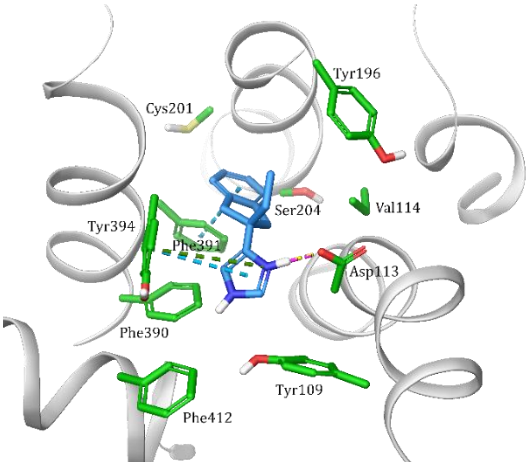

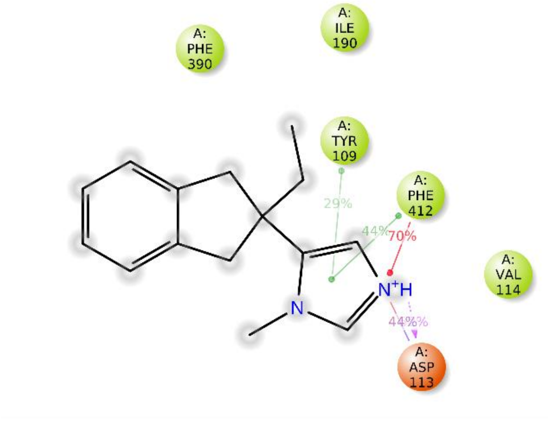

5

12

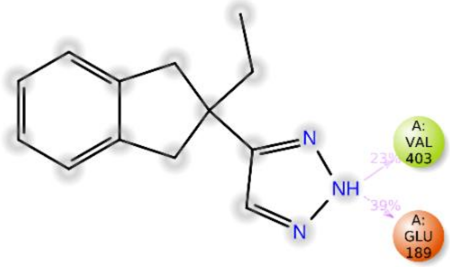

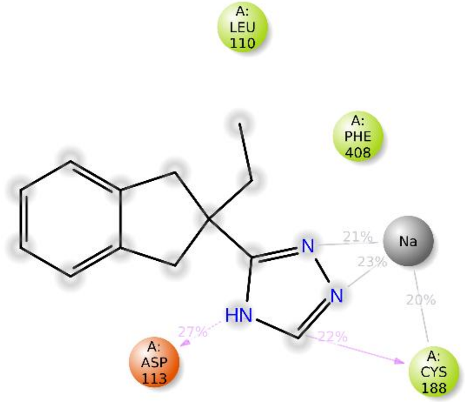

14

16

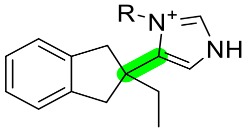

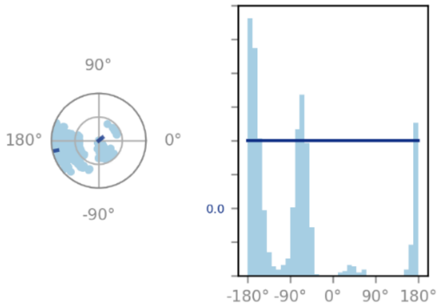

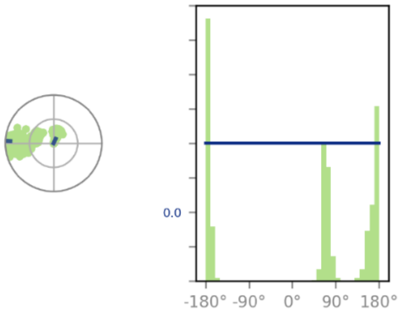

5

12

Figure S6 Top left – the 500th frame of the 1 μs all-atom MD of **5** in α_{2A} adrenoreceptor (view from the top, orthogonally to the membrane). The key amino acid residues are shown as green sticks and **5** as azure sticks. The hydrogen bonds are shown as yellow dashed lines, π–π interactions as blue dashed lines, cation–π interactions as dark green

dashed lines, and salt bridge with magenta dashed line. This pose, locked in the aromatic cage, remained fairly constant during the second part of the simulation. Continued – ligand interaction diagrams for compounds **12**, **14**, and **16** (100 ns MD) in the α_{2A} adrenoreceptor binding site. Bottom, comparison of available torsional space for the highlighted bond in **5** and **12**. Radial plots describe the conformation of the torsion throughout the course of the simulation, from centre radially outwards. The bar plots show probability density of the specific torsion with torsion potential [kcal/mol] plotted *vs.* torsional angle. Rotation of **12** *vs.* **5** is slightly hindered.

Computational studies—commentary

N-Carbamoylazoles produced highly comparable docking poses in the hBChE active site for both pre-reaction and tetrahedral intermediate states. More precisely, the carbonyl oxygen of these compounds was tridentately coordinated within the oxyanion hole comprised of Gly116, Gly117, and Ala199, while the *N*-alkyls were oriented towards Trp231 and Leu286 residues in the acyl-binding pocket, the azole moiety formed a *π–π* interaction with His438, and the indan-2-yl moiety with Trp82 from choline-binding pocket. Meanwhile, the leaving groups mainly occupied the choline-binding pocket and exhibited *π–π* interactions of the azole and phenyl moieties with both Trp82 and Tyr332, and/or His438 residues.

The persistence of these interactions as well as the generated binding modes were further examined by molecular dynamics (MD) simulations (Fig. S5A–S5D). During 100 ns of simulation, **5** primarily interacted with Trp82 through van der Waals interactions and transient *π–π* interactions. Additionally, hydrogen bonding with Asp70, which occurred directly or *via* water molecules, was detected (Fig. S5A). Meanwhile, during first 85 ns of MD simulation, compound **8** consistently formed at least two hydrogen bonds with the residues of oxyanion hole, its carbamoyl carbon was placed at the proper distance from the Ser198's *Oγ* for the reaction to occur (approx. 3.5 Å), and both aromatic rings were involved in *π–π* interactions (Fig. S5B and S5C). In addition, the postreaction tetrahedral intermediate of **8** was fixed in the oxyanion hole, while the rest of the molecule interacted mainly *via* van der Waals hydrophobic contacts during the simulated 100 ns (Fig. S5D). This intermediate disintegrates into Ser198–*N*,*N*-diethylcarbamoylated hBChE and leaving group **5**, which stayed in the enzyme

active site during the 2000 ns MD simulation, forming transient π–π interactions with nearby aromatic residues and a reoccurring hydrogen bond with Trp82 (not shown).

As only reversible non-covalent binding is expected of these compounds within α_{2A} adrenoreceptors, we performed only regular docking experiments. Although the ionic interaction of the protonated **5** with Asp113 was identified, the used scoring function failed to recognize the superior binding of **5** *vs.* carbamoylazoles. The limitations of scoring functions to properly rank docking solutions are well known, therefore this was somewhat inherently expected.

Hence, for a more meaningful insight into the interaction profile of these compounds with α_{2A} adrenoreceptors, a series of molecular dynamics (MD) simulations was carried out for compounds **5**, **12**, **14**, **16** (Fig. S6). A 1 μs all-atom MD simulation of compound **5** in α_{2A} adrenoreceptor reached equilibrium after approximately 220 ns and revealed a stable pose with a persistent hydrogen bond and ionic interaction between atipamezole's protonated imidazole and Asp113$^{3.32}$ residue, which is a necessary recognition motif in adrenoreceptors (actually, in all monoaminergic and opioid receptors). The rest of the molecule formed recurrent van der Waals contacts with Phe390, Phe391, Tyr394, and Ile190 sidechains, and recurrent water bridges with Tyr394 and Phe411. Surprisingly, compound **12** was able to productively engage Asp113$^{3.32}$, either *via* an ionic interaction or, after the binding pose changed at the middle of the simulation, a hydrogen bond, and van der Waals contacts with aromatic cage phenylalanines and tyrosines. However, its binding pose was different from that of atipamezole **5**.

On the other hand, after only a few simulated frames, compound **14** dissociated from the binding site, and remained in its vicinity, where it interacted with residues lining the entrance to the binding site (namely, hydrogen bonds with Glu189 and Val403, and several van der Waals contacts). After initial hydrogen bonding to Asp113, compound **16** also moved towards the entrance of the binding site but did not fully dissociate during the simulated 100 ns. Similarly, the prodrug **6** also did not exhibit a stable binding pose but rather remained positioned near the entrance to the binding site. These computational results are in accordance with the observed lack of binding of these compounds in the displacement assay.

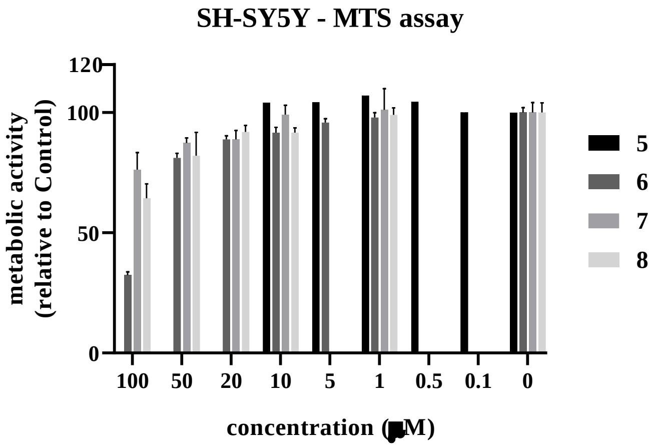

Figure S7 *In vitro* metabolic activity of SH-SY5Y cell treated with compounds **5**–**8**. The control group (DMSO) was considered as 100% cell viability. Data are means±SEM of three independent experiments, each carried out in quadruplicate.

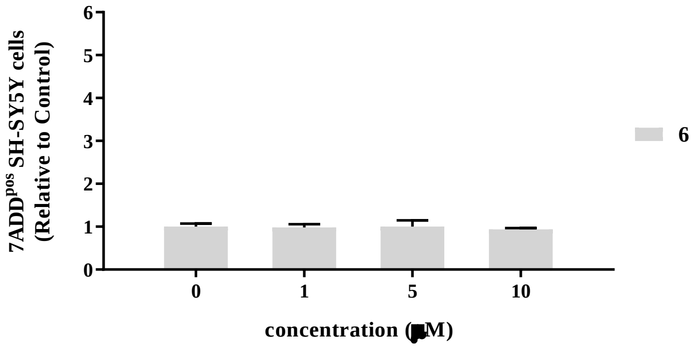

Figure S8 *In vitro* cytotoxicity profile of **6** on SH-SY5Y cells. Data are relative percentages of 7-AAD-positive (7-AAD^{pos}) cells normalized to the control (DMSO). Data are means±SEM of at least two independent experiments, each performed in duplicate.

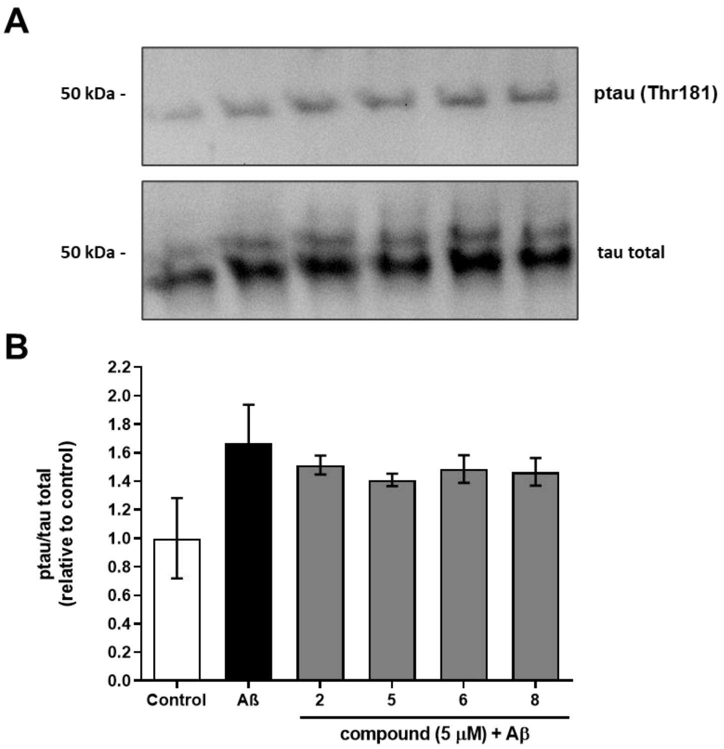

Figure S9 The effects of compounds on Aβ-induced Tau signalling in SH-SY5Y cells. Representative Western blots (A) and quantification (B) of relative values of the phosphorylated form of tau (Thr181) compared to the total tau form after pre-aggregated A$\beta_{1\text{–}42}$ (5 µmol/L) treatment in the absence or presence of compounds at concentration of 5 µmol/L.

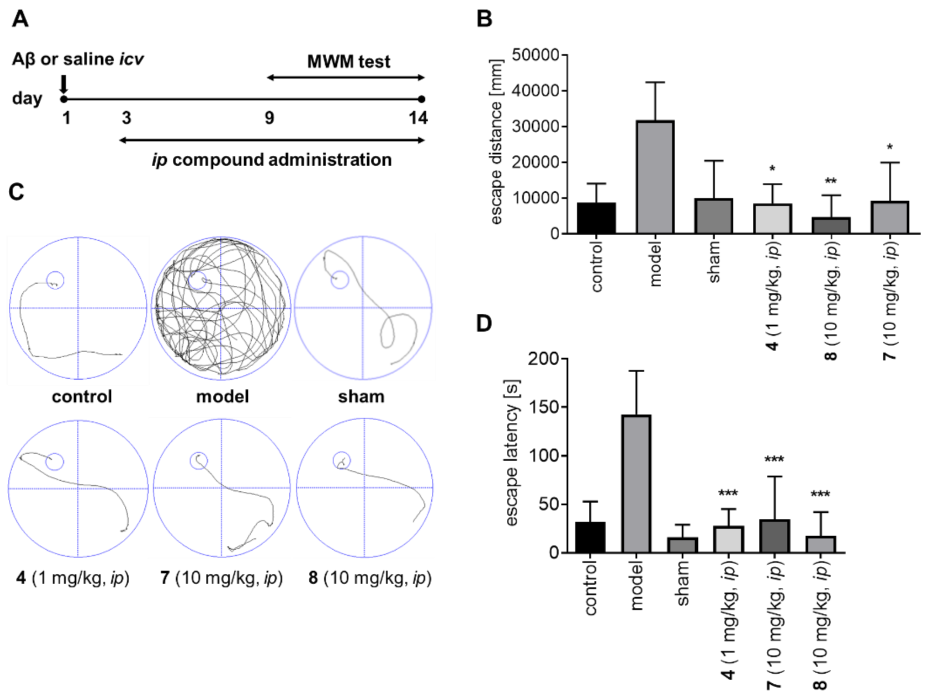

Figure S10 Effects of treatment with compounds **7**, **8** and rivastigmine **4** on cognitive impairment induced by intracerebral injection of A$\beta_{1\text{–}42}$ in ICR mice. (A) A schematic diagram of experimental design. Mice were intracerebroventricularly (icv) injected with saline or A$\beta_{1\text{–}42}$ peptide (10 μg) on Day 1. Compounds were then administrated intraperitoneally (*ip*) once a day for 12 consecutive days after surgery, Morris water maze (MWM) task was conducted during Days 9–14, and tissues were harvested on Day 14. (B) The swum distance to the platform in MWM task. Values are expressed as means±SD (n = 4; $^{**}P < 0.01$, $^{*}P < 0.05$ *vs.* A$\beta_{1\text{–}42}$ model group. (C) Representative trajectories of mice to the platform in MWM test. (D) The mice' latency for the first escape to the platform in MWM task. Values are expressed as means±SD (n = 4; $^{***}P \leq 0.001$ *vs.* A$\beta_{1\text{–}42}$ model group).

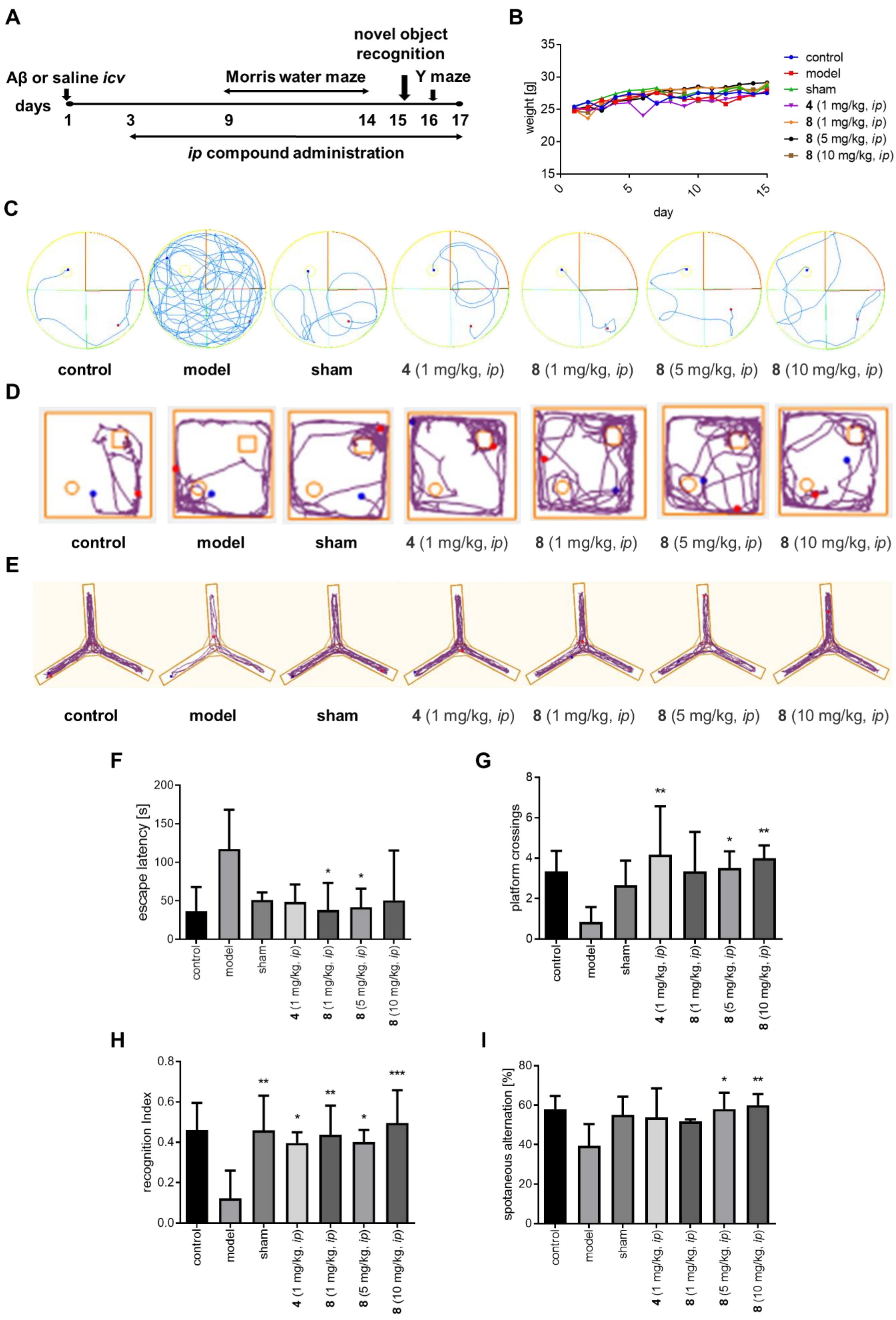

Figure S11 Effects of ip treatment with compound **8** and rivastigmine **4** on cognitive impairment in ICR mice, induced by intracerebral injection of A$\beta_{1\text{–}42}$. (A) A schematic diagram of experimental design. Mice were icv injected with either saline or A$\beta_{1\text{–}42}$

peptide (10 μg) on Day 1. Compounds were then administrated once a day for 12 consecutive days after surgery, Morris water maze task was conducted during Days 9–14, novel object recognition test was conducted on Day 15, Y maze test was conducted on Day 16, and the tissues were harvested on Day 17. (B) Mice body weight changes during the experiment. (C) Representative mice trajectories to the platform in MWM test. (D) Representative mice trajectories in NOR test. (E) Representative trajectory of mice to explore the novel arm in Y maze test. (F) The mice' latency for the first escape to the platform in MWM test. (G) The number of platform crossings in MWM test. (H) The recognition index in NOR test. (I) The spontaneous alternation in Y maze test. Values are given as means±SD ($n = 6$; $^{***}P < 0.001$, $^{**}P < 0.01$, $^{**}P < 0.01$, $^{*}P < 0.05$ *vs.* model group).

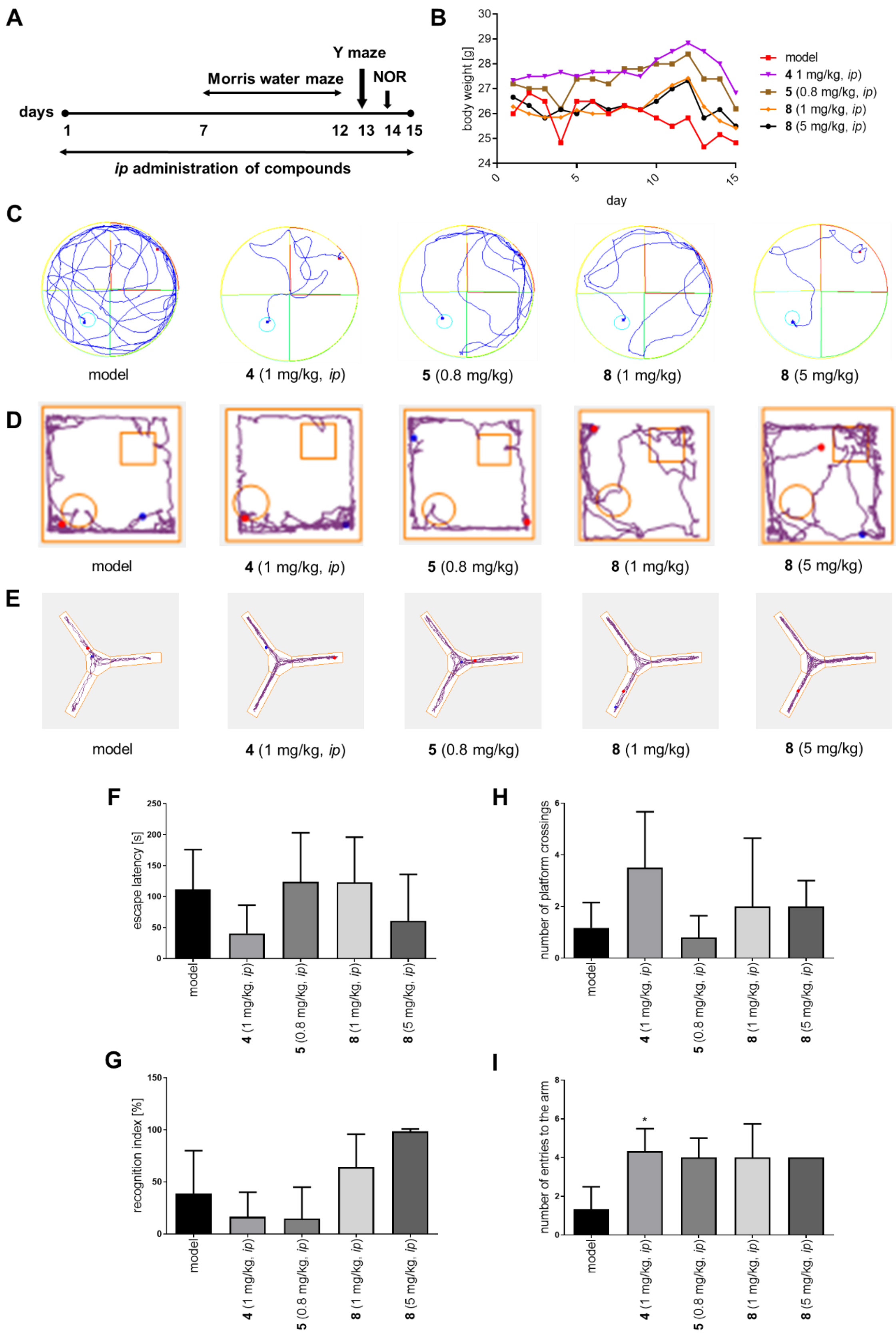

Figure S12 Effects of ip treatment with compound **8,** rivastigmine **4** and atipamezole **5** hydrochloride on cognitive impairment in APP/PS1 mice model. (A) A schematic diagram of experimental design. Compounds were administrated ip once a day for 15

consecutive days. Morris water maze task was conducted during Days 7–12, novel object recognition test was conducted on Day 14, Y maze test was conducted on Day 15, and the tissues were harvested on Day 15. (B) Mice body weight changes during the esperiment. (C) The representative mice trajectories to the platform in MWM test. (D) The representative mice trajectories in NOR test. (E) The representative trajectories of mice to explore the novel arm in Y maze test. (F) The mice' latency for the first escape to the platform location in MWM test. (G) The recognition index in NOR test. (H) The number of platform crossings in MWM test. (I) The spontaneous alteration in Y maze test. Values are given as means±SD (***$P < 0.001$, **$P < 0.01$, **$P < 0.01$, *$P < 0.05$ *vs.* model group).

Raw Western blot images for Figure 4

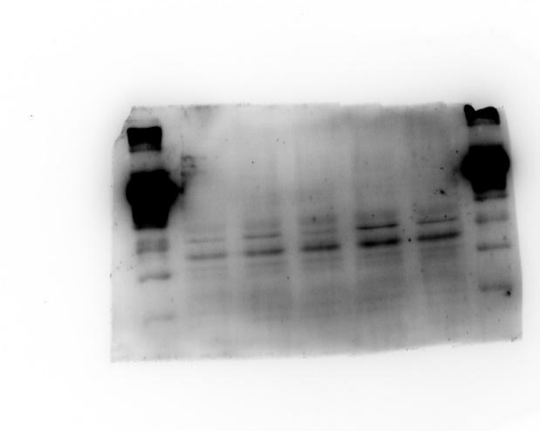

blot #1 – pGSK3*β*

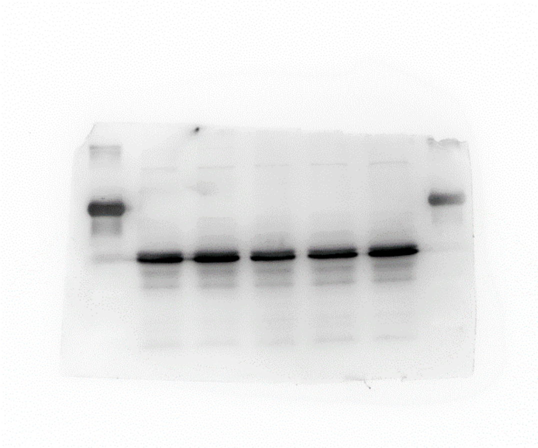

blot #1 – GSK3*β*

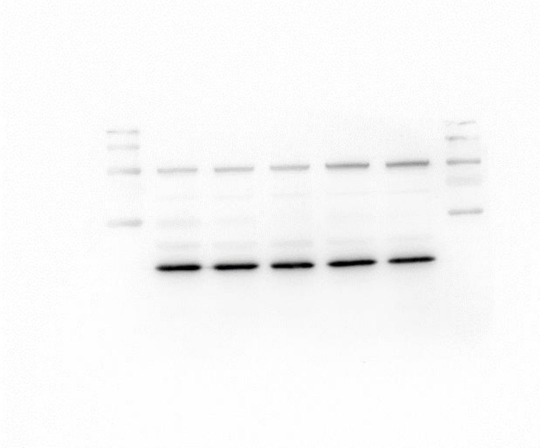

blot #1 – GAPDH

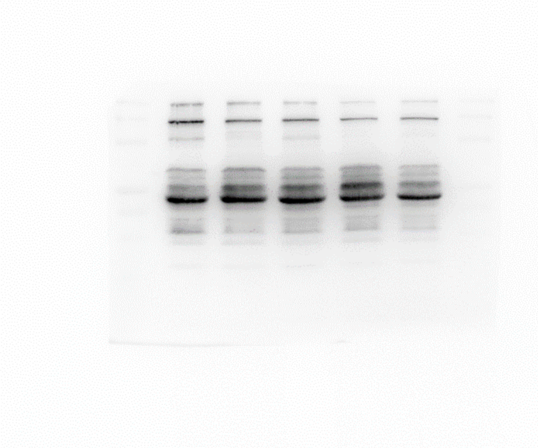

blot #2 – ptau

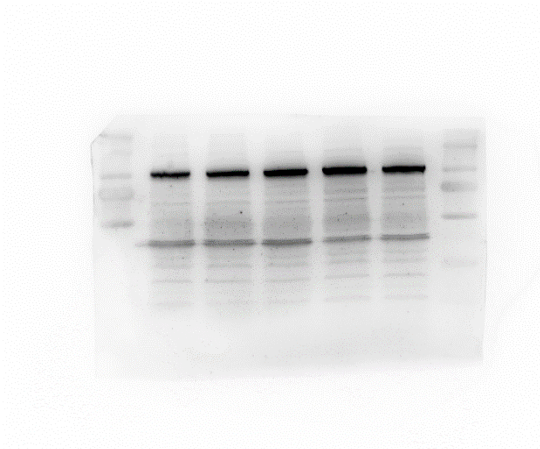

blot #2 – tau

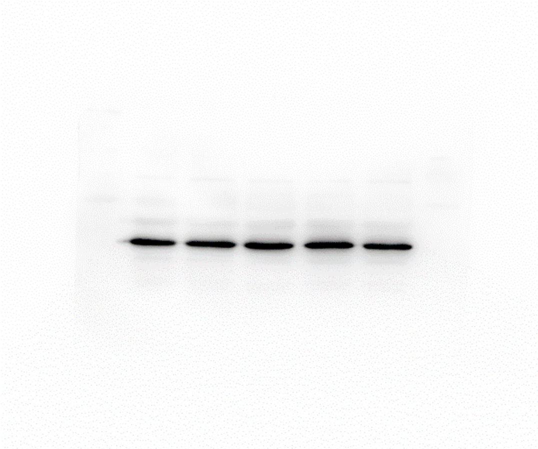

blot #2 – GAPDH

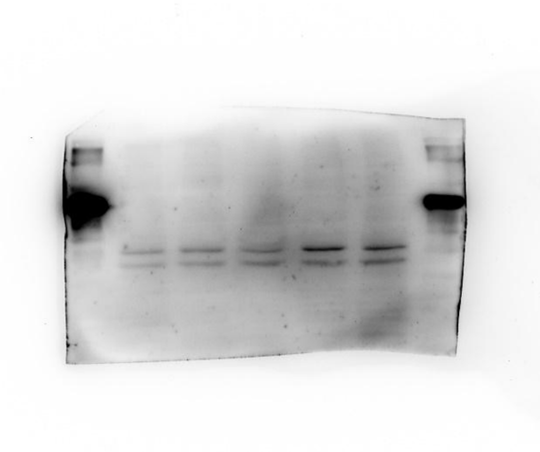

blot #3 – pGSK3β

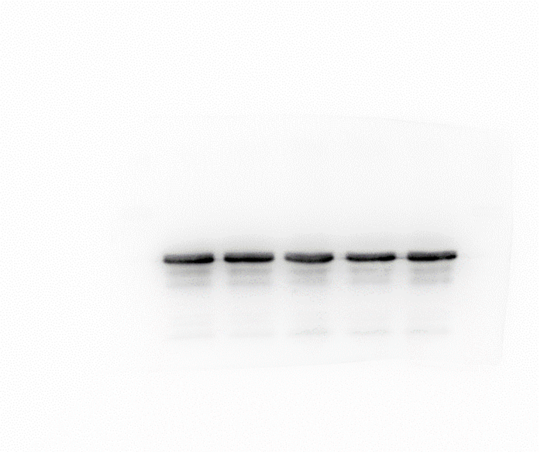

blot #3 – GSK3β

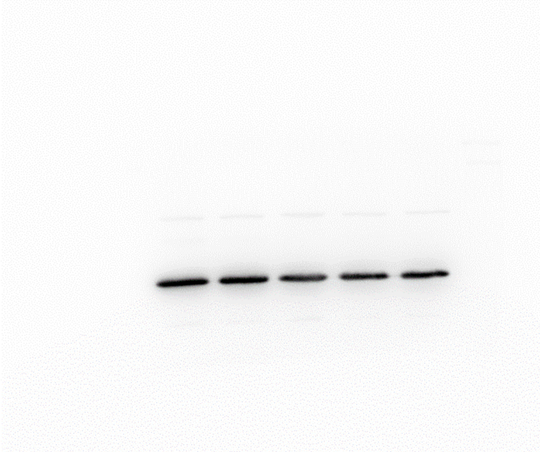

blot #3 – GAPDH

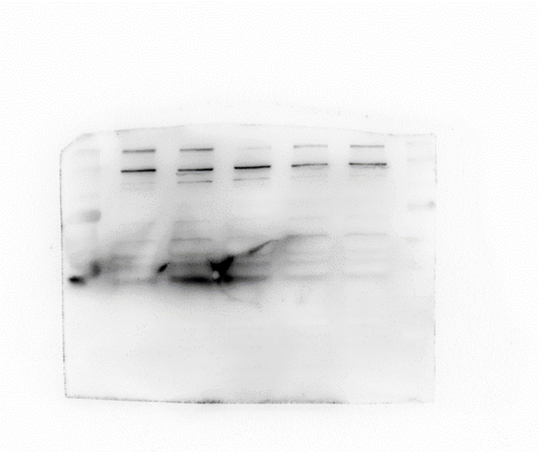

blot #4 – ptau

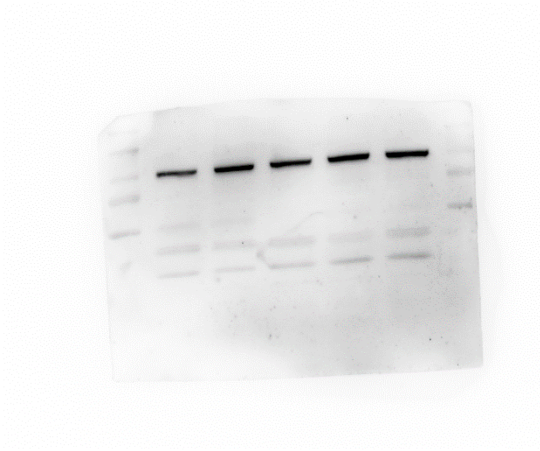

blot #4 – tau

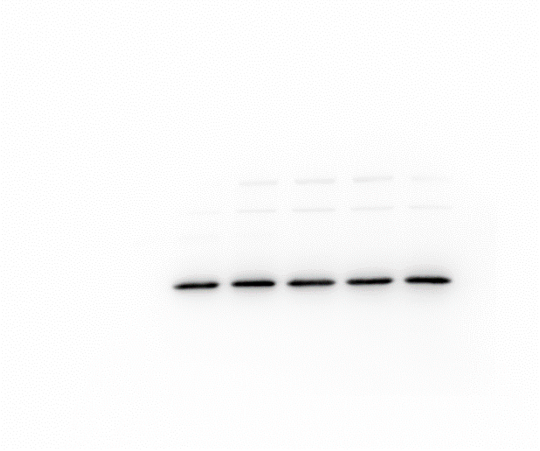

blot #4 – GAPDH

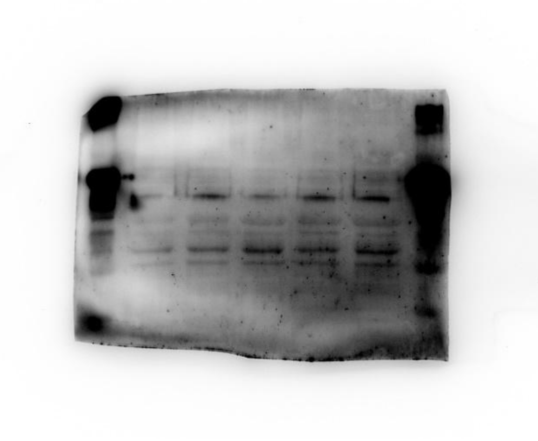

blot #5 – pGSK3β

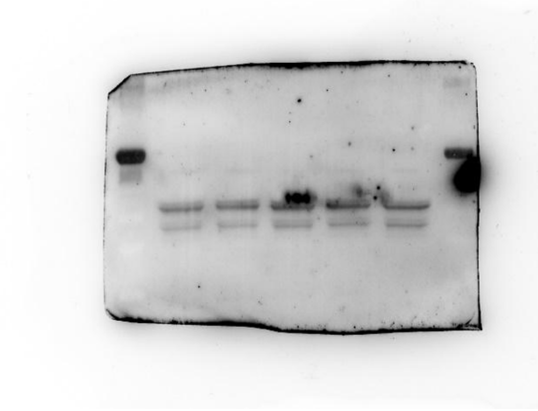

blot #5 – GSK3β

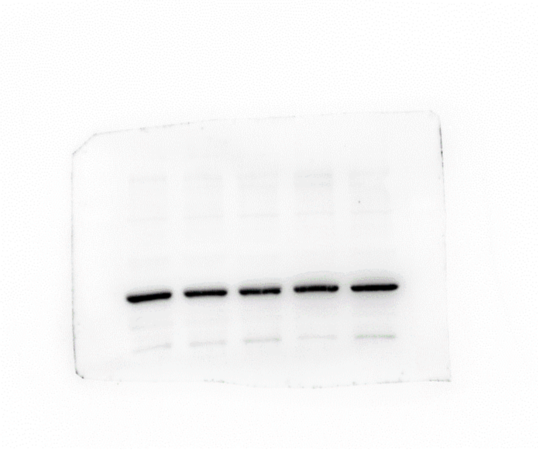

blot #5 – GAPDH

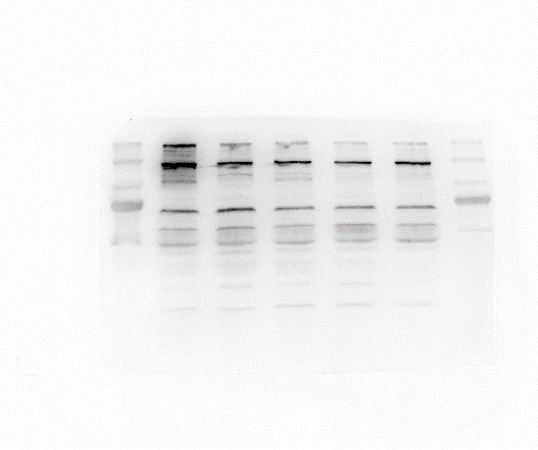

blot #6 – ptau

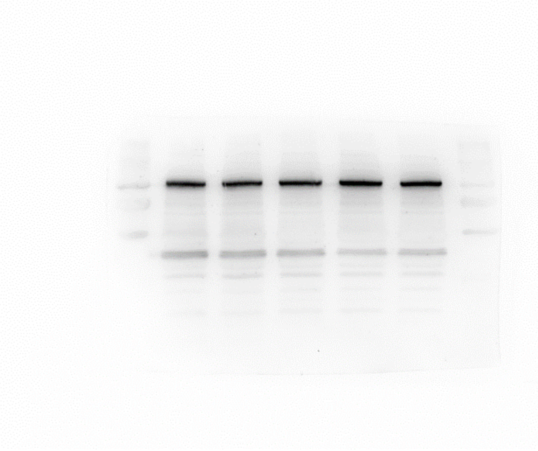

blot #6 – tau

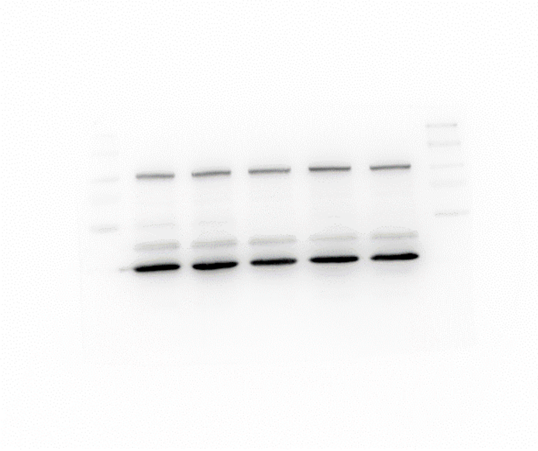

blot #6 – GAPDH

NMR spectra

4b

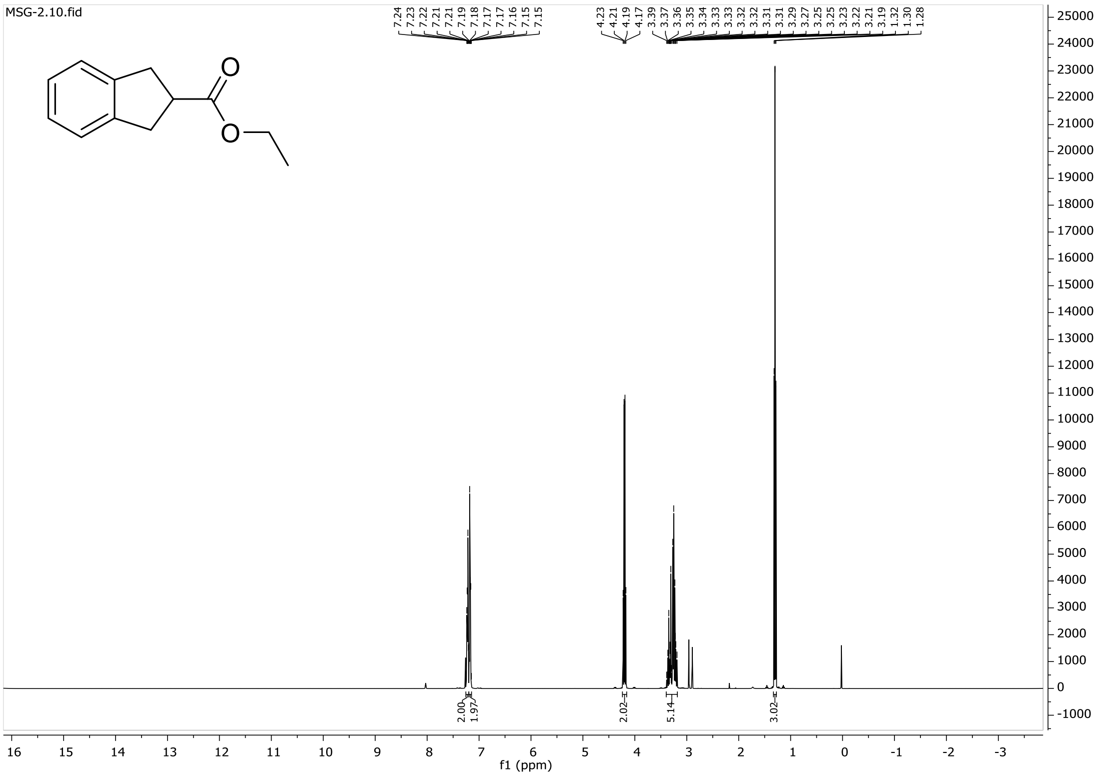

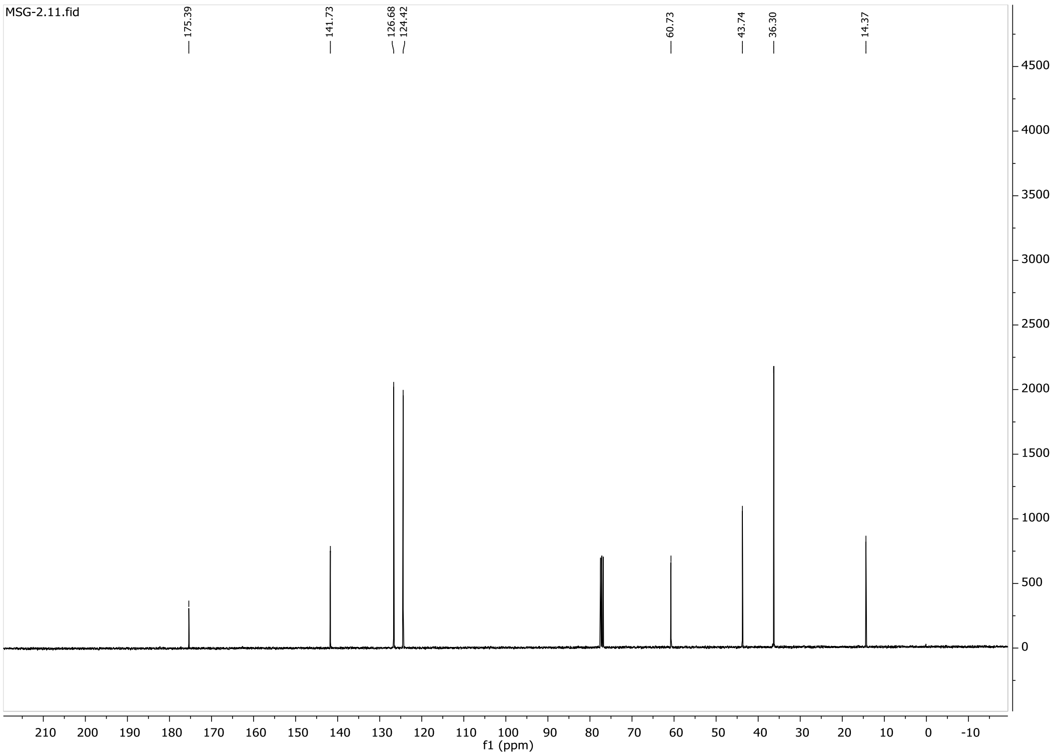

4c

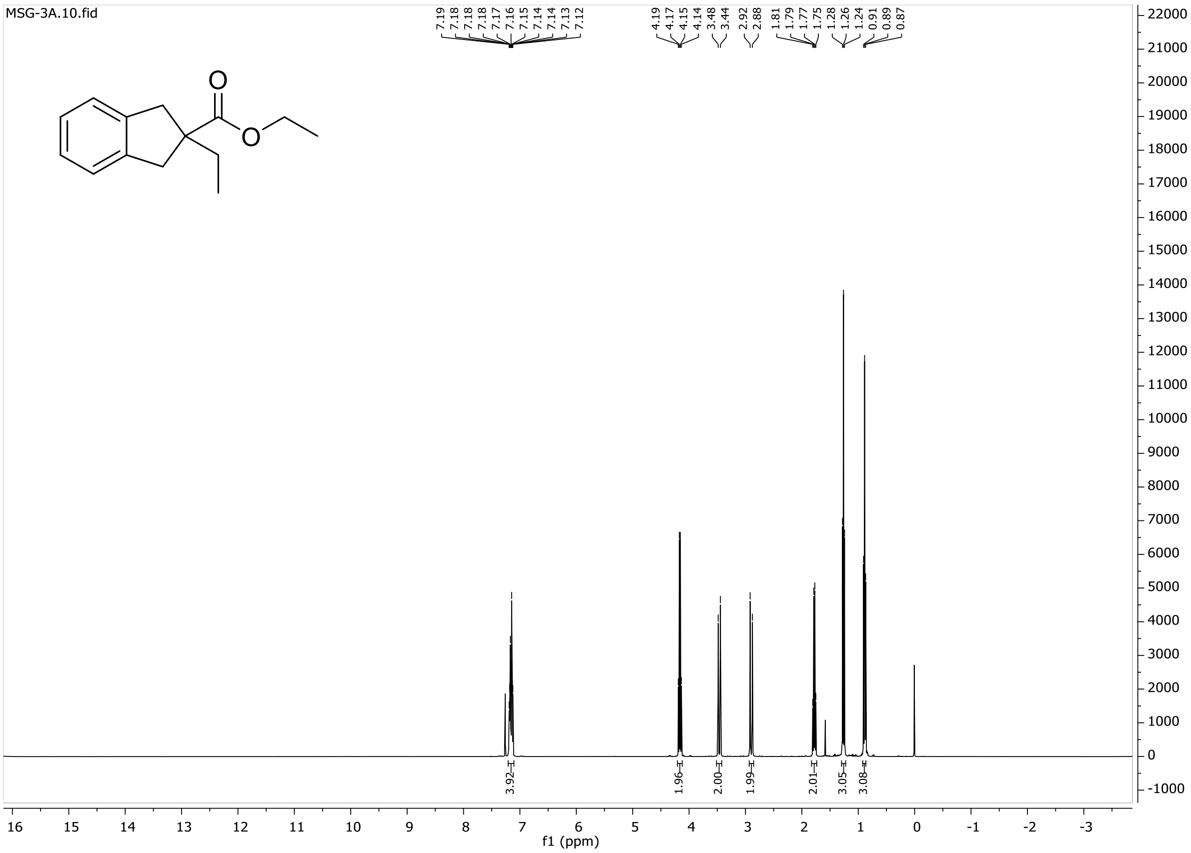
MSG-3A.10.fid
7.19
7.18
7.18
7.18
7.17
7.16
7.15
7.14
7.14
7.13
7.12
4.19
4.17
4.15
4.14
3.48
3.44
2.92
2.88
1.81
1.79
1.77
1.75
1.28
1.26
1.24
0.91
0.89
0.87
3.92
1.96
2.00
1.99
2.01
3.05
3.08
f1 (ppm)

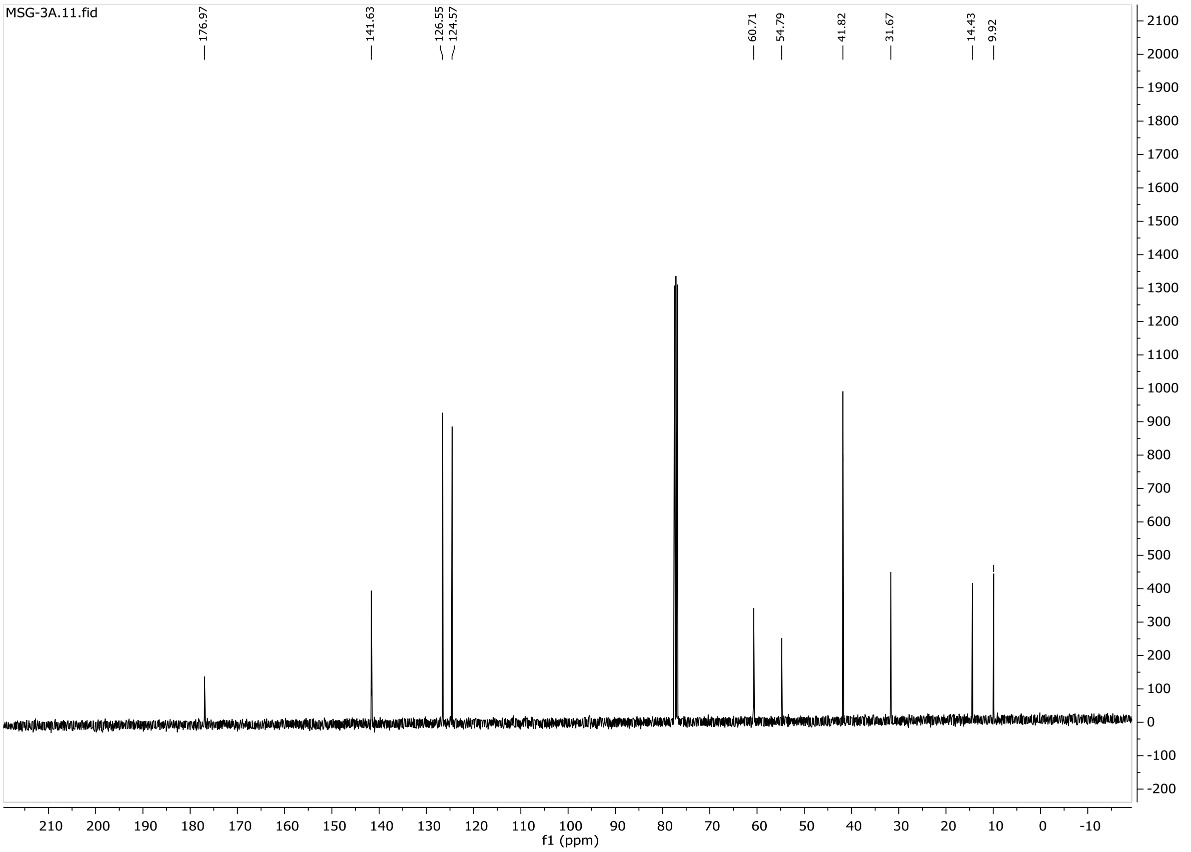
MSG-3A.11.fid
176.97
141.63
126.55
124.57
60.71
54.79
41.82
31.67
14.43
9.92
f1 (ppm)

4d

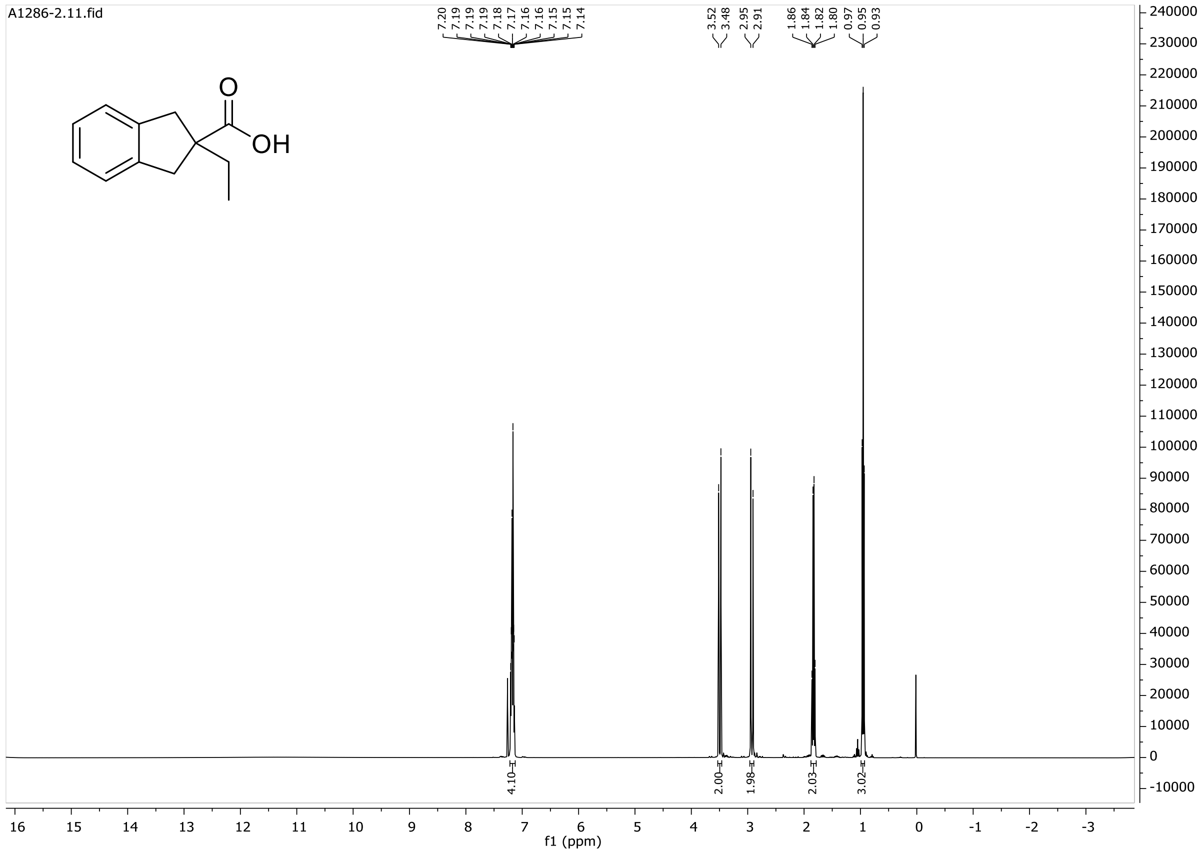
A1286-2.11.fid
7.20
7.19
7.19
7.19
7.18
7.17
7.16
7.16
7.15
7.15
7.14
3.52
3.48
2.95
2.91
1.86
1.84
1.82
1.80
0.97
0.95
0.93
4.10
2.00
1.98
2.03
3.02
f1 (ppm)

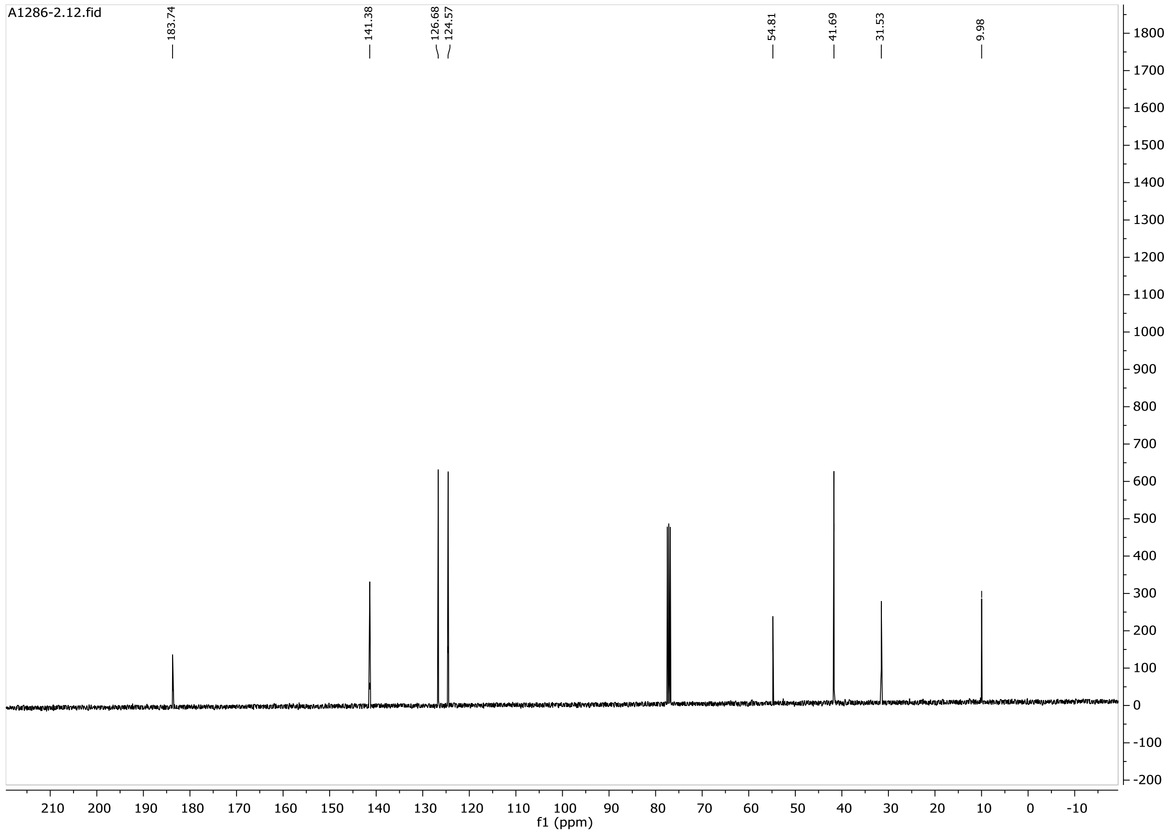
A1286-2.12.fid
183.74
141.38
126.68
124.57
54.81
41.69
31.53
9.98
f1 (ppm)

4ea

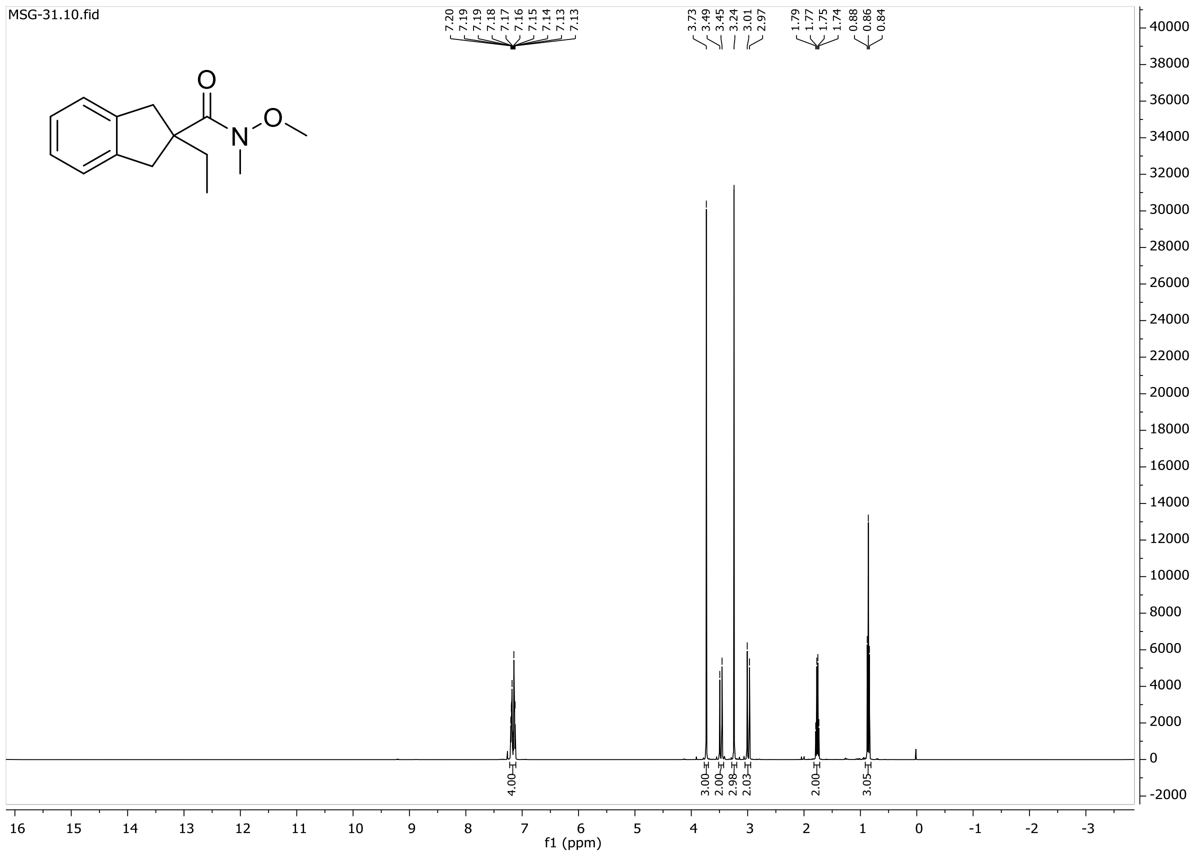
MSG-31.10.fid
7.20 7.19 7.19 7.18 7.17 7.16 7.15 7.14 7.13 7.13
3.73 3.49 3.45 3.24 3.01 2.97
1.79 1.77 1.75 1.74 0.88 0.86 0.84
O
N
O
4.00
3.00 2.00 2.98 2.03
2.00
3.05
f1 (ppm)

4e

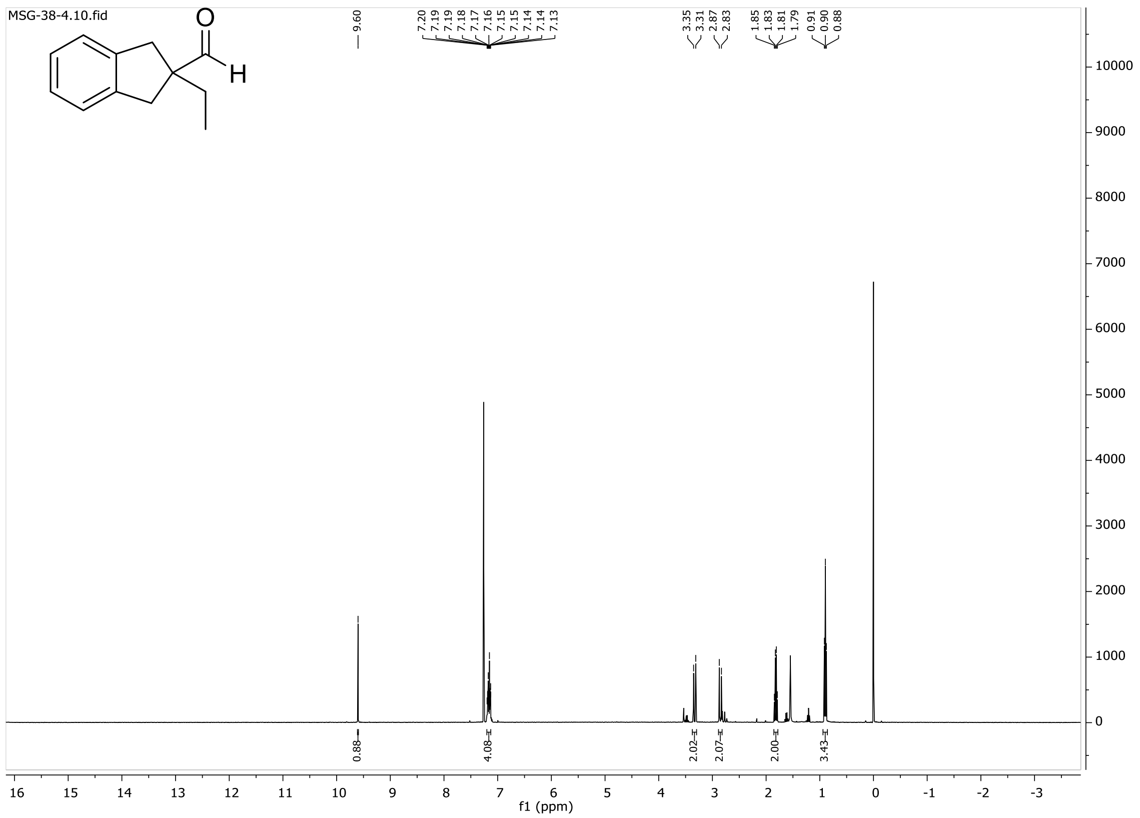
MSG-38-4.10.fid
9.60
7.20 7.19 7.19 7.18 7.17 7.16 7.15 7.15 7.14 7.13
3.35 3.31 2.87 2.83
1.85 1.83 1.81 1.79 0.91 0.90 0.88
O
H
0.88
4.08
2.02 2.07
2.00
3.43
f1 (ppm)

4g

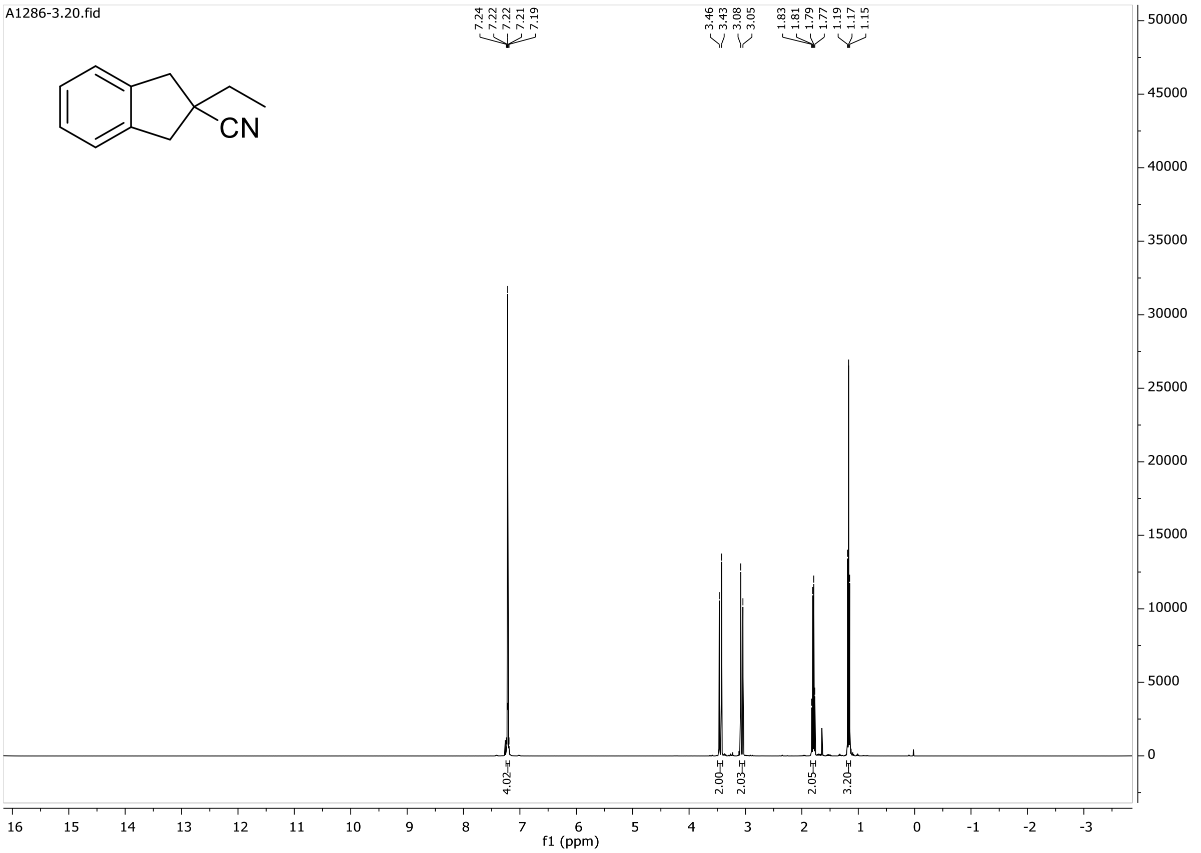
A1286-3.20.fid
CN
7.24
7.22
7.22
7.21
7.19
3.46
3.43
3.08
3.05
1.83
1.81
1.79
1.77
1.19
1.17
1.15
4.02
2.00
2.03
2.05
3.20
f1 (ppm)

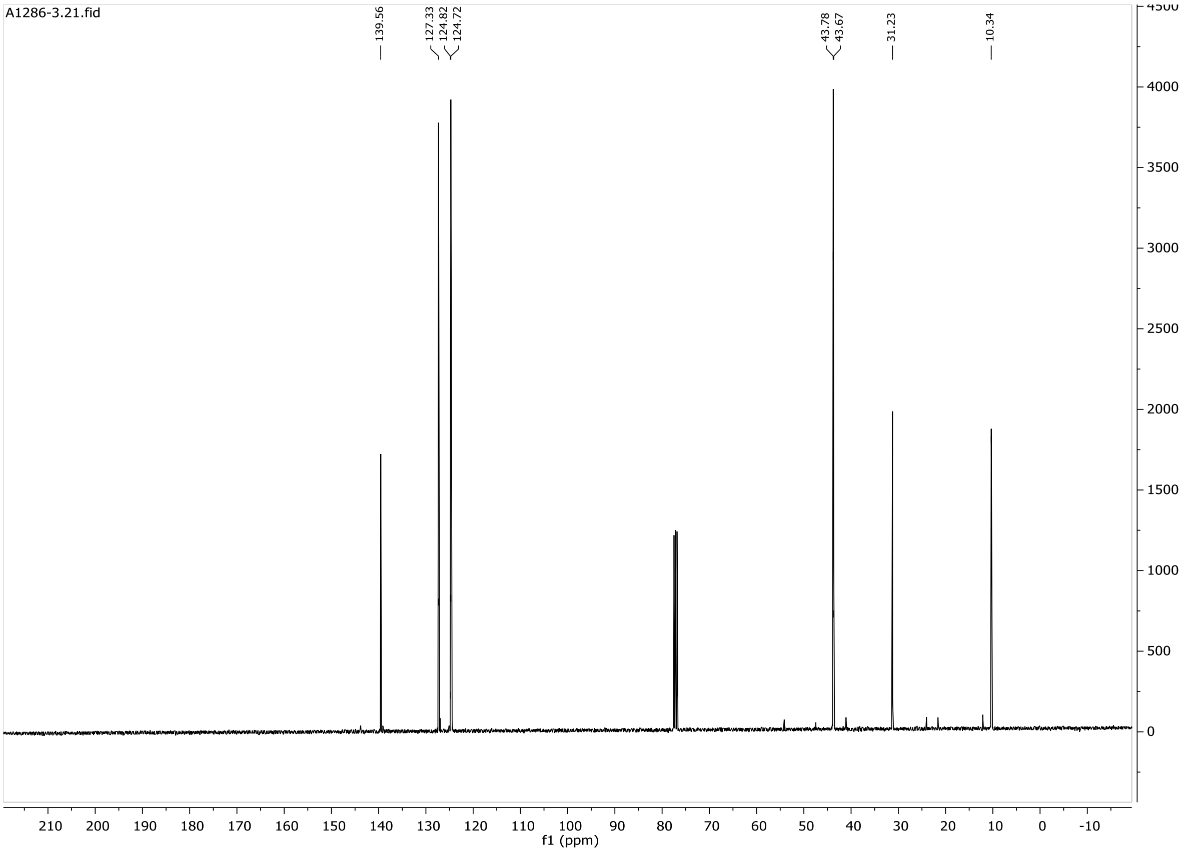
A1286-3.21.fid
139.56
127.33
124.82
124.72
43.78
43.67
31.23
10.34
f1 (ppm)

5

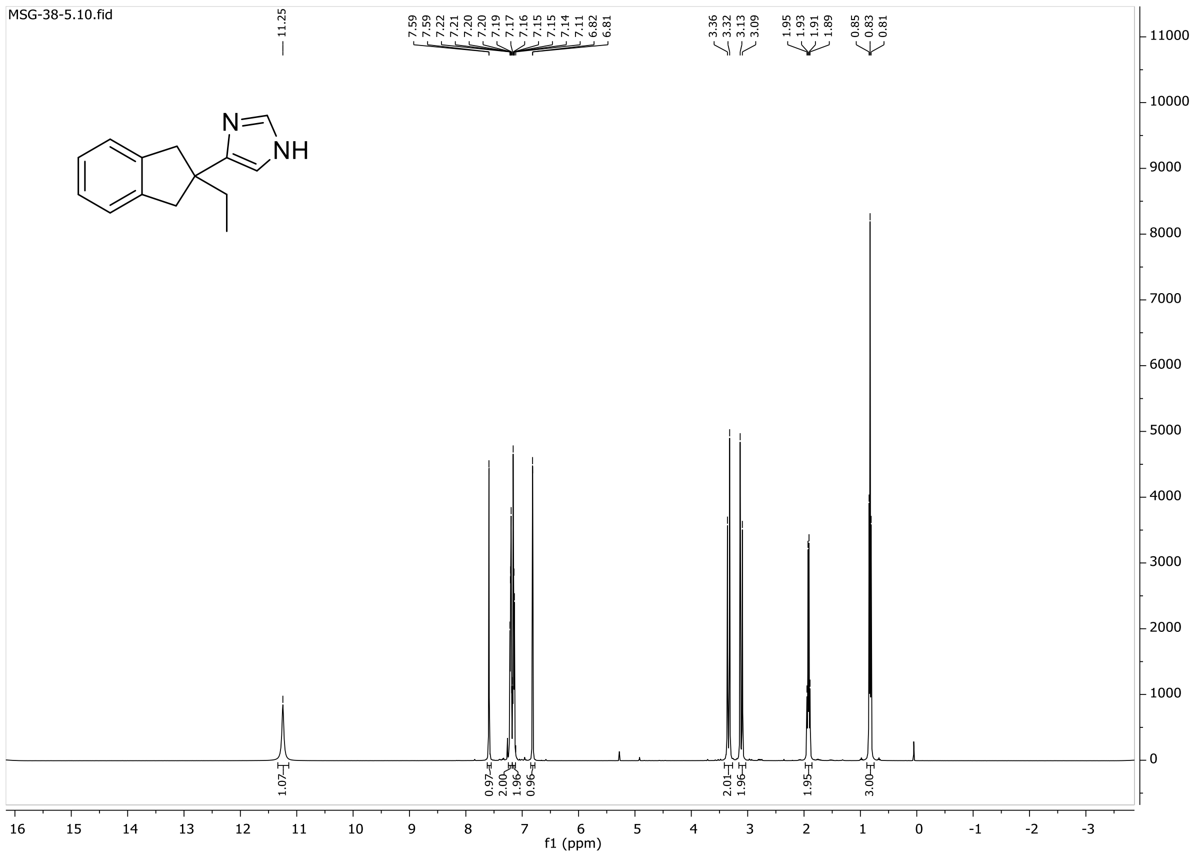

MSG-38-5.10.fid
N
NH
11.25
7.59
7.59
7.22
7.21
7.20
7.20
7.19
7.17
7.16
7.15
7.15
7.14
7.11
6.82
6.81
3.36
3.32
3.13
3.09
1.95
1.93
1.91
1.89
0.85
0.83
0.81
1.07
0.97
2.00
1.96
0.96
2.01
1.96
1.95
3.00
f1 (ppm)

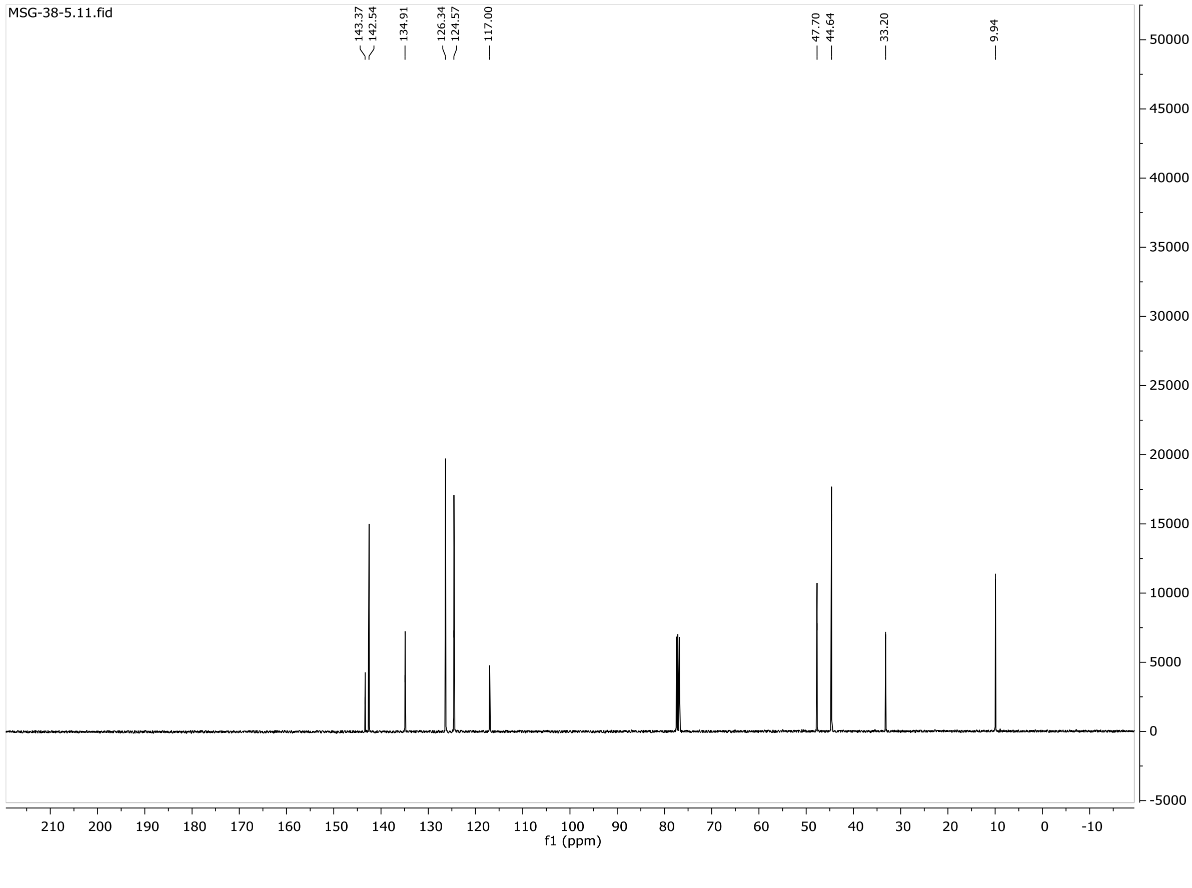

MSG-38-5.11.fid
143.37
142.54
134.91
126.34
124.57
117.00
47.70
44.64
33.20
9.94
f1 (ppm)

5-d_5

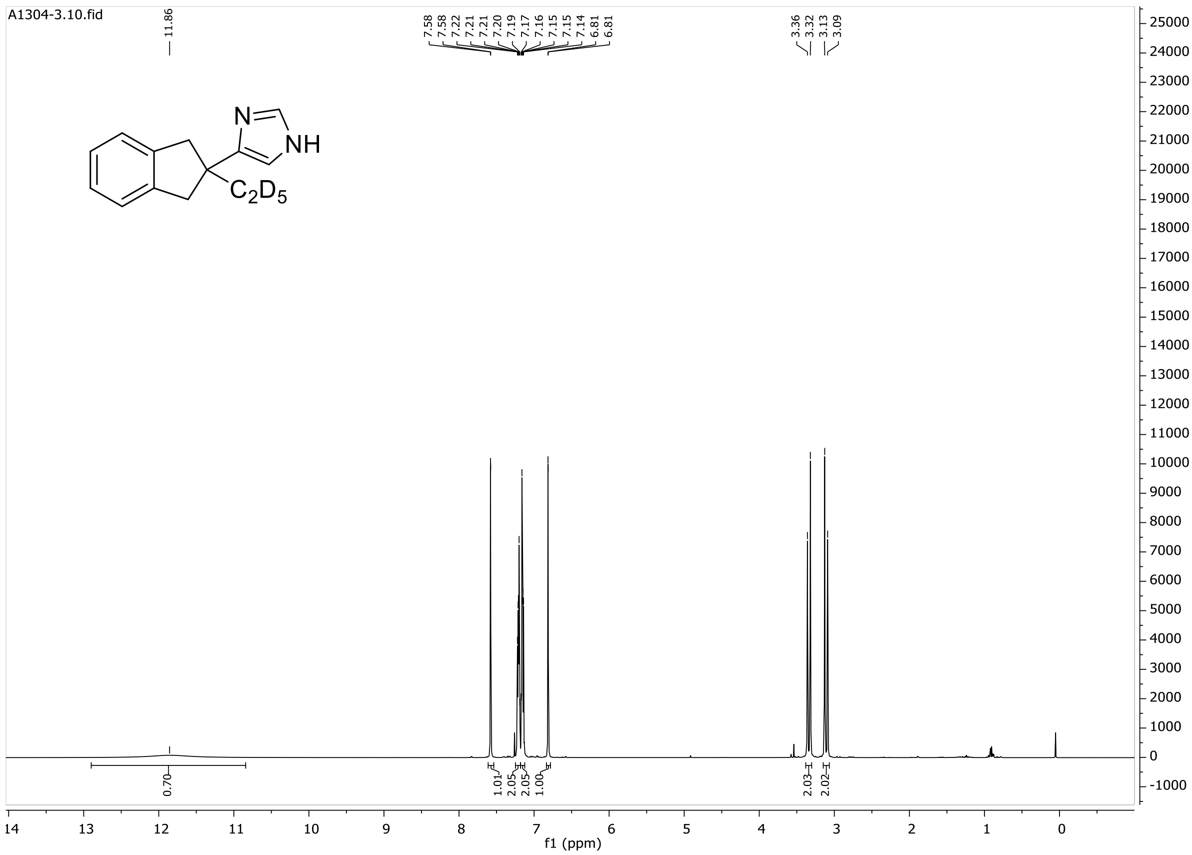
A1304-3.10.fid
11.86
7.58 7.58 7.22 7.21 7.21 7.20 7.19 7.17 7.16 7.15 7.15 7.14 6.81 6.81
3.36 3.32 3.13 3.09
N
NH
C_2D_5
0.70
1.01 2.05 2.05 1.00
2.03 2.02
f1 (ppm)

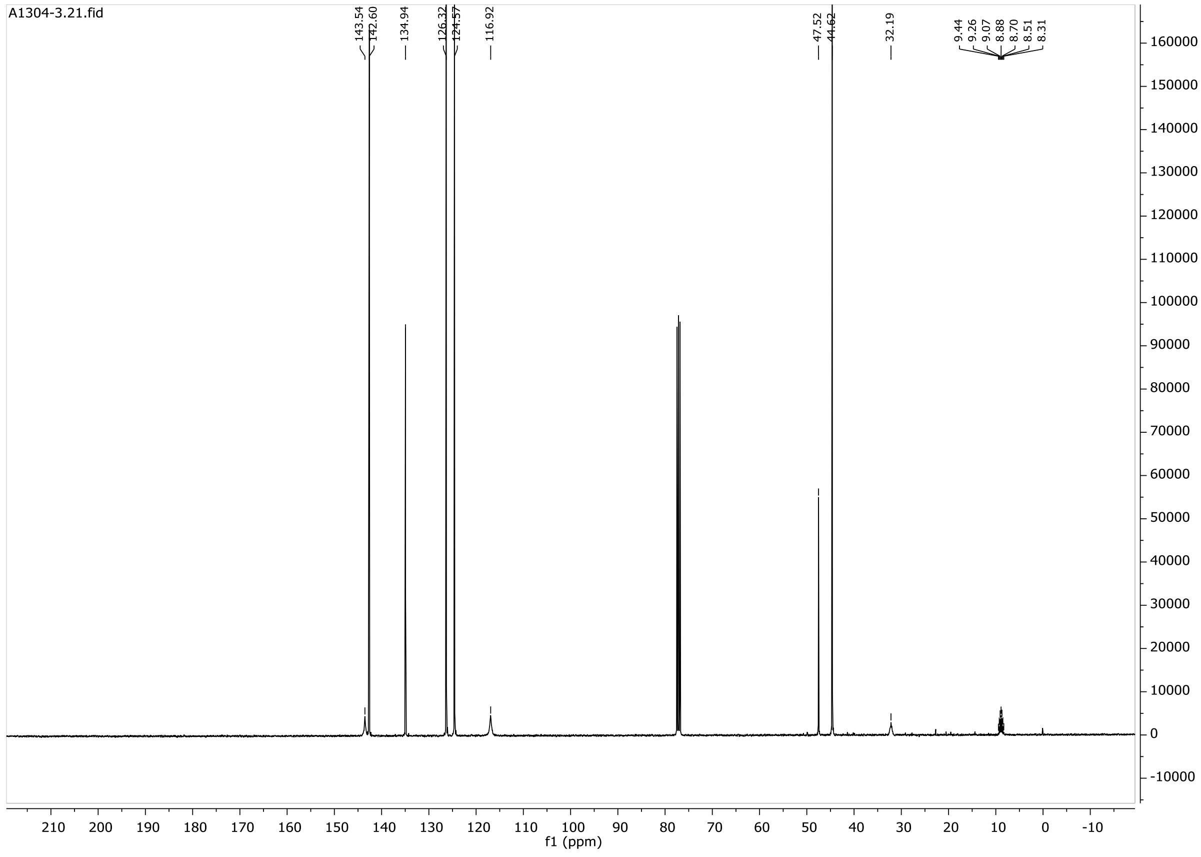
A1304-3.21.fid
143.54 142.60 134.94 126.32 124.57 116.92
47.52 44.62 32.19
9.44 9.26 9.07 8.88 8.70 8.51 8.31
f1 (ppm)

6

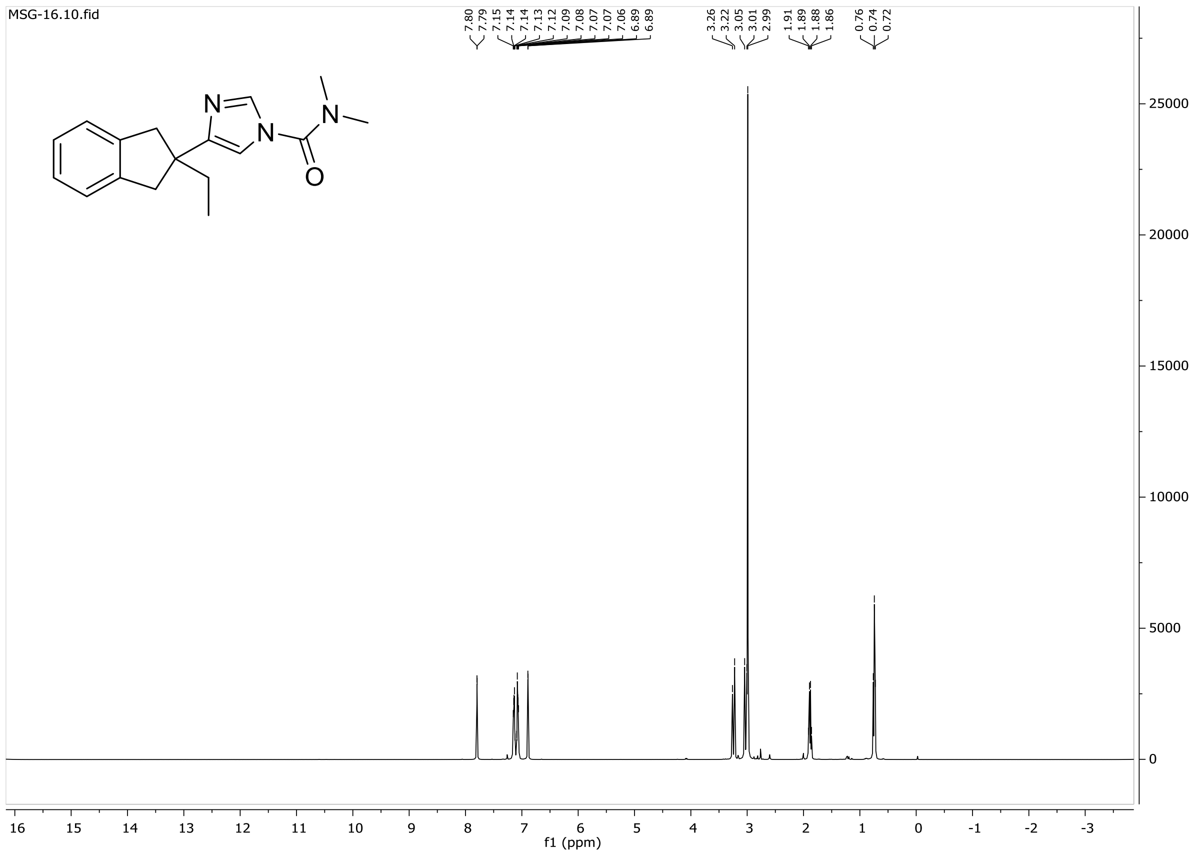
MSG-16.10.fid
7.80 7.79 7.15 7.14 7.14 7.13 7.12 7.09 7.08 7.07 7.07 7.06 6.89 6.89
3.26 3.22 3.05 3.01 2.99
1.91 1.89 1.88 1.86
0.76 0.74 0.72
f1 (ppm)

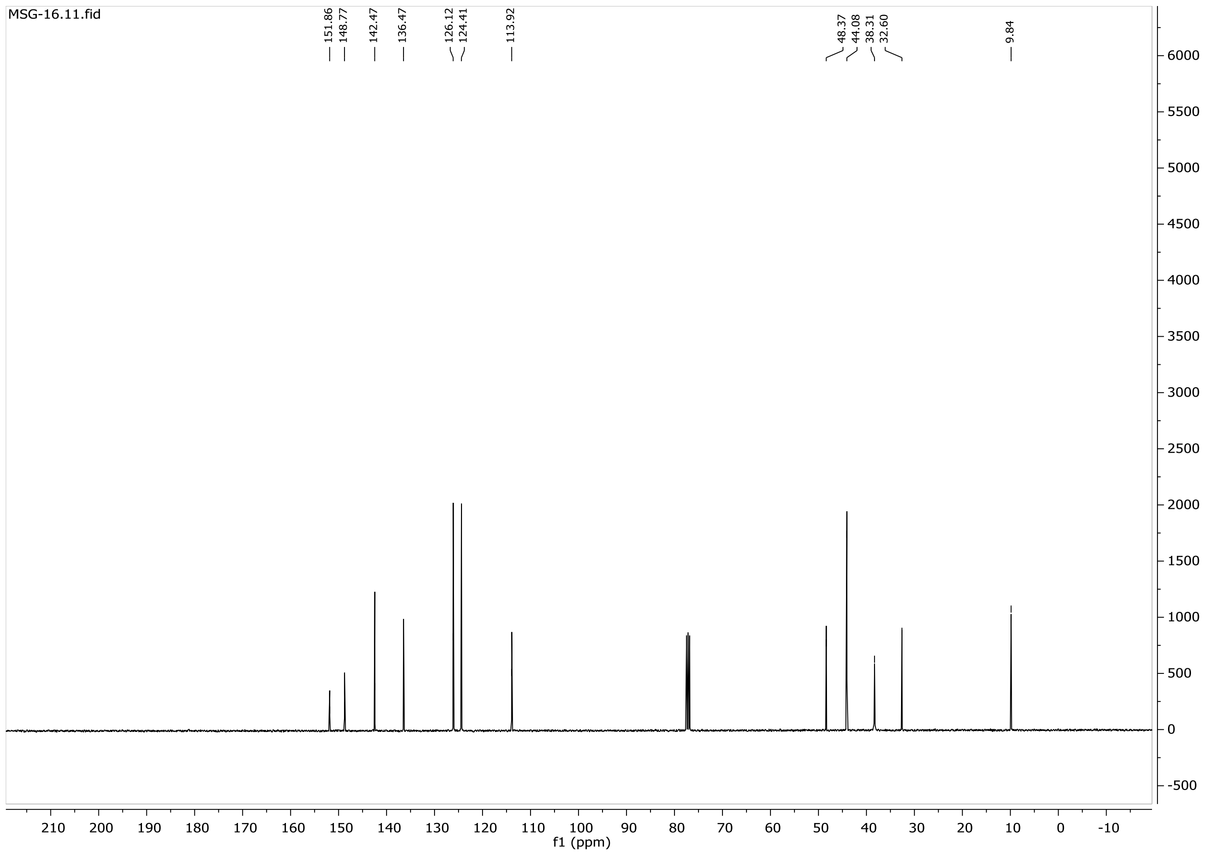
MSG-16.11.fid
151.86 148.77 142.47 136.47 126.12 124.41 113.92
48.37 44.08 38.31 32.60
9.84
f1 (ppm)

HMBC spectrum

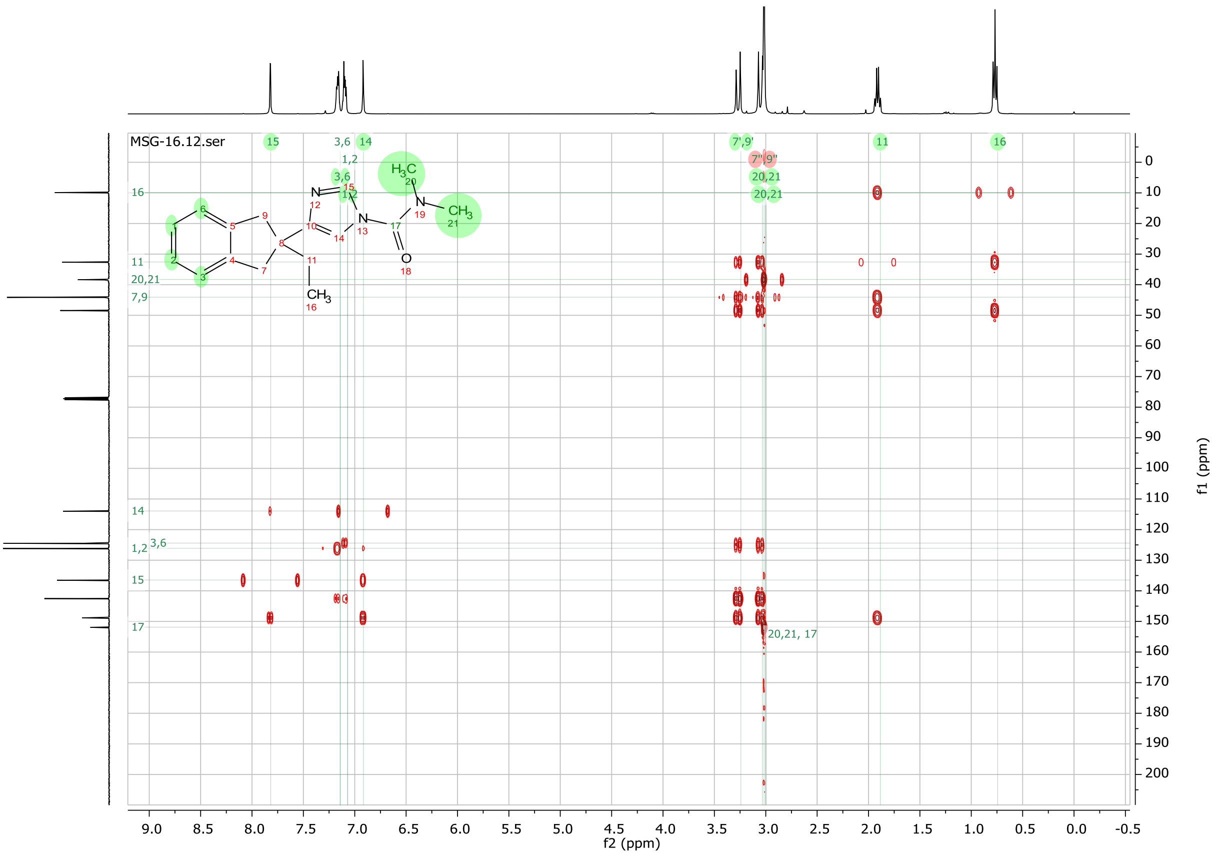

HSQC spectrum

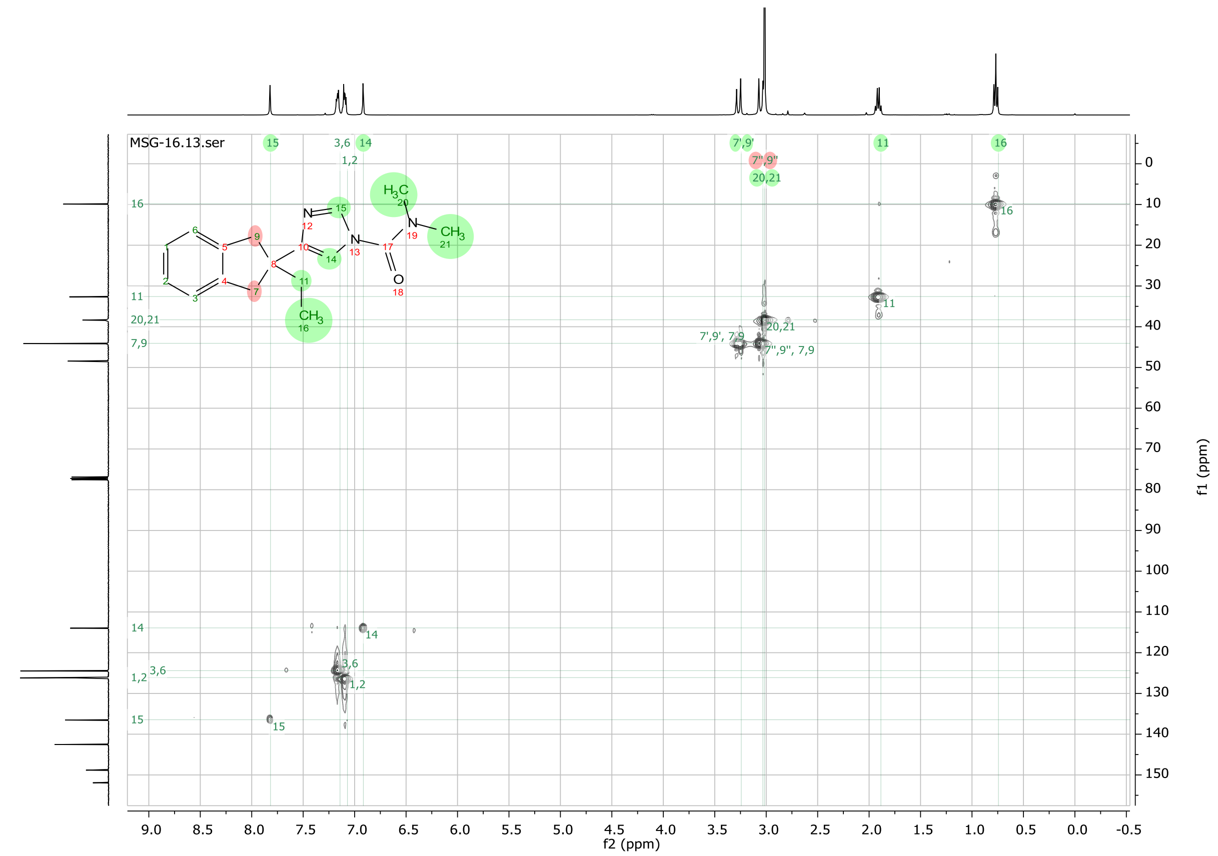

NOESY spectrum – imidazole C2 shows a crosspeak with carbamate *N*-methyls.

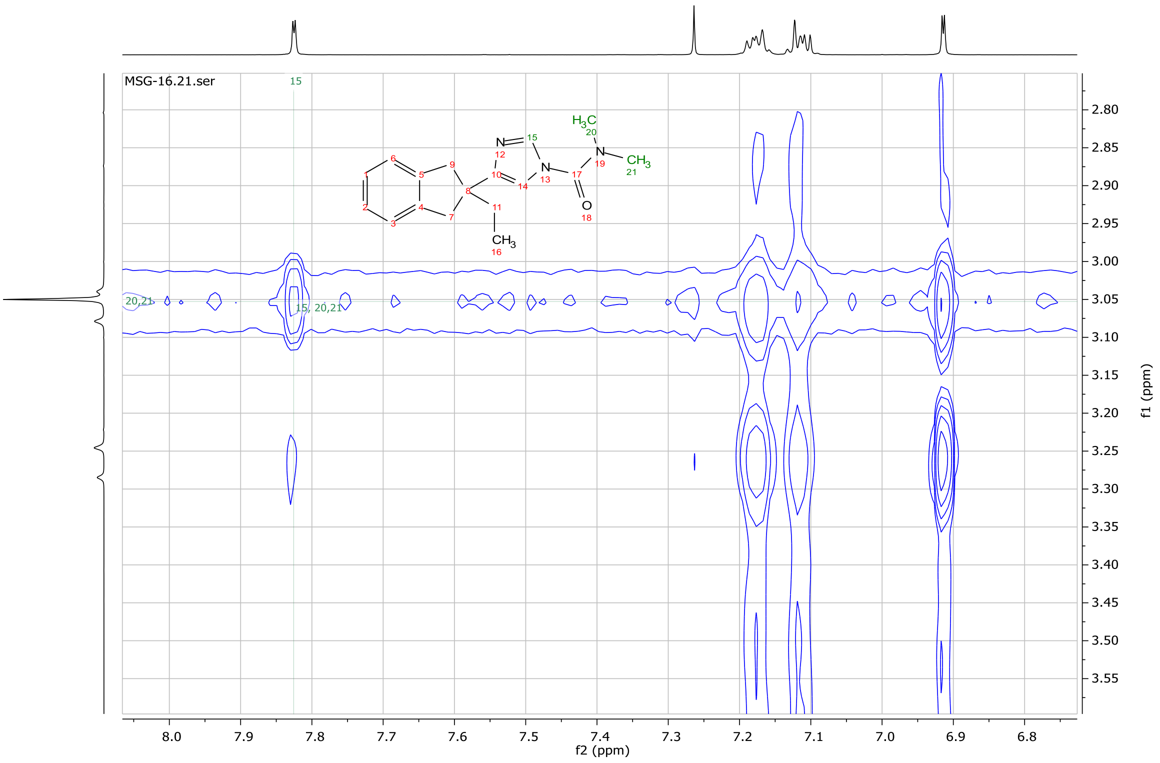

7

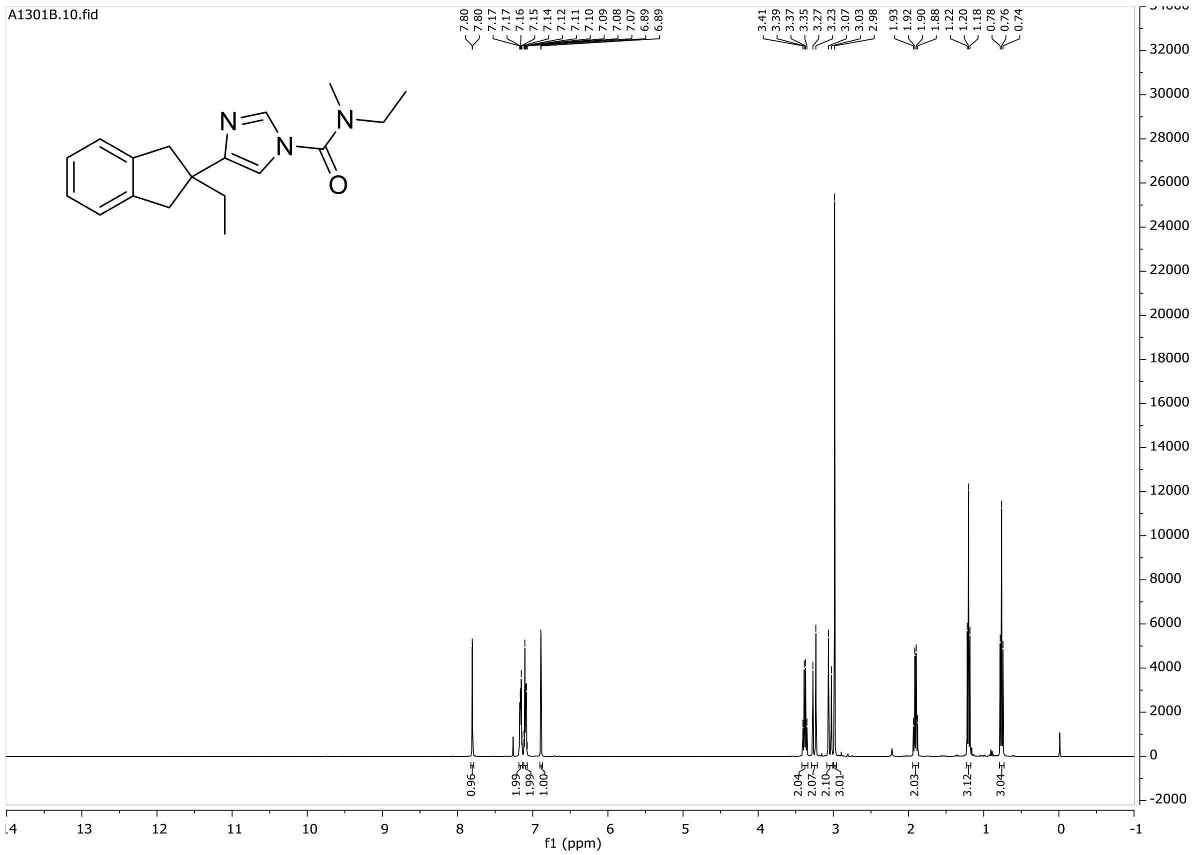
A1301B.10.fid
7.80
7.80
7.17
7.16
7.15
7.14
7.12
7.11
7.10
7.09
7.08
7.07
6.89
6.89
3.41
3.39
3.37
3.35
3.27
3.23
3.07
3.03
2.98
1.93
1.92
1.90
1.88
1.22
1.20
1.18
0.78
0.76
0.74
0.96
1.99
1.99
1.00
2.04
2.07
2.10
3.01
2.03
3.12
3.04
f1 (ppm)

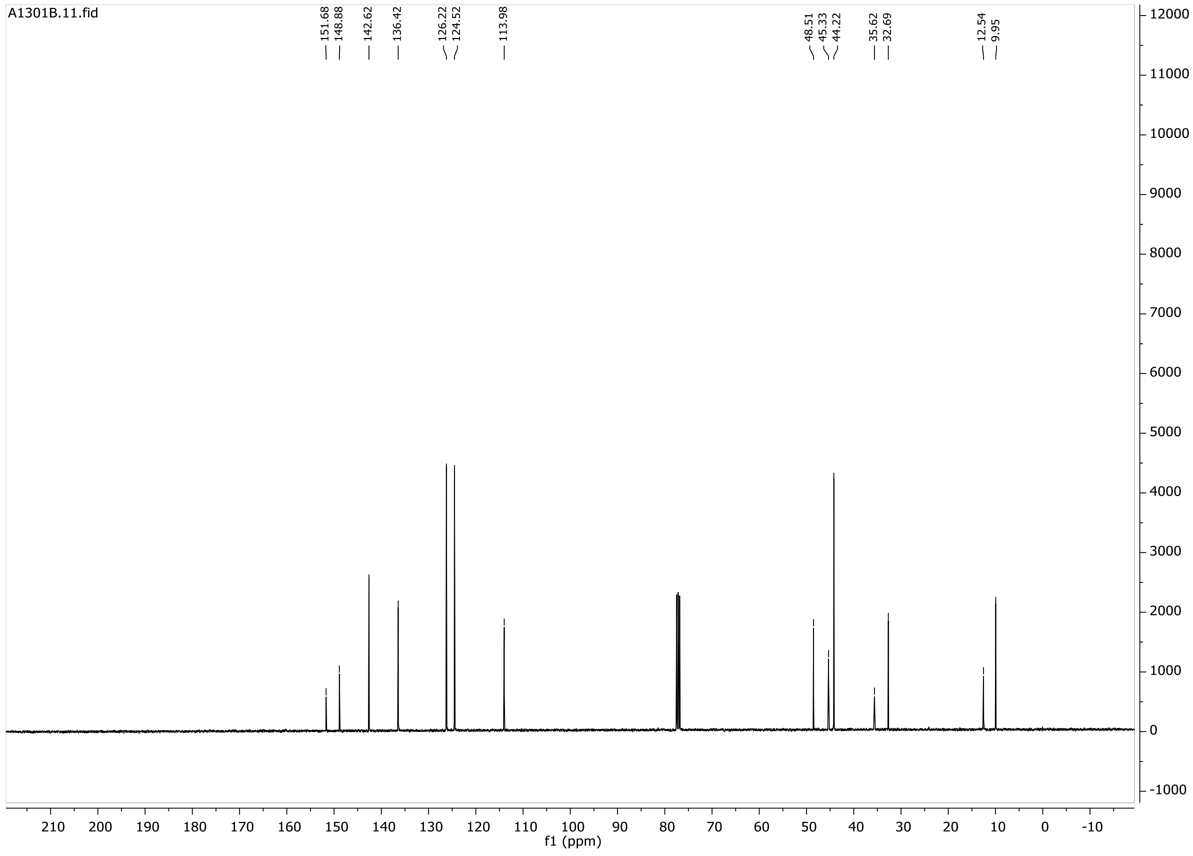
A1301B.11.fid
151.68
148.88
142.62
136.42
126.22
124.52
113.98
48.51
45.33
44.22
35.62
32.69
12.54
9.95
f1 (ppm)

8

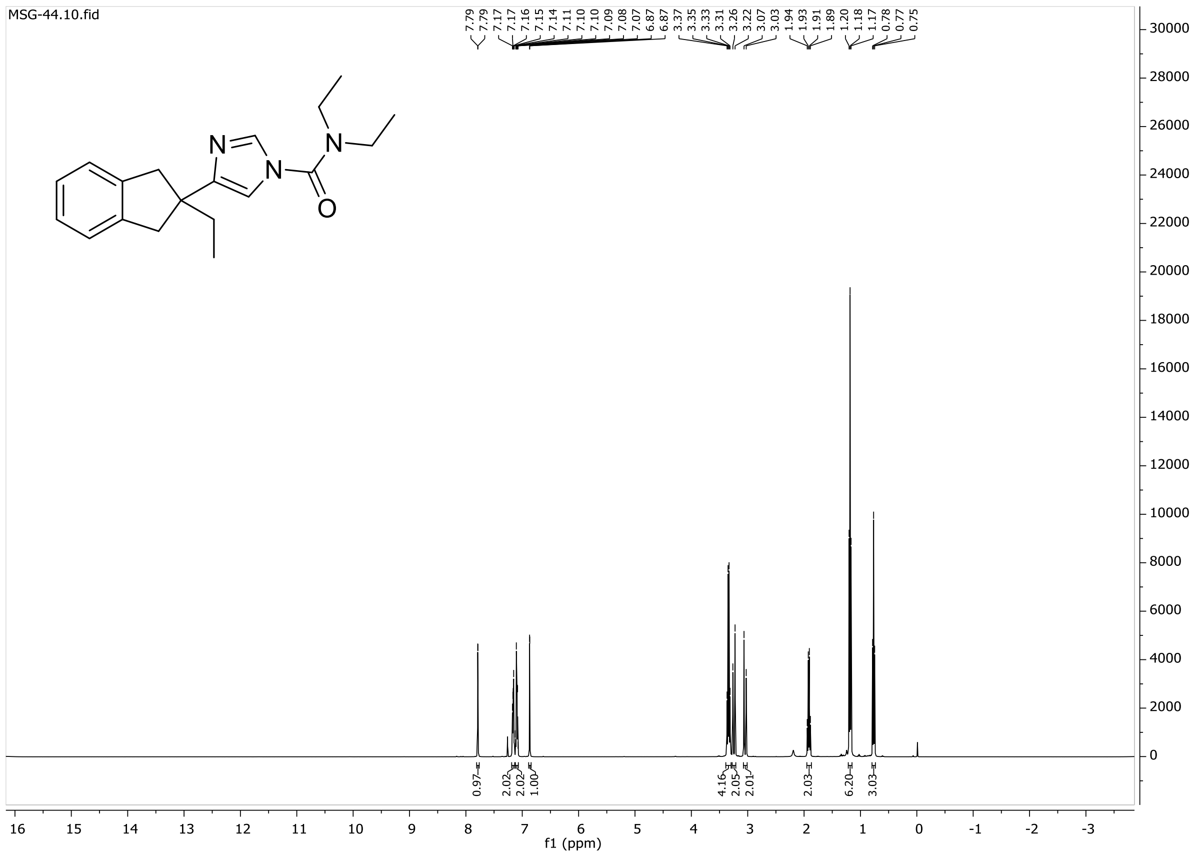

MSG-44.10.fid
7.79
7.79
7.17
7.16
7.15
7.14
7.11
7.10
7.09
7.08
7.07
6.87
6.87
3.37
3.35
3.33
3.31
3.26
3.22
3.07
3.03
1.94
1.93
1.91
1.89
1.20
1.18
1.17
0.78
0.77
0.75
0.97
2.02
2.02
1.00
4.16
2.05
2.01
2.03
6.20
3.03
f1 (ppm)

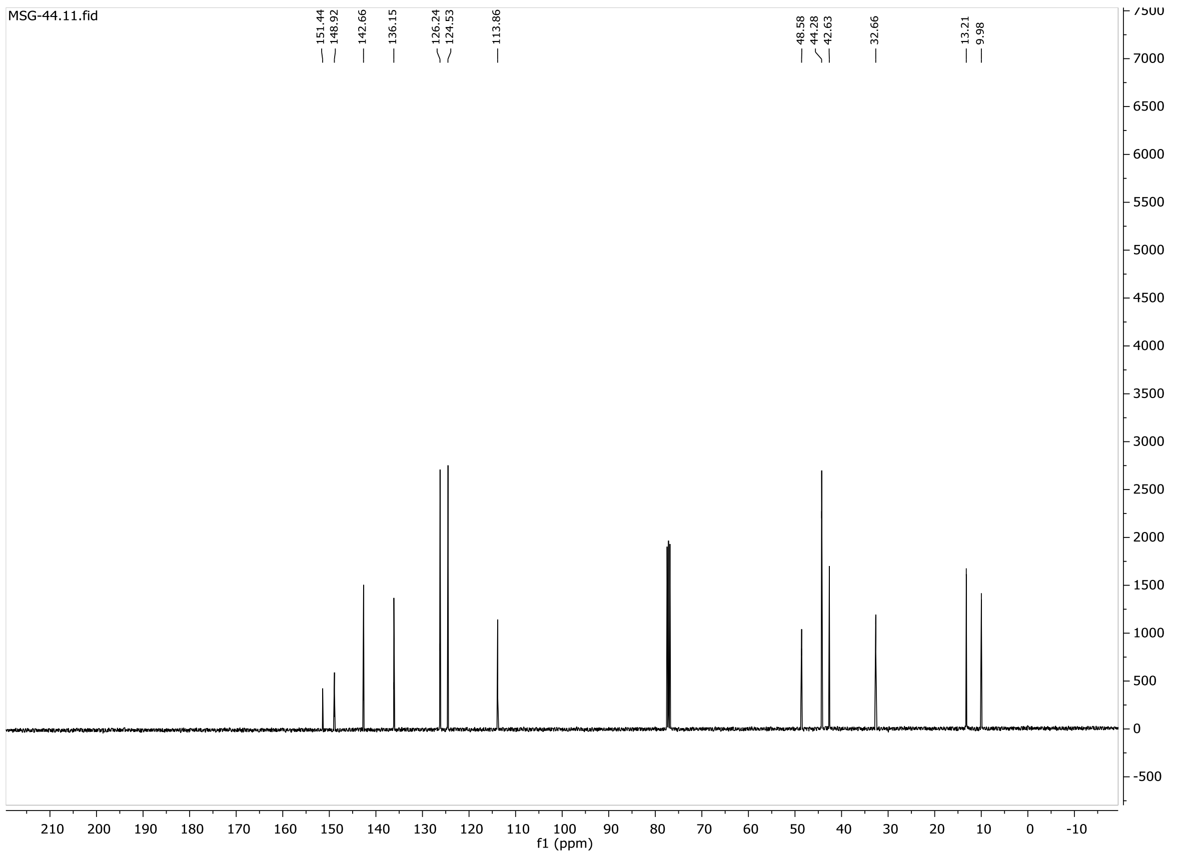

MSG-44.11.fid
151.44
148.92
142.66
136.15
126.24
124.53
113.86
48.58
44.28
42.63
32.66
13.21
9.98
f1 (ppm)

8-*d*5

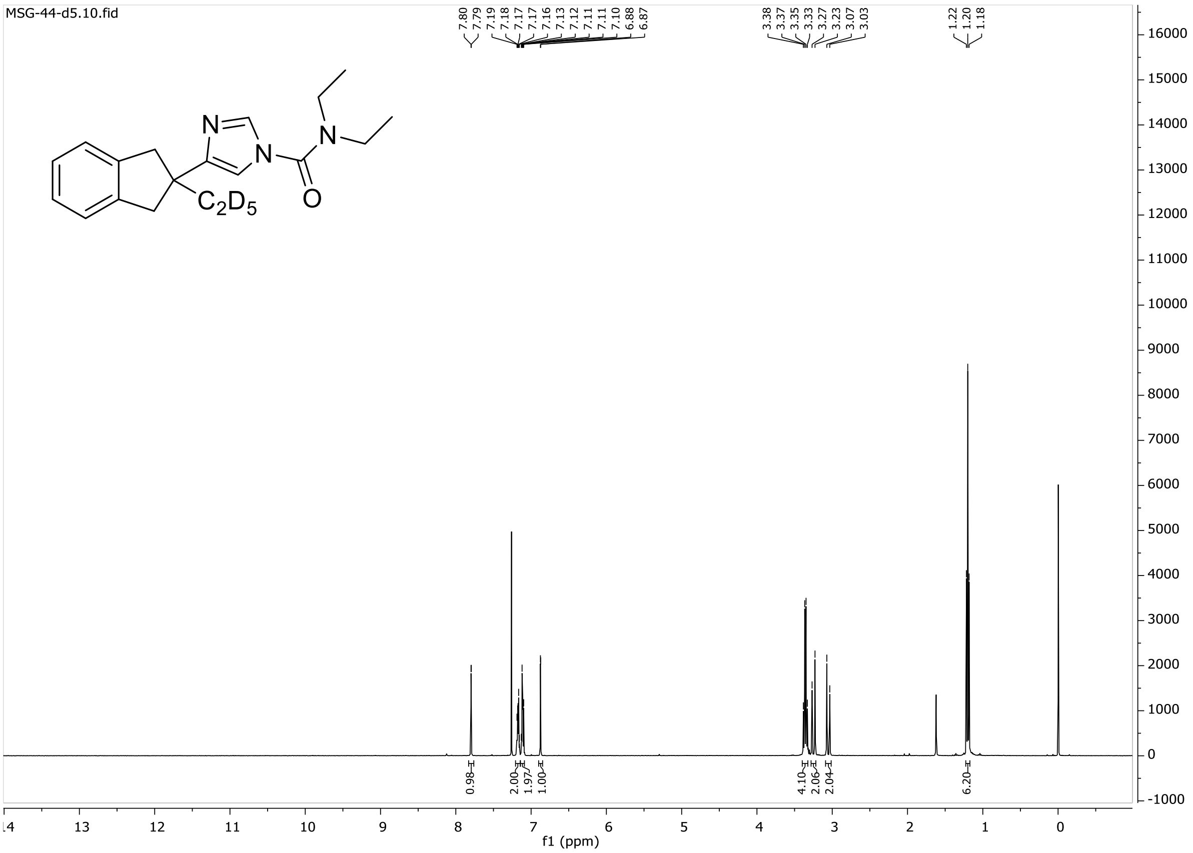
MSG-44-d5.10.fid
7.80
7.79
7.19
7.18
7.17
7.17
7.16
7.13
7.12
7.11
7.11
7.10
6.88
6.87
3.38
3.37
3.35
3.33
3.27
3.23
3.07
3.03
1.22
1.20
1.18
N
N
N
O
C2D5
0.98
2.00
1.97
1.00
4.10
2.06
2.04
6.20
f1 (ppm)

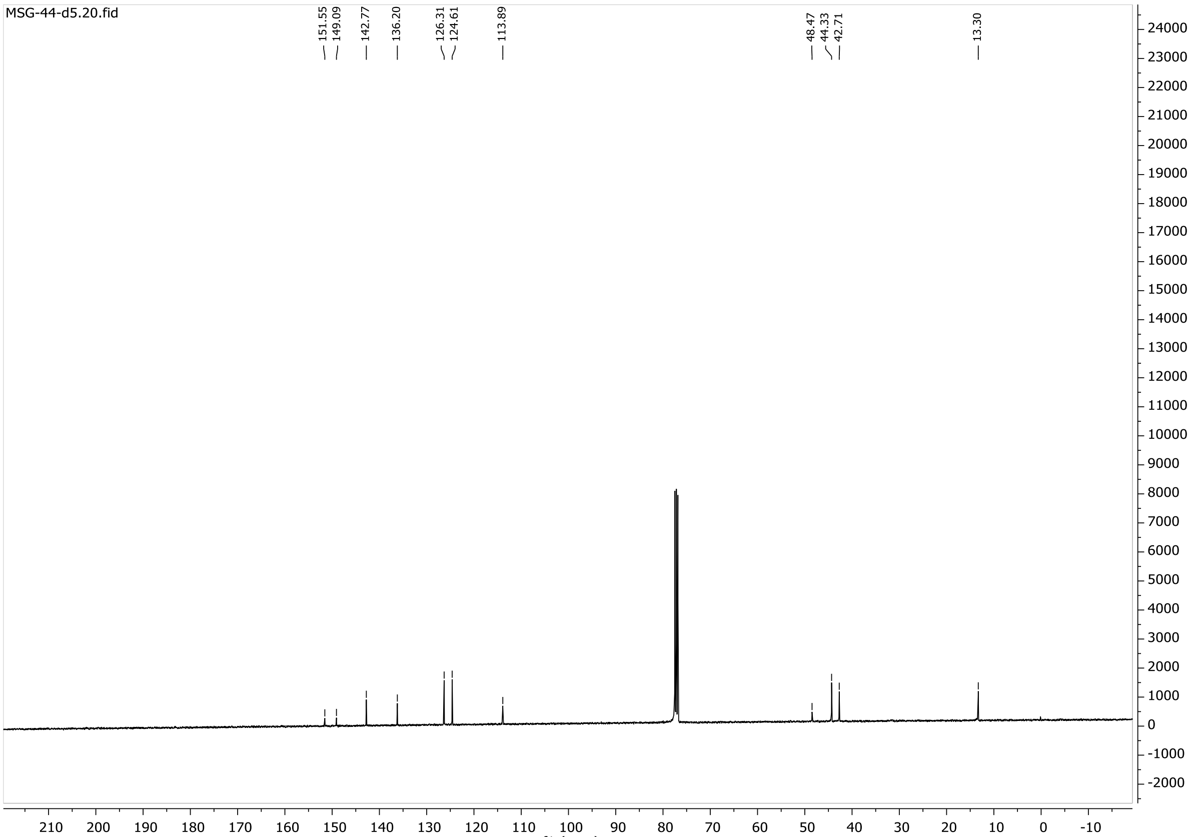
MSG-44-d5.20.fid
151.55
149.09
142.77
136.20
126.31
124.61
113.89
48.47
44.33
42.71
13.30
f1 (ppm)

9

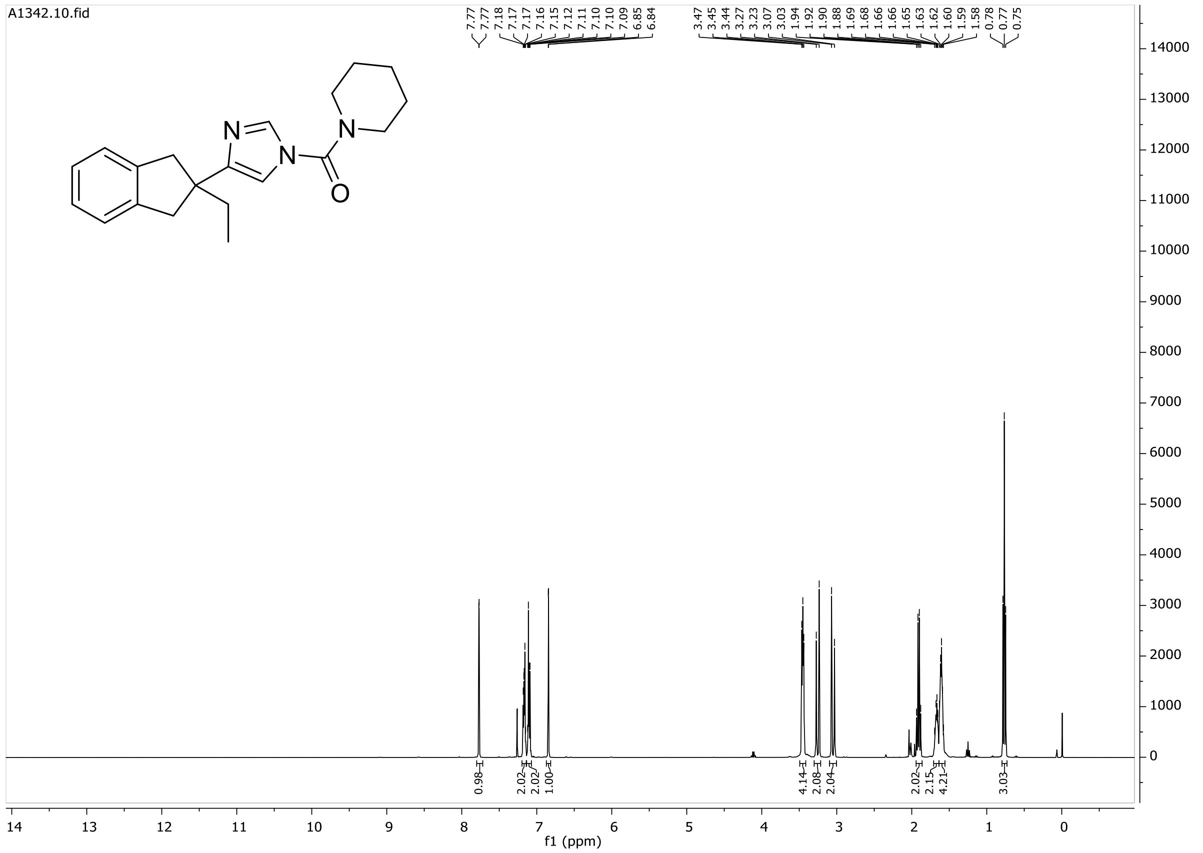
A1342.10.fid
7.77
7.77
7.18
7.17
7.17
7.16
7.15
7.12
7.11
7.10
7.10
7.09
6.85
6.84
3.47
3.45
3.44
3.27
3.23
3.07
3.03
1.94
1.92
1.90
1.88
1.69
1.68
1.66
1.66
1.65
1.63
1.62
1.60
1.59
1.58
0.78
0.77
0.75
0.98
2.02
2.02
1.00
4.14
2.08
2.04
2.02
2.15
4.21
3.03
f1 (ppm)

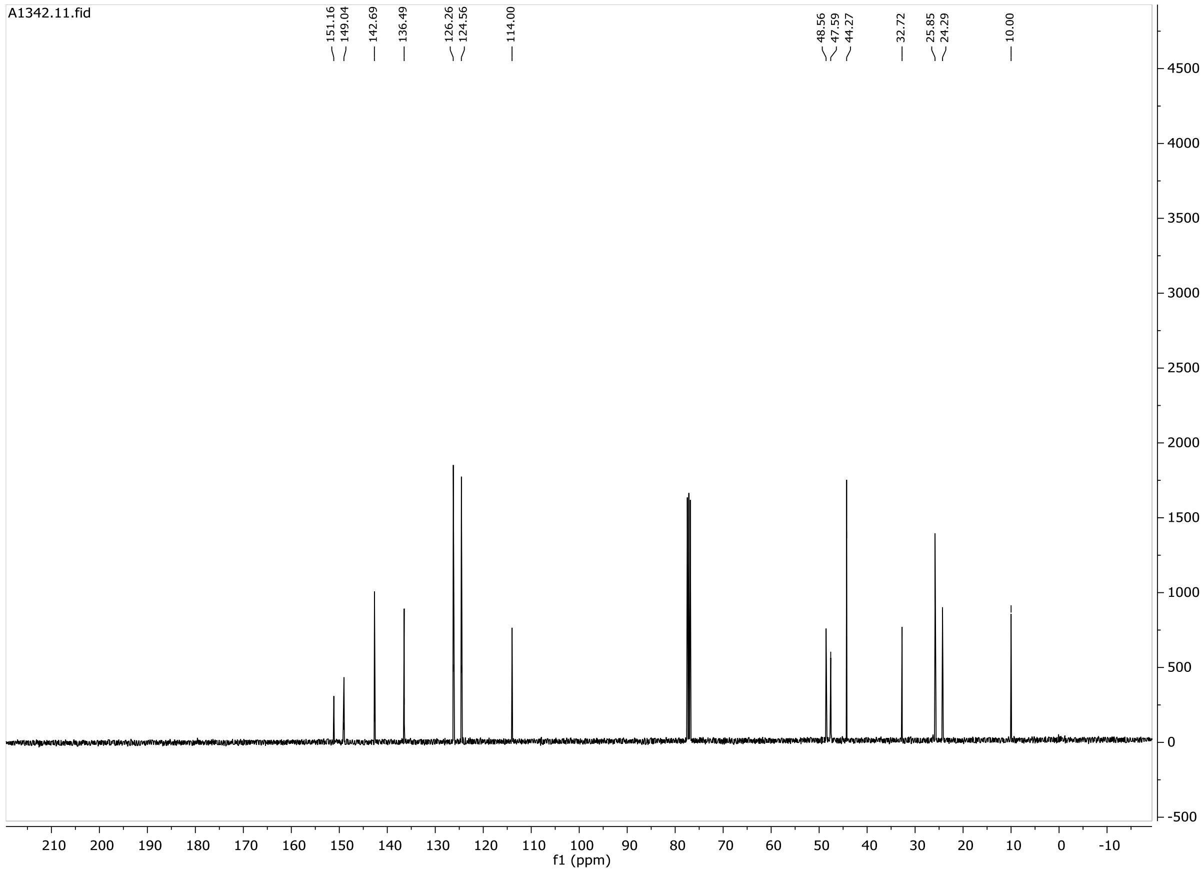
A1342.11.fid
151.16
149.04
142.69
136.49
126.26
124.56
114.00
48.56
47.59
44.27
32.72
25.85
24.29
10.00
f1 (ppm)

10

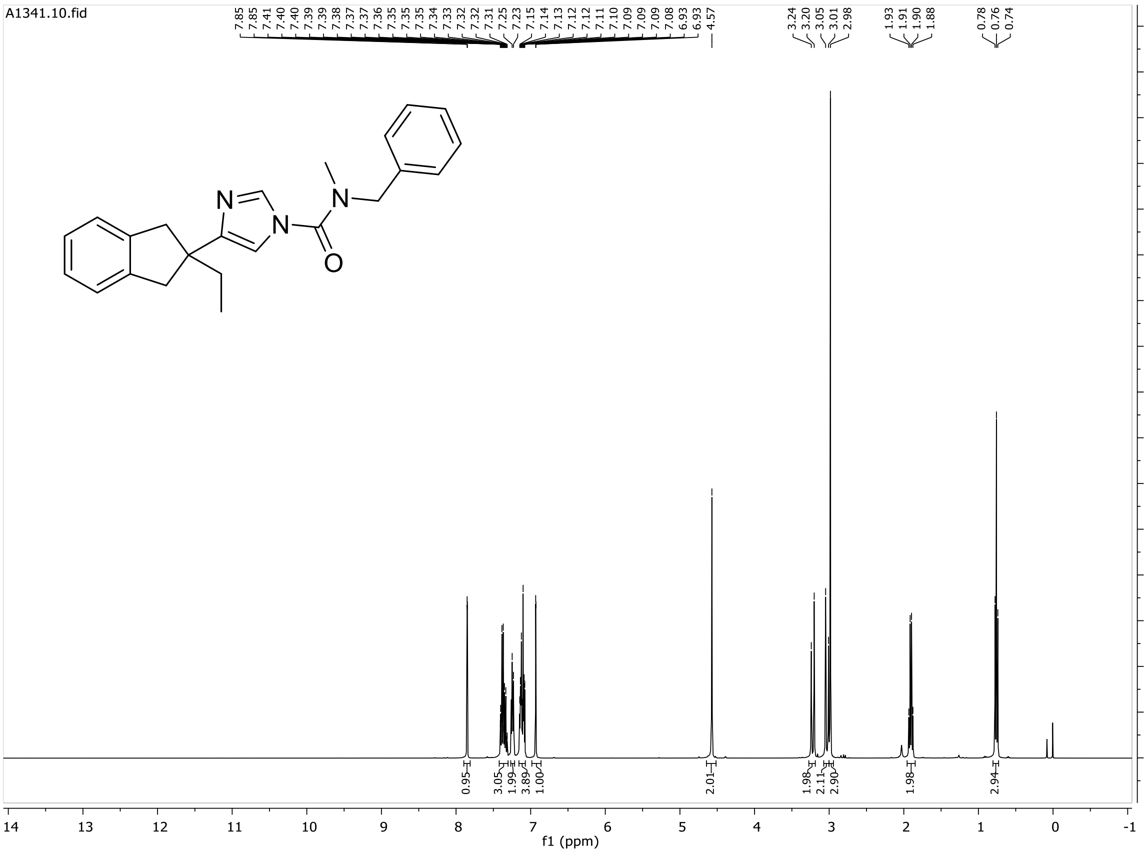
A1341.10.fid
7.85 7.85 7.41 7.40 7.39 7.39 7.38 7.37 7.37 7.36 7.35 7.35 7.35 7.34 7.33 7.32 7.32 7.31 7.25 7.23 7.15 7.14 7.13 7.12 7.12 7.11 7.10 7.09 7.09 7.09 7.08 6.93 6.93 4.57
3.24 3.20 3.05 3.01 2.98
1.93 1.91 1.90 1.88
0.78 0.76 0.74
N
N
N
O
0.95 3.05 1.99 3.89 1.00 2.01 1.98 2.11 2.90 1.98 2.94
f1 (ppm)

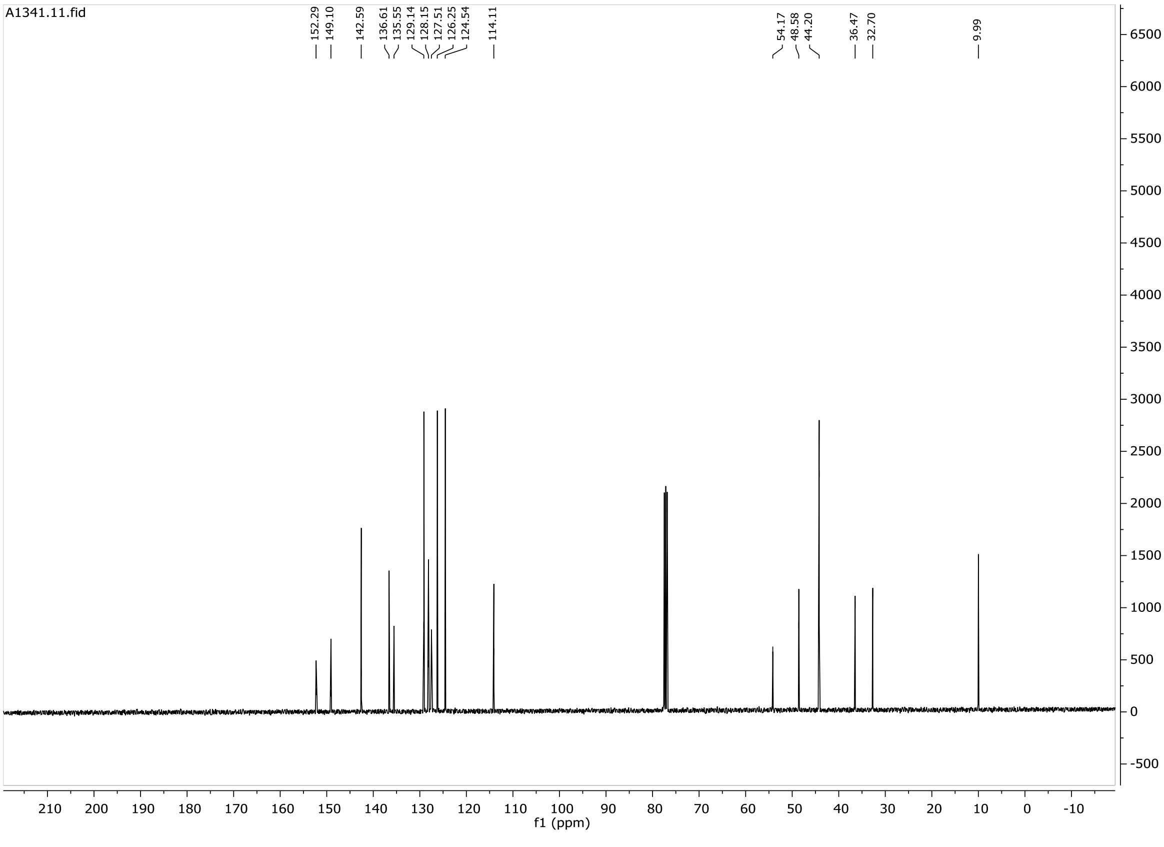
A1341.11.fid
152.29 149.10 142.59 136.61 135.55 129.14 128.15 127.51 126.25 124.54 114.11
54.17 48.58 44.20 36.47 32.70 9.99
f1 (ppm)

11

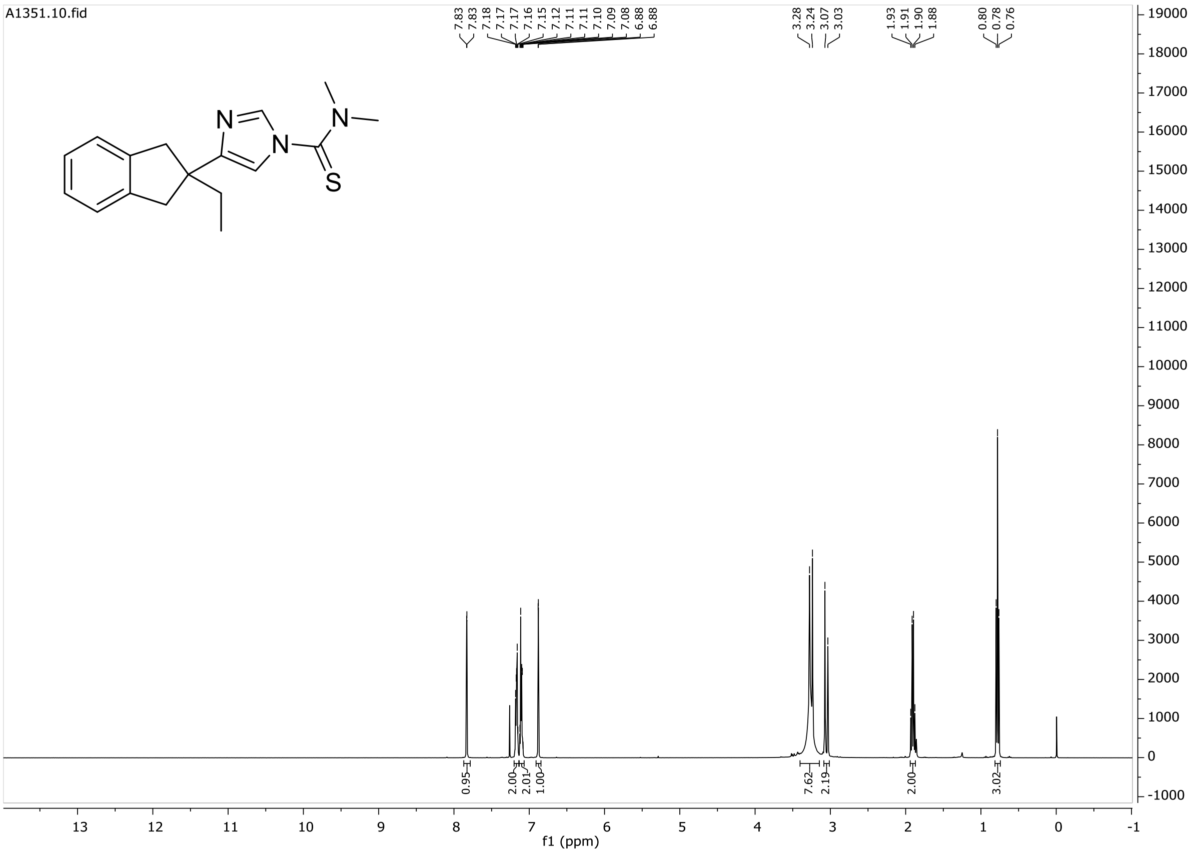
A1351.10.fid
7.83
7.83
7.18
7.17
7.16
7.15
7.12
7.11
7.11
7.10
7.09
7.08
6.88
6.88
3.28
3.24
3.07
3.03
1.93
1.91
1.90
1.88
0.80
0.78
0.76
0.95
2.00
2.01
1.00
7.62
2.19
2.00
3.02
f1 (ppm)

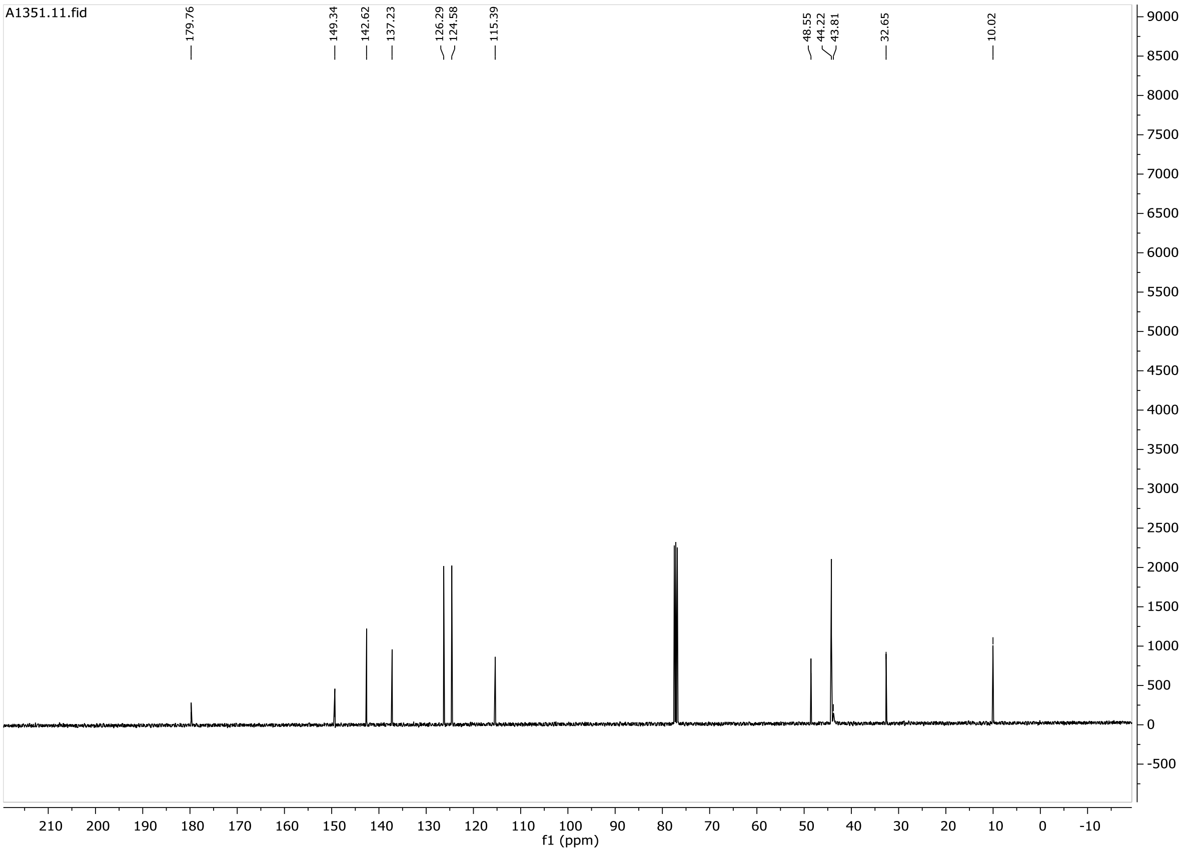
A1351.11.fid
179.76
149.34
142.62
137.23
126.29
124.58
115.39
48.55
44.22
43.81
32.65
10.02
f1 (ppm)

12

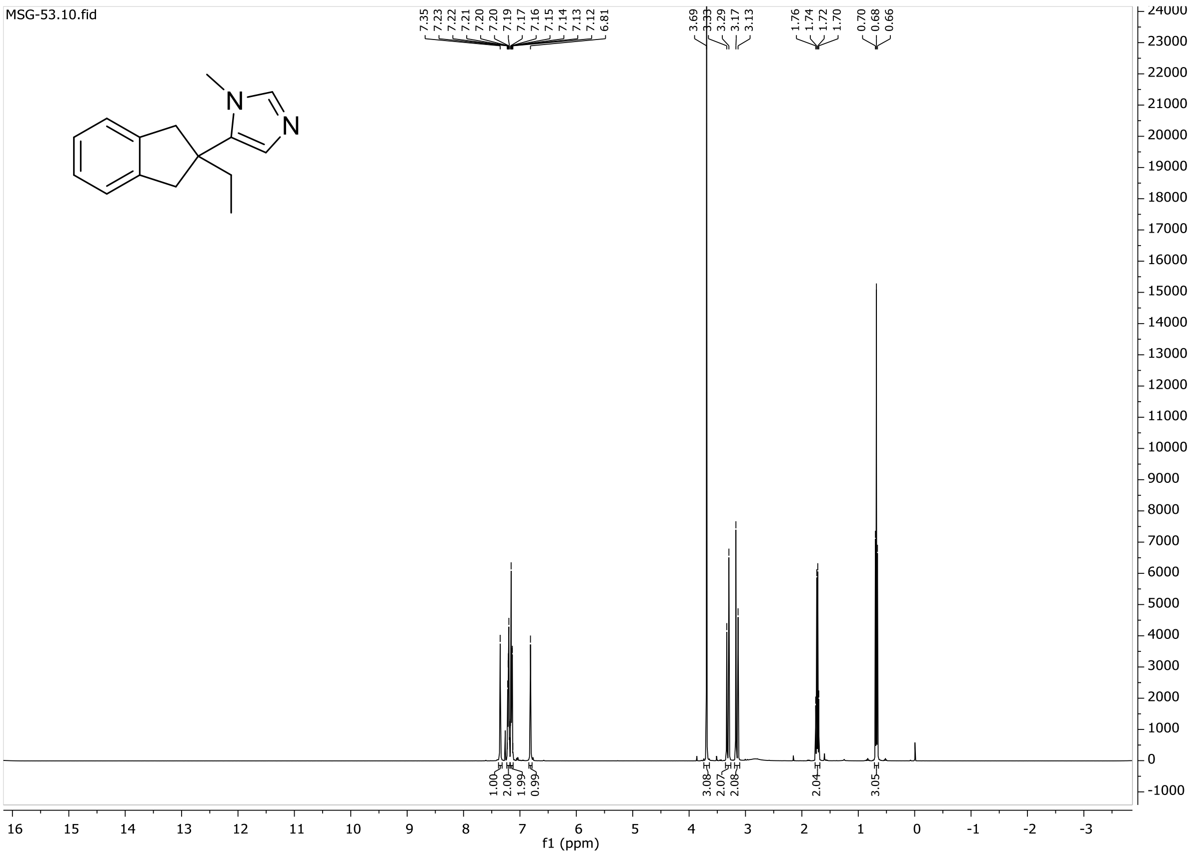

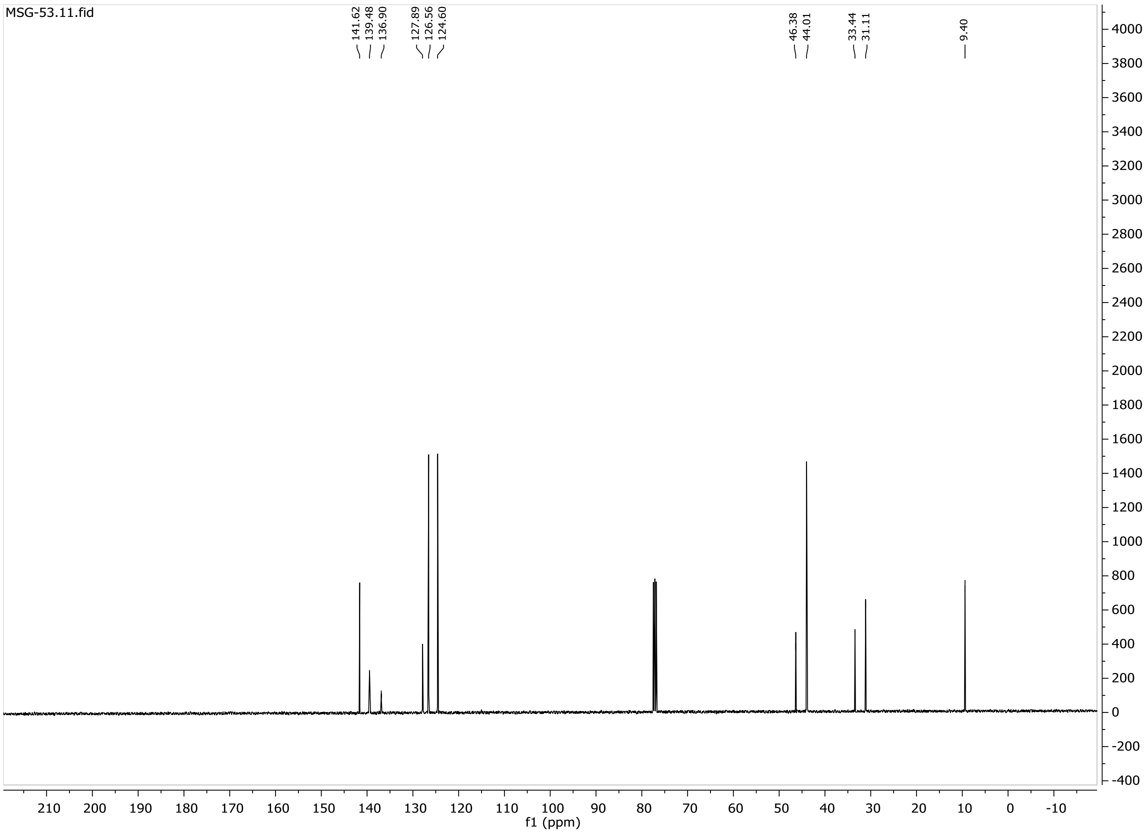

NOESY spectrum – an interaction between atom 11 ($\underline{CH_2}CH_3$) and 17 ($N\underline{CH_3}$) is observed, which is only possible with the 1,5-substitution pattern.

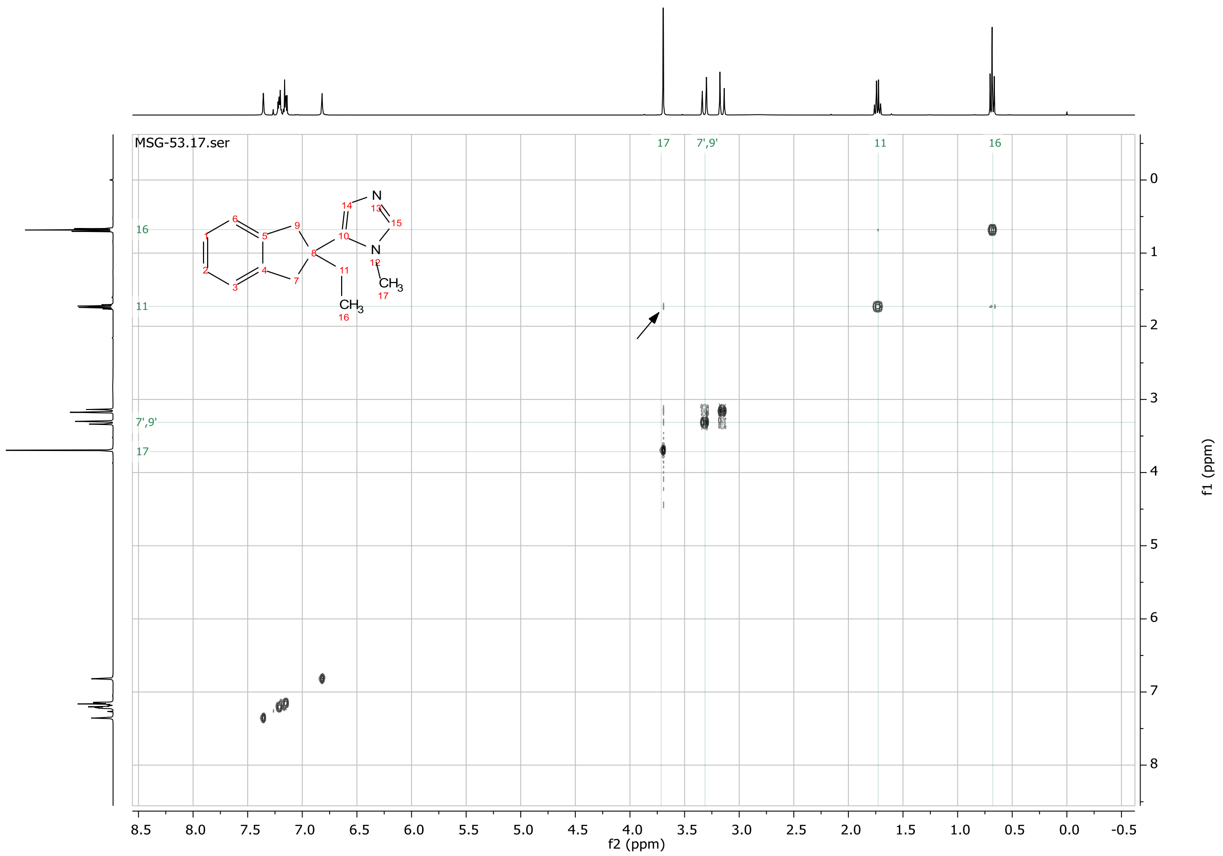

HSQC spectrum

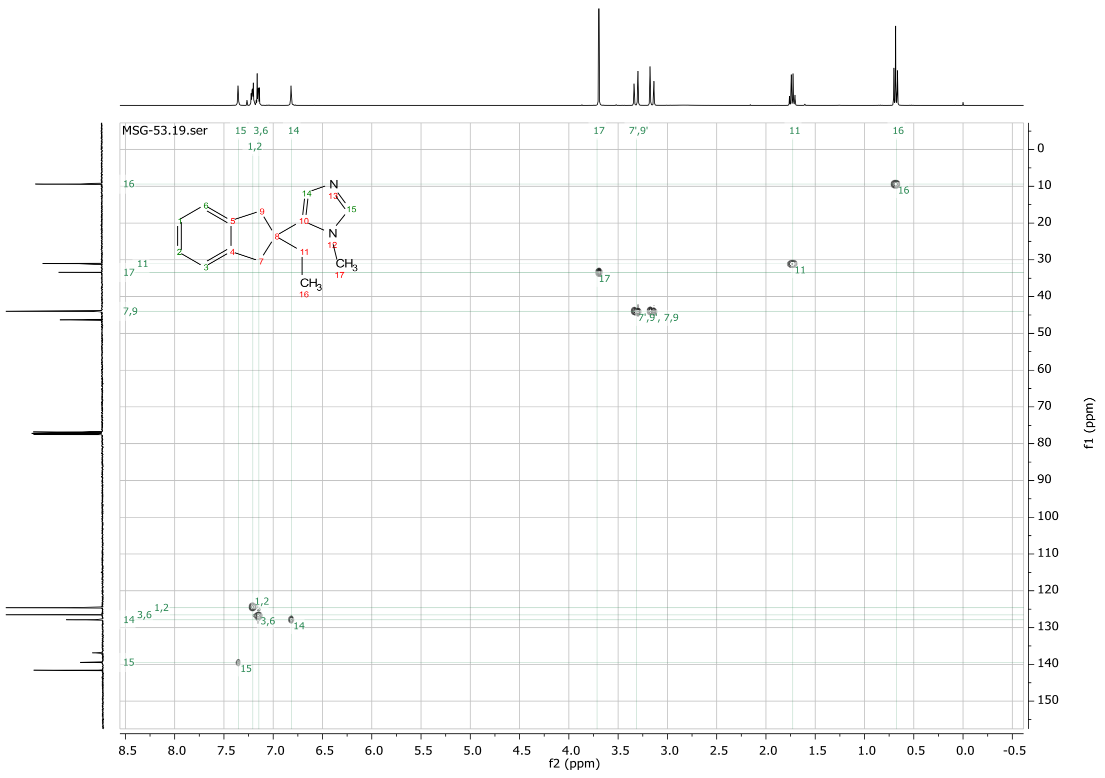

13

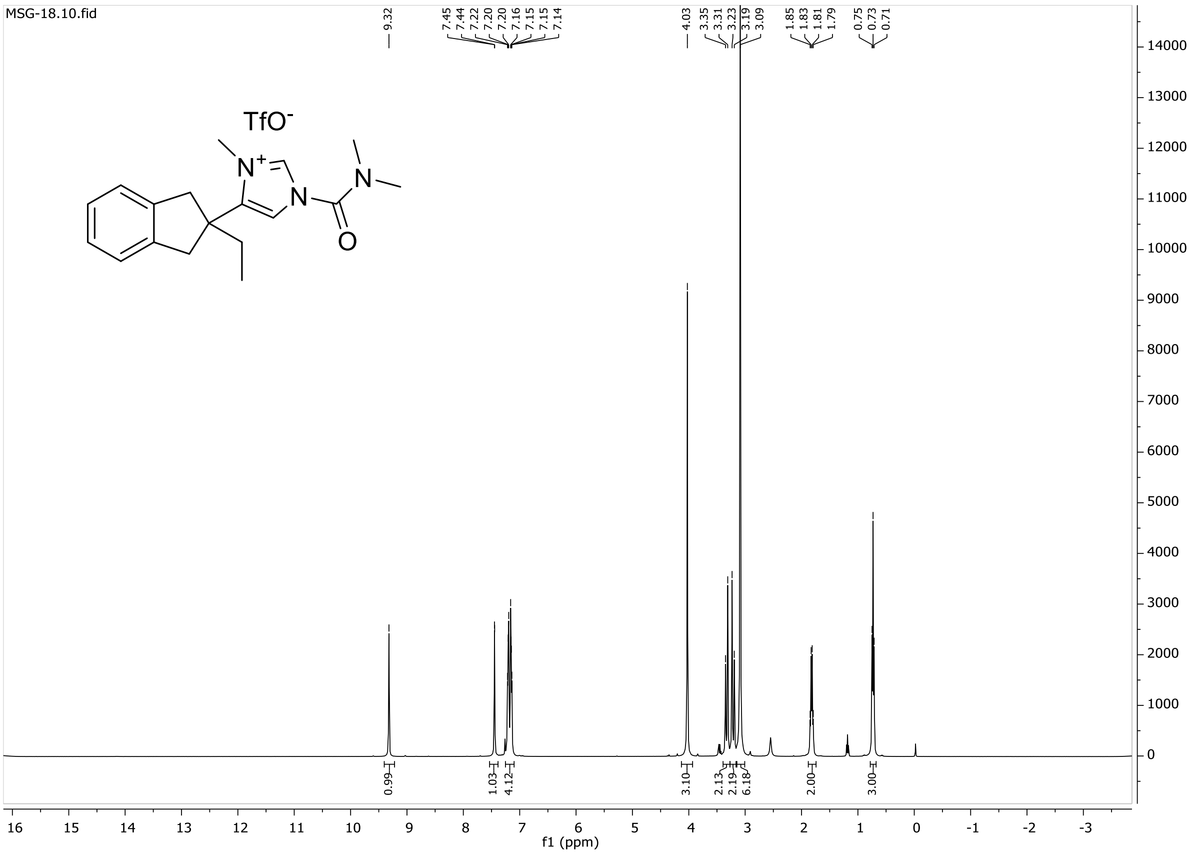
MSG-18.10.fid
9.32
7.45 7.44 7.22 7.20 7.20 7.16 7.15 7.15 7.14
4.03 3.35 3.31 3.23 3.19 3.09
1.85 1.83 1.81 1.79
0.75 0.73 0.71
TfO-
N+
N
N
O
0.99 1.03 4.12 3.10 2.13 2.19 6.18 2.00 3.00
f1 (ppm)

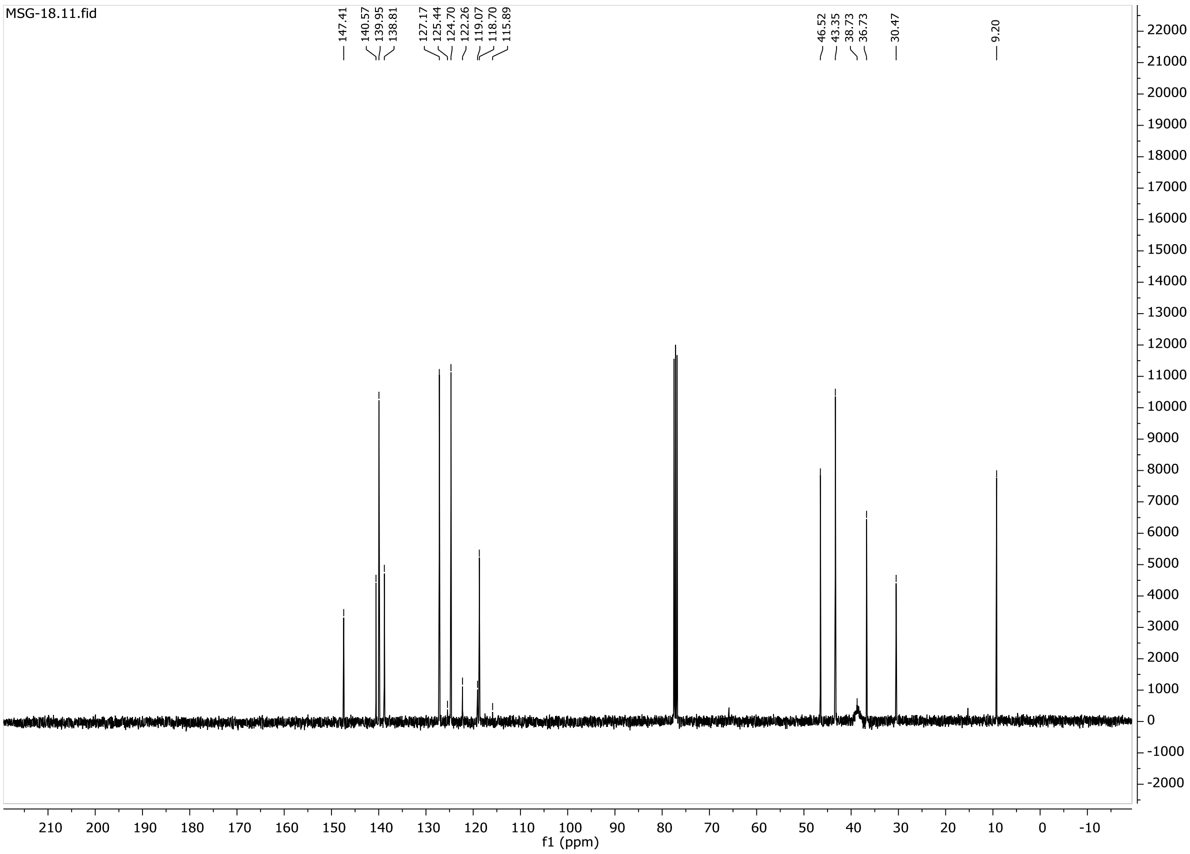
MSG-18.11.fid
147.41 140.57 139.95 138.81 127.17 125.44 124.70 122.26 119.07 118.70 115.89
46.52 43.35 38.73 36.73 30.47 9.20
f1 (ppm)

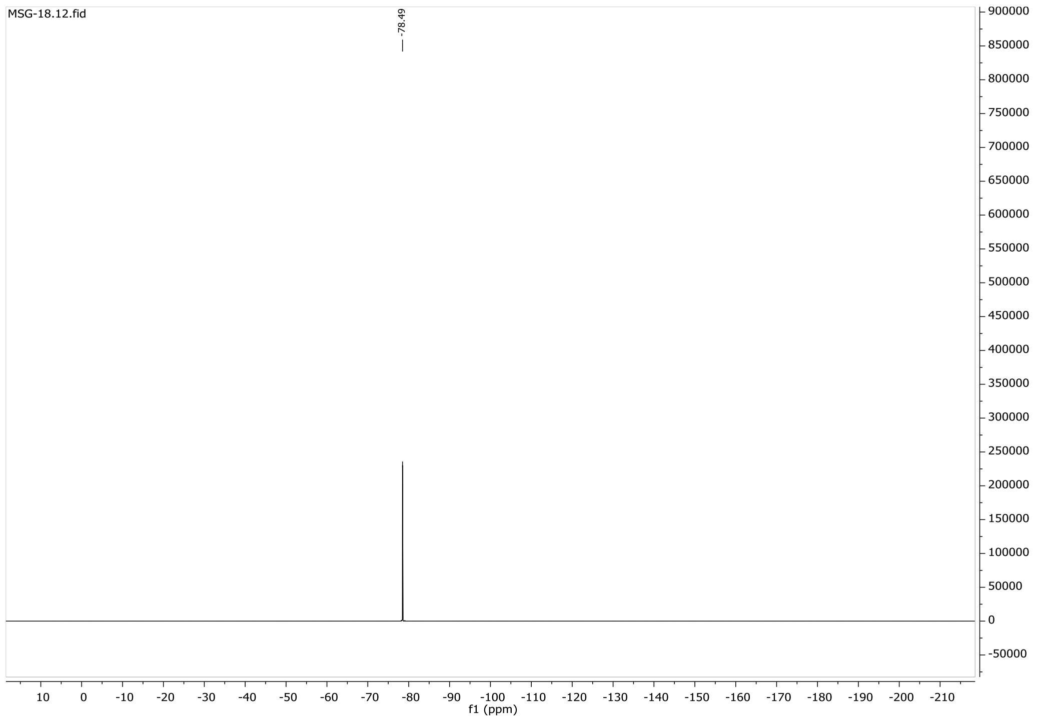

NOESY spectrum shows a correlation of the proton on position 15 with both N^+Me (22) and NMe_2 (20, 21). Correlation of protons on position 22 (N^+Me) with protons on 11 ($\underline{CH_2}CH_3$) and absence of correlation between protons on 20, 21 (NMe_2) and protons on 11 ($\underline{CH_2}CH_3$) is only possible with the 4-substitution pattern.

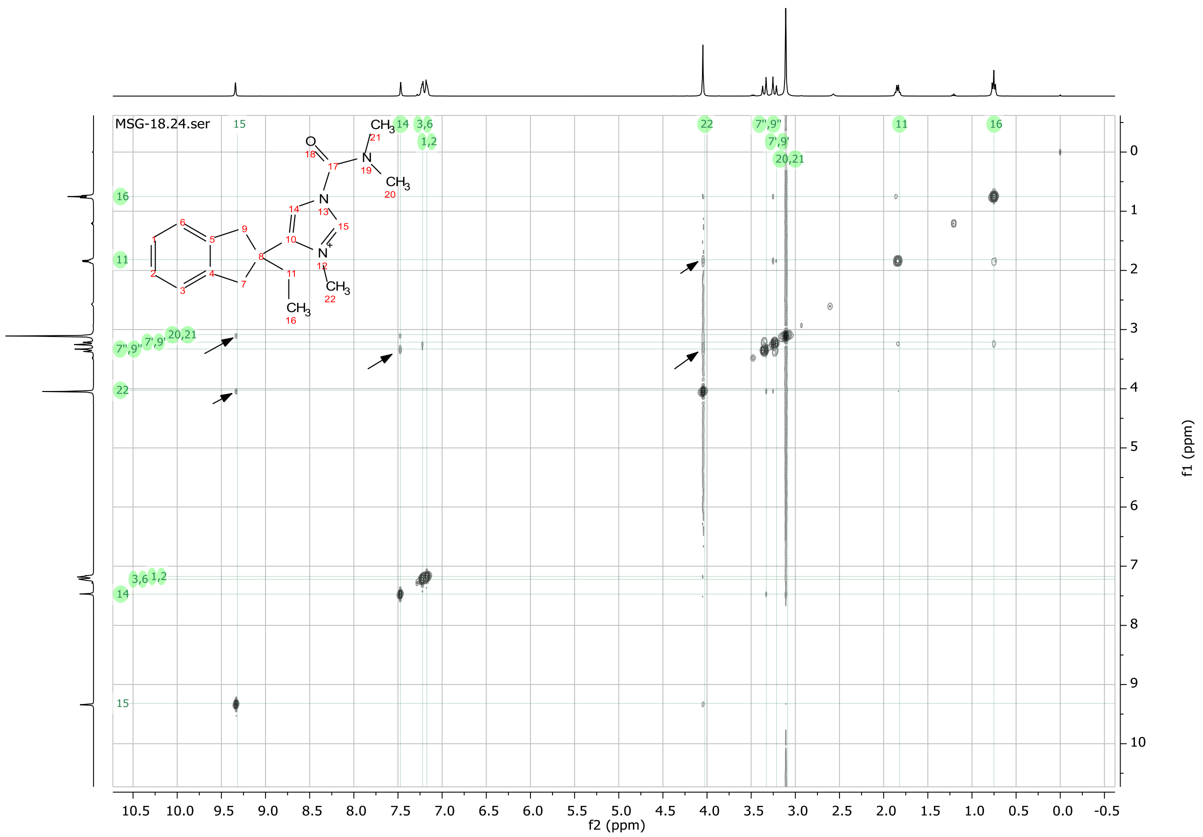

14

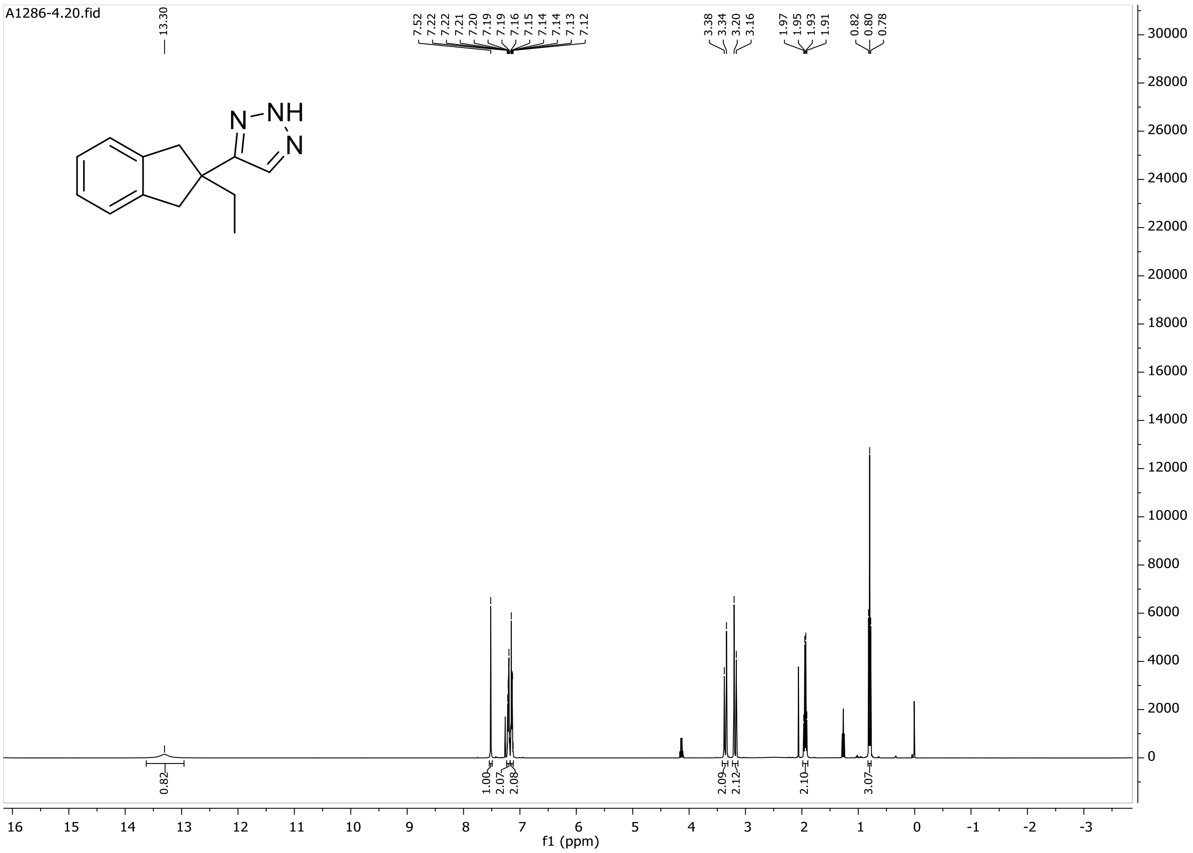
A1286-4.20.fid
13.30
7.52 7.22 7.22 7.21 7.20 7.19 7.19 7.16 7.15 7.14 7.14 7.13 7.12
3.38 3.34 3.20 3.16
1.97 1.95 1.93 1.91
0.82 0.80 0.78
N
NH
N
0.82
1.00 2.07 2.08
2.09 2.12
2.10
3.07
f1 (ppm)

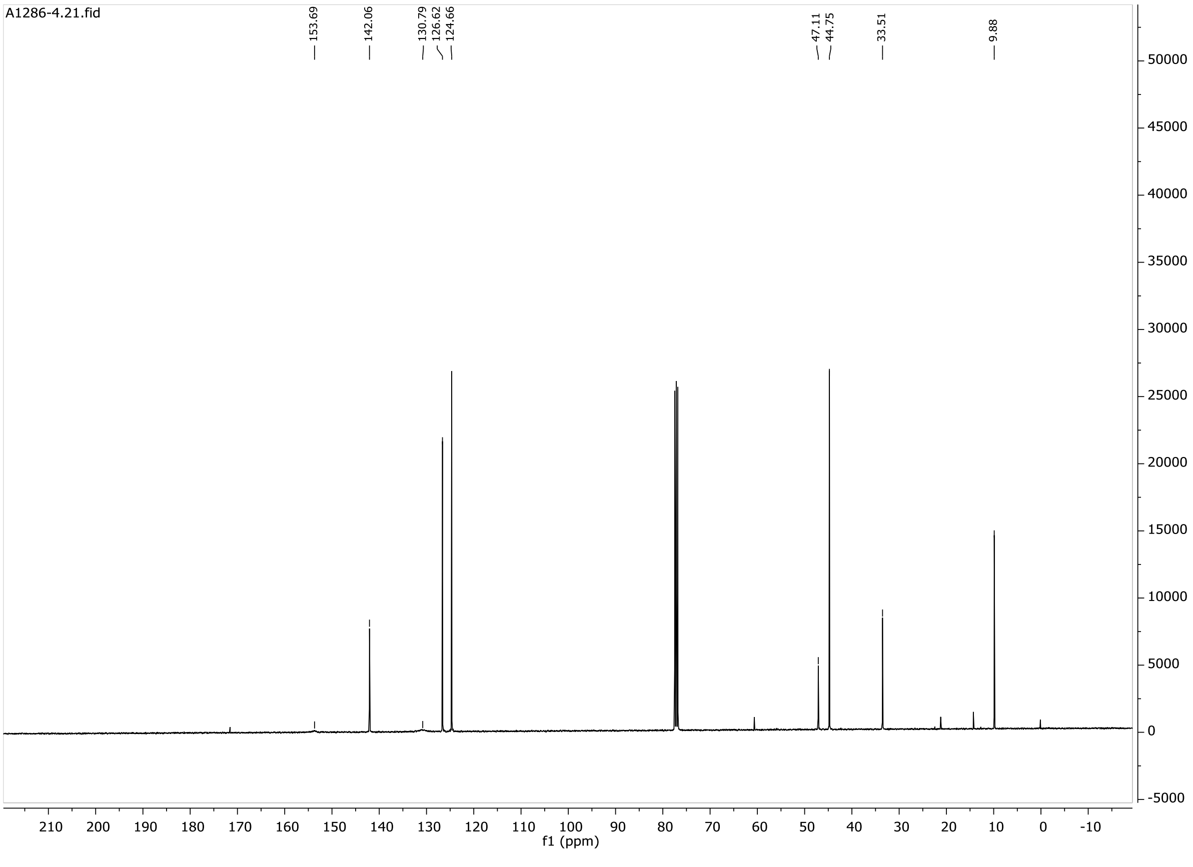
A1286-4.21.fid
153.69
142.06
130.79 126.62 124.66
47.11 44.75
33.51
9.88
f1 (ppm)

15

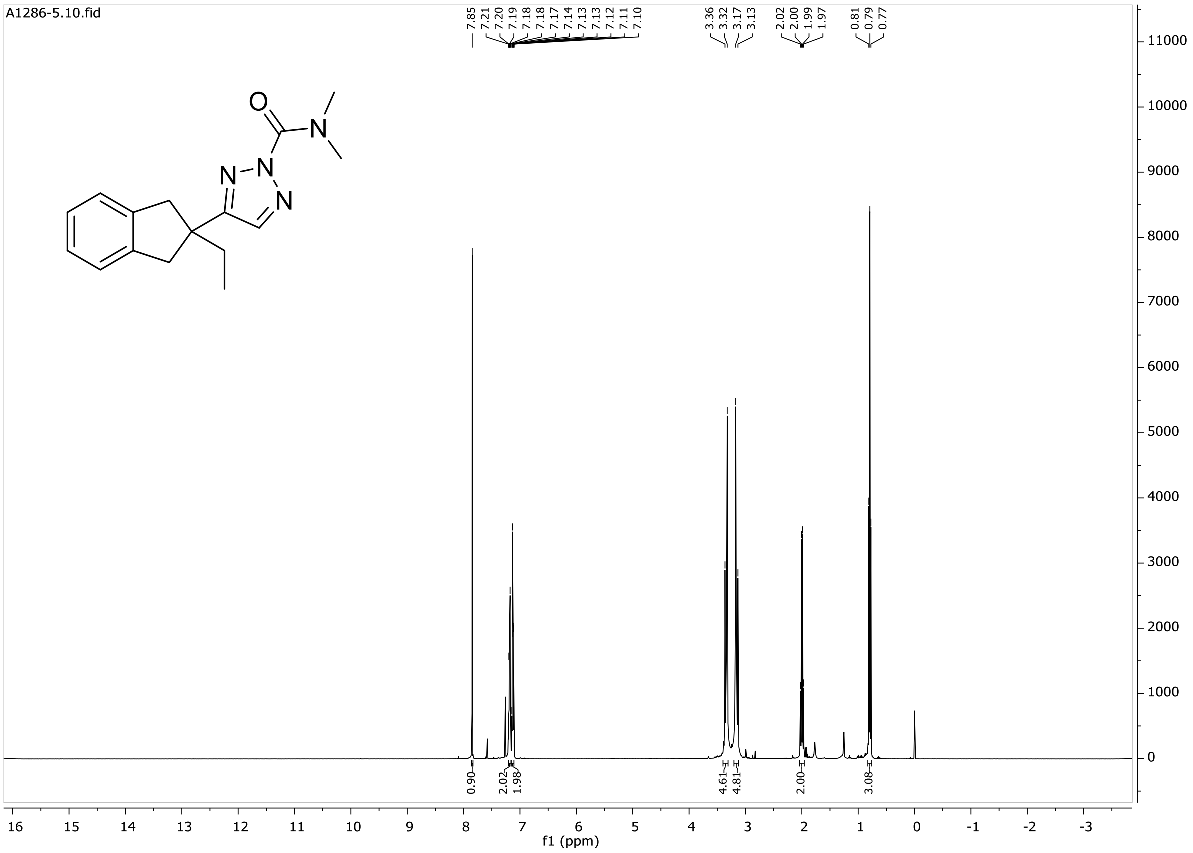
A1286-5.10.fid
7.85
7.21
7.20
7.19
7.18
7.17
7.14
7.13
7.13
7.12
7.11
7.10
3.36
3.32
3.17
3.13
2.02
2.00
1.99
1.97
0.81
0.79
0.77
0.90
2.02
1.98
4.61
4.81
2.00
3.08
f1 (ppm)

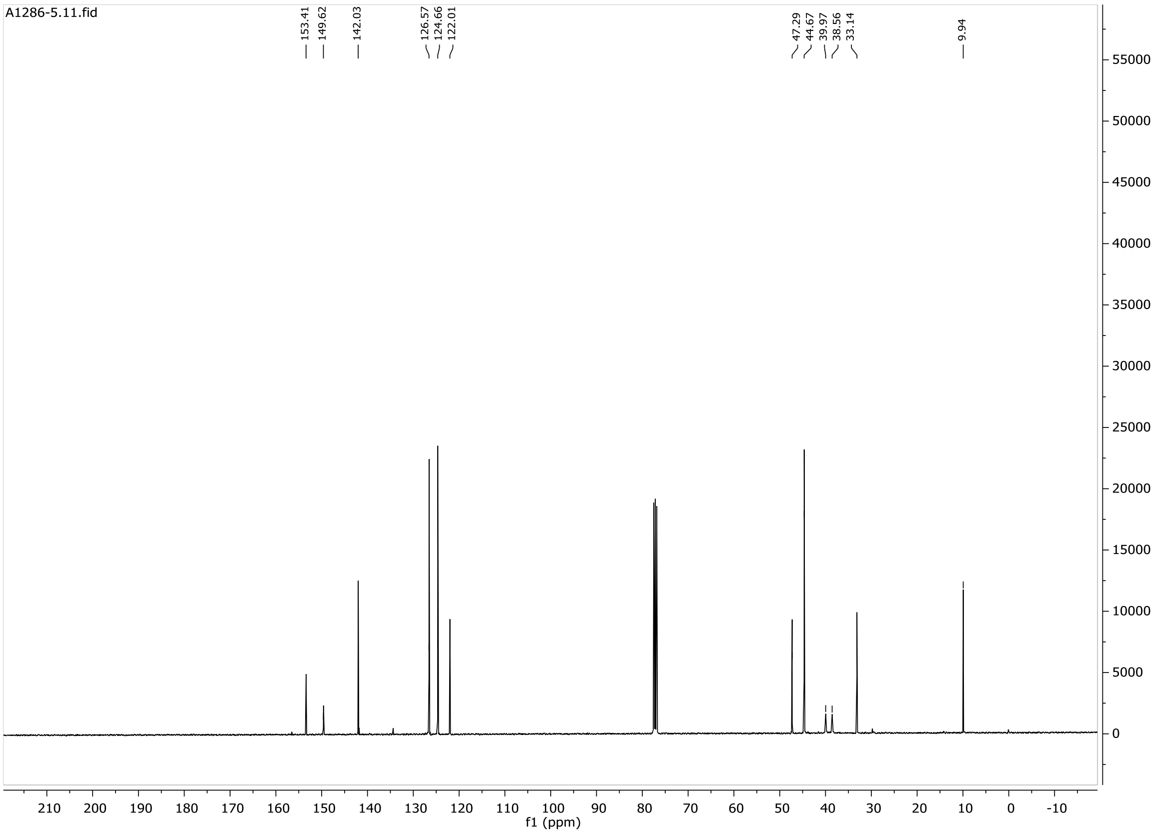
A1286-5.11.fid
153.41
149.62
142.03
126.57
124.66
122.01
47.29
44.67
39.97
38.56
33.14
9.94
f1 (ppm)

HSQC spectrum:

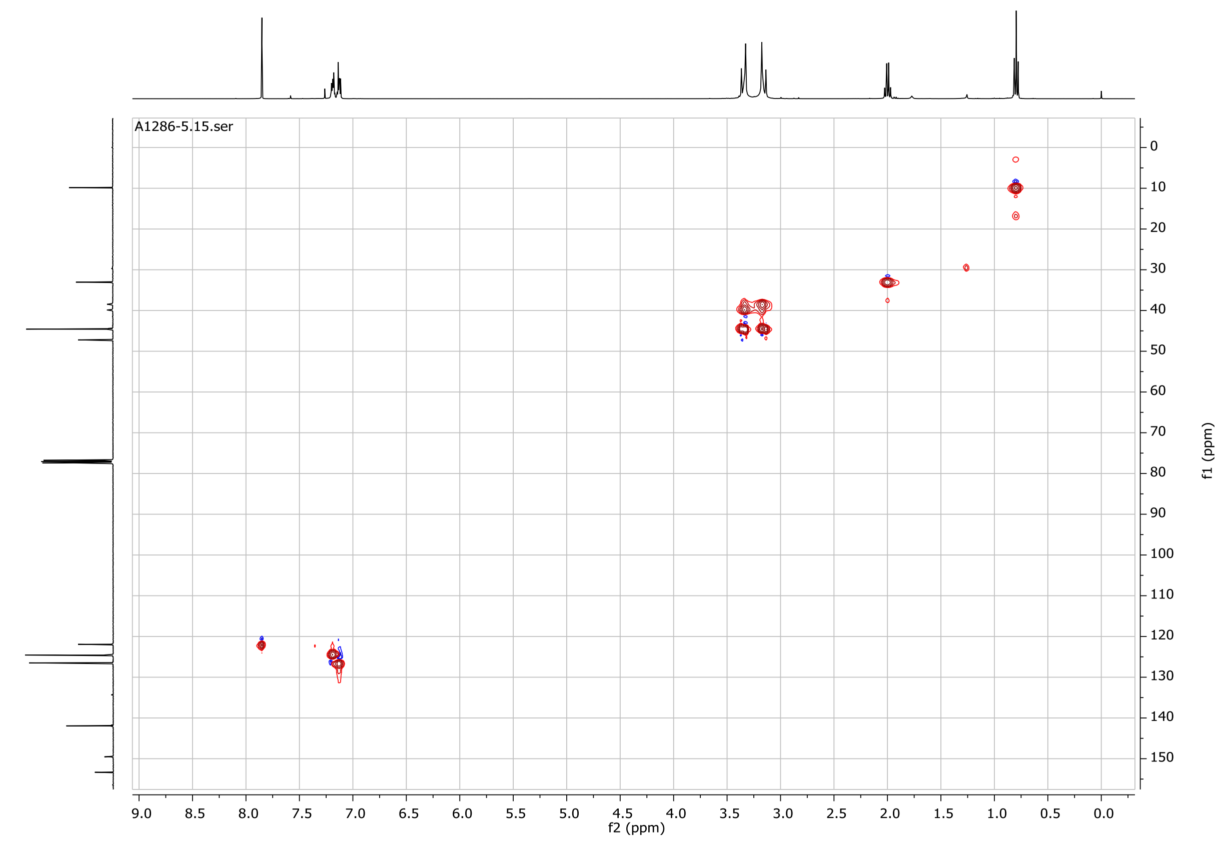

HMBC spectrum:

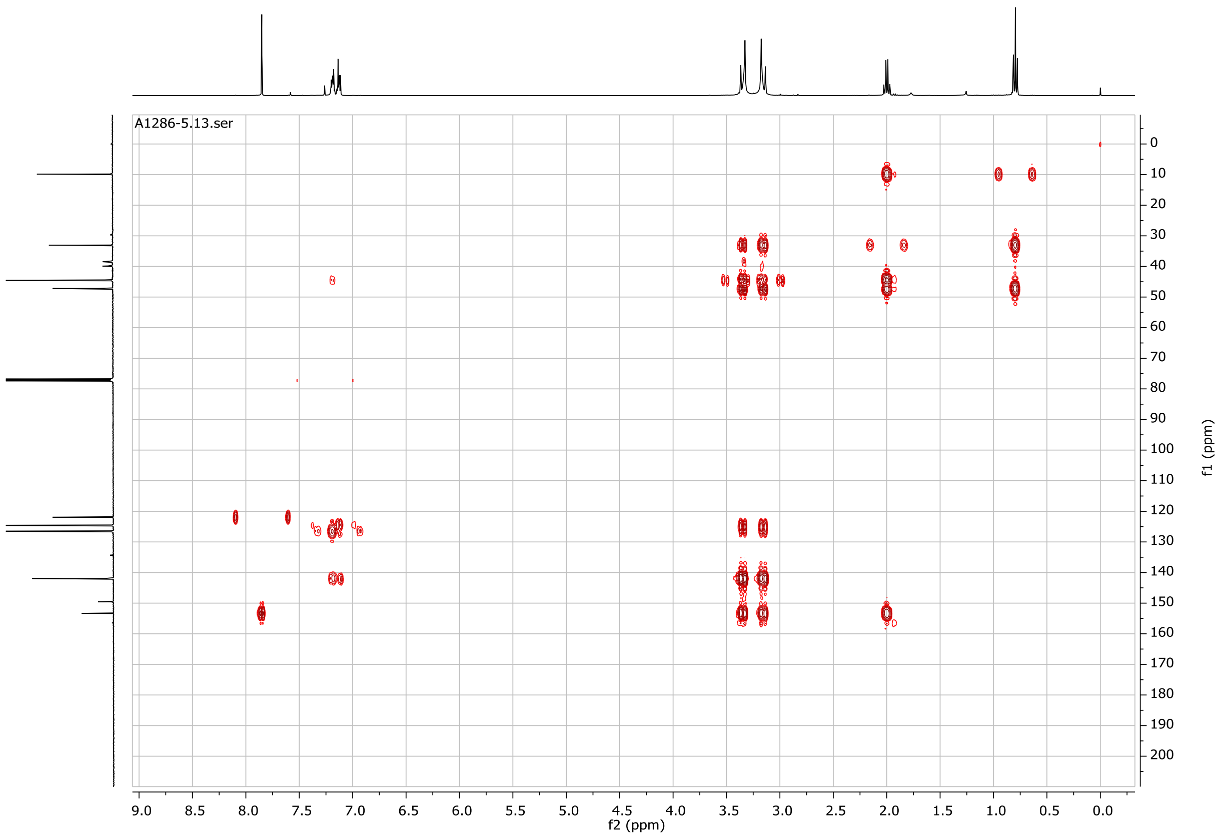

NOESY spectrum:

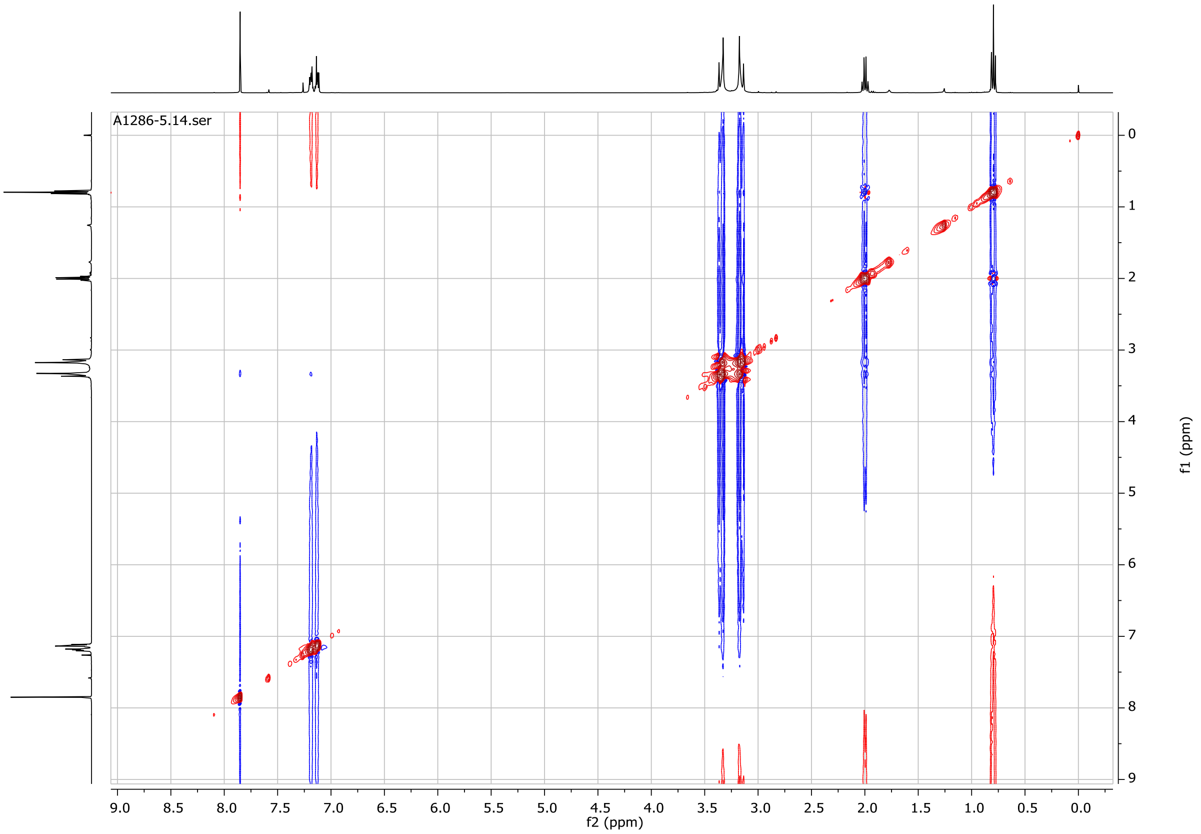

16

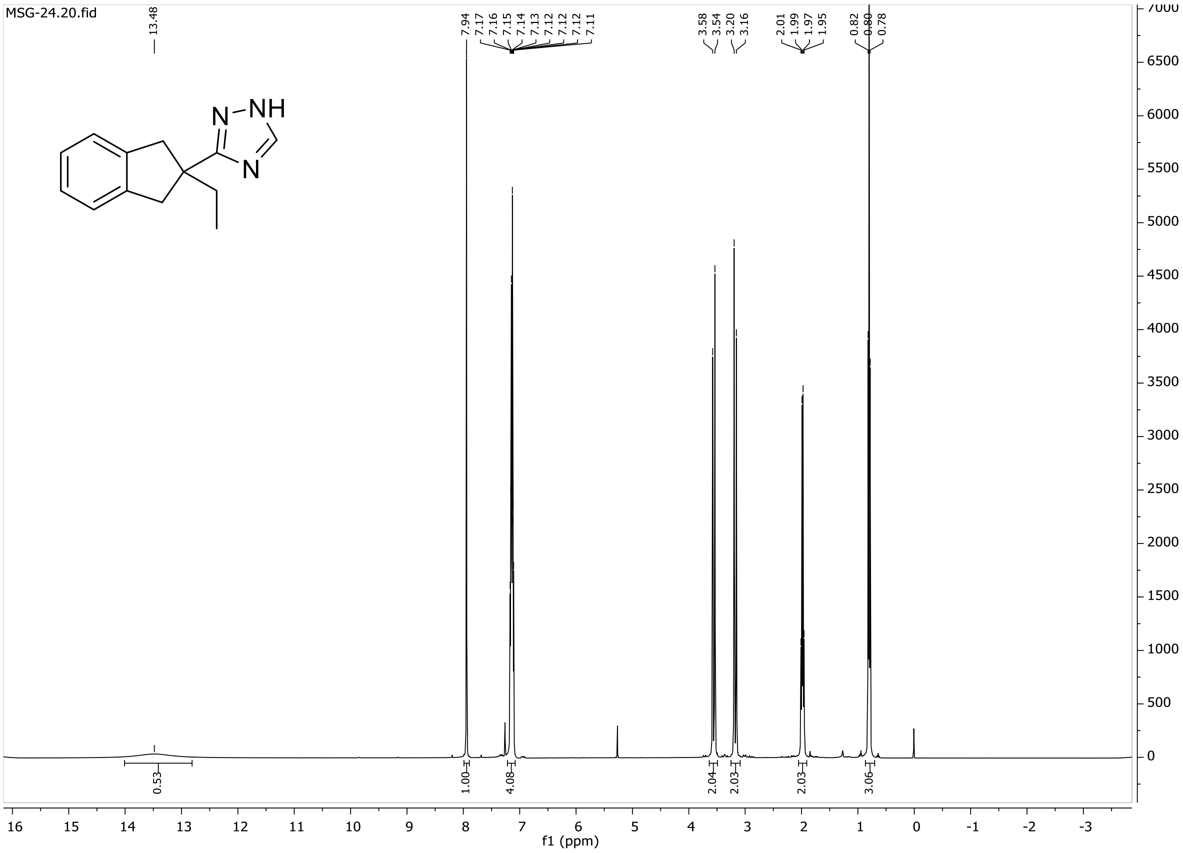

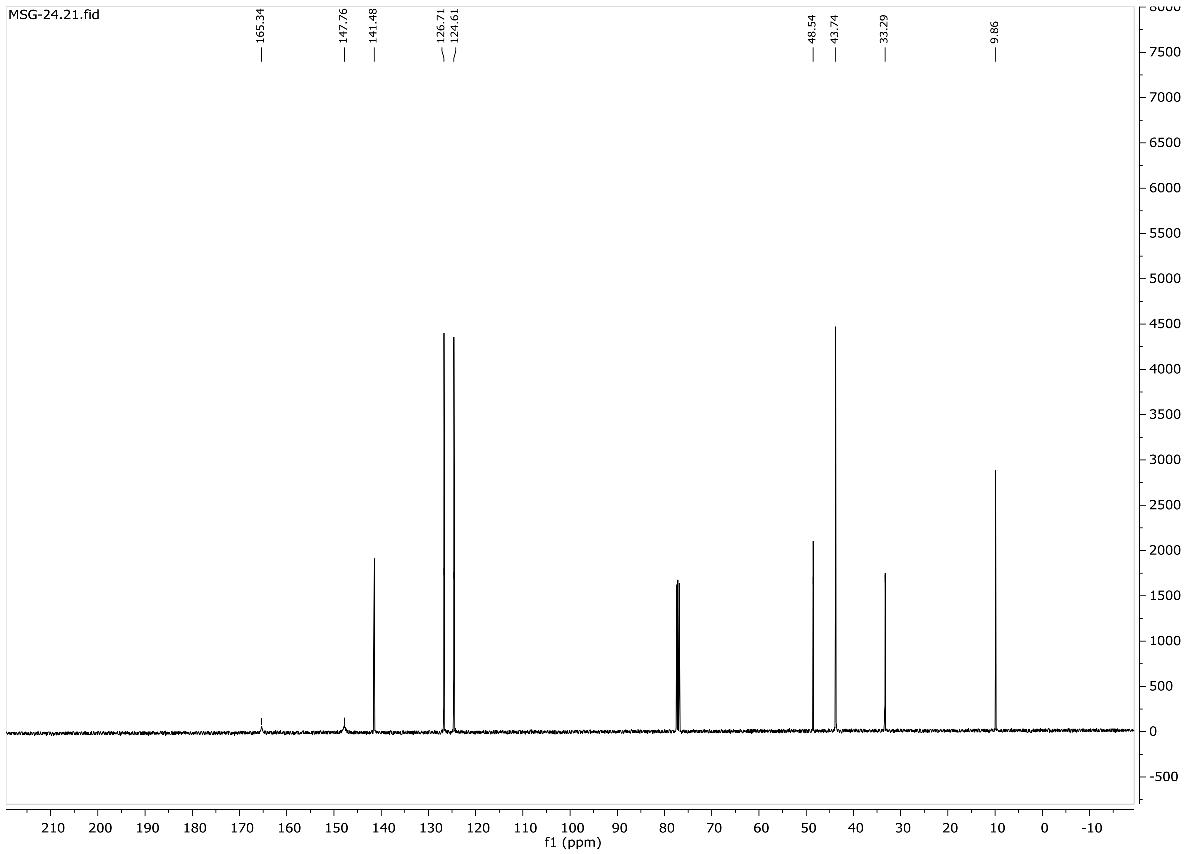
MSG-24.21.fid
165.34
147.76
141.48
126.71
124.61
48.54
43.74
33.29
9.86
f1 (ppm)

17

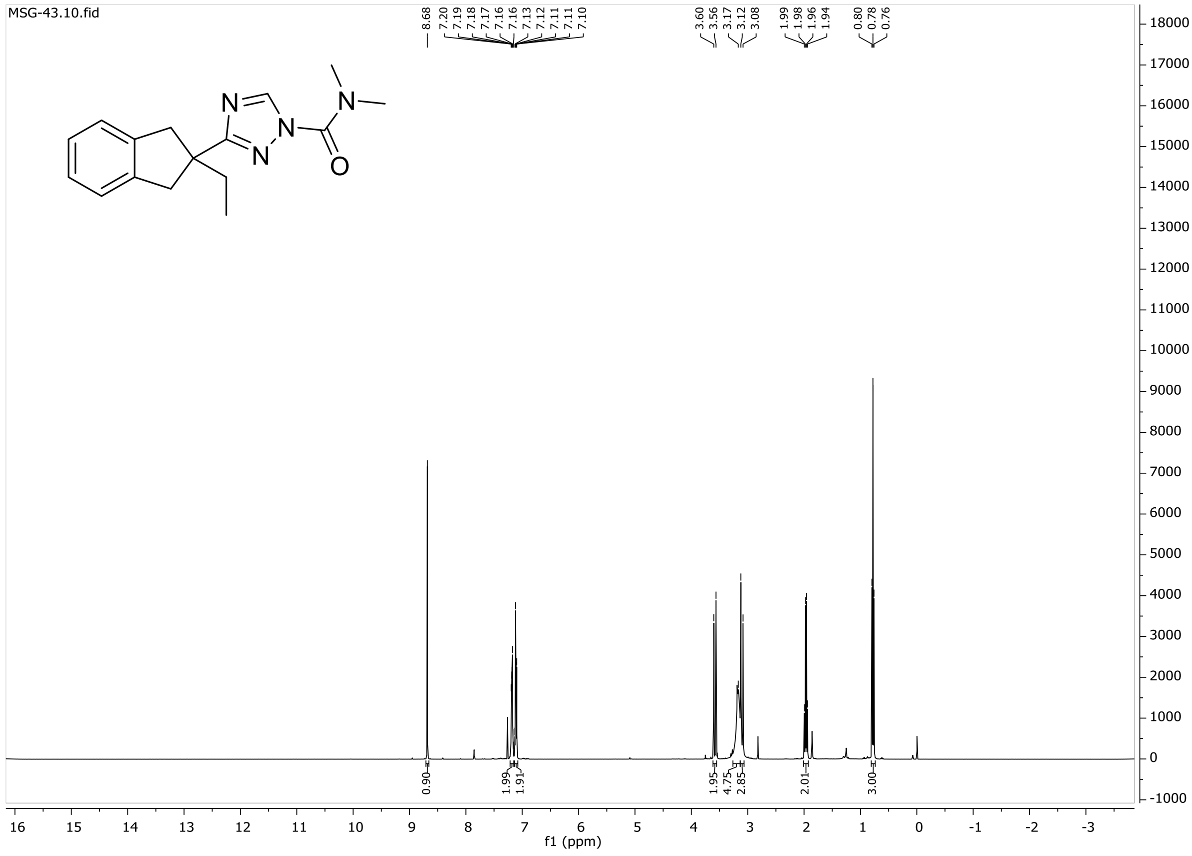
MSG-43.10.fid
8.68
7.20
7.19
7.18
7.17
7.16
7.13
7.12
7.11
7.11
7.10
3.60
3.56
3.17
3.12
3.08
1.99
1.98
1.96
1.94
0.80
0.78
0.76
N
N
N
N
O
0.90
1.99
1.91
1.95
4.75
2.85
2.01
3.00
f1 (ppm)

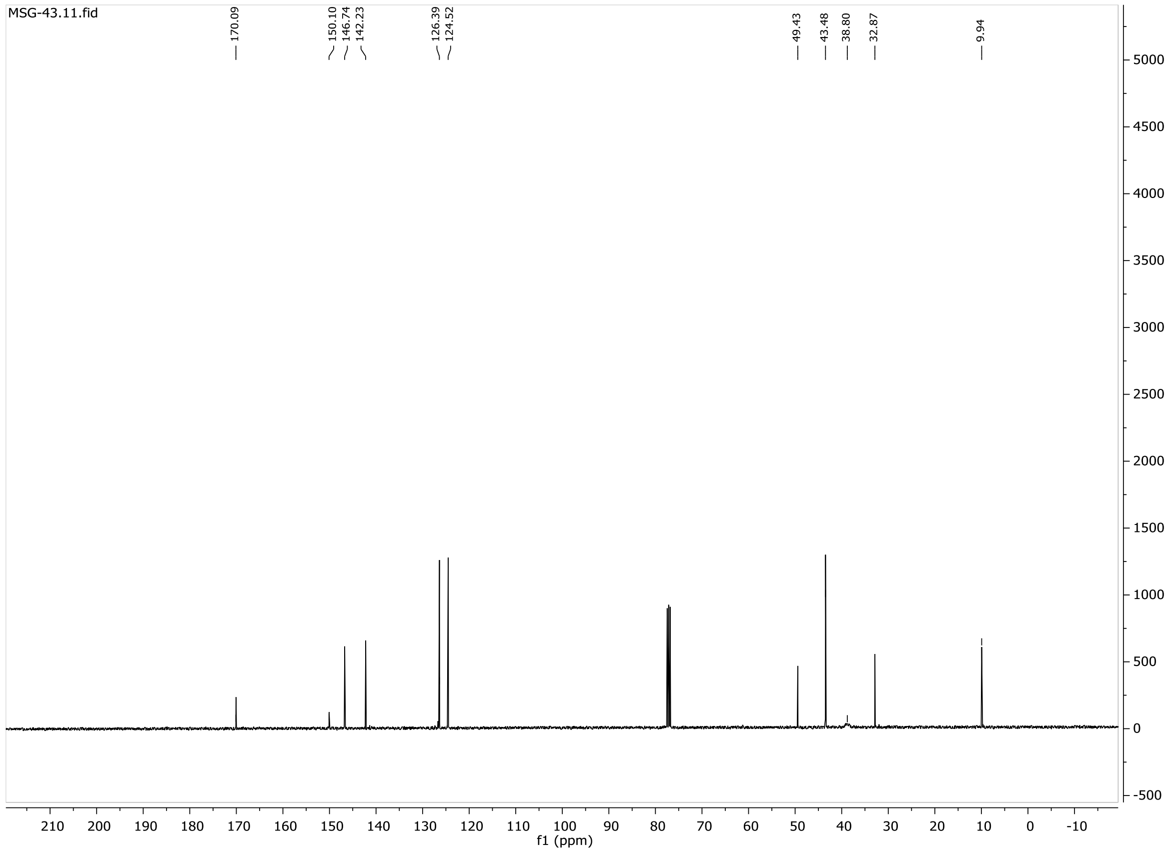

HSQC spectrum:

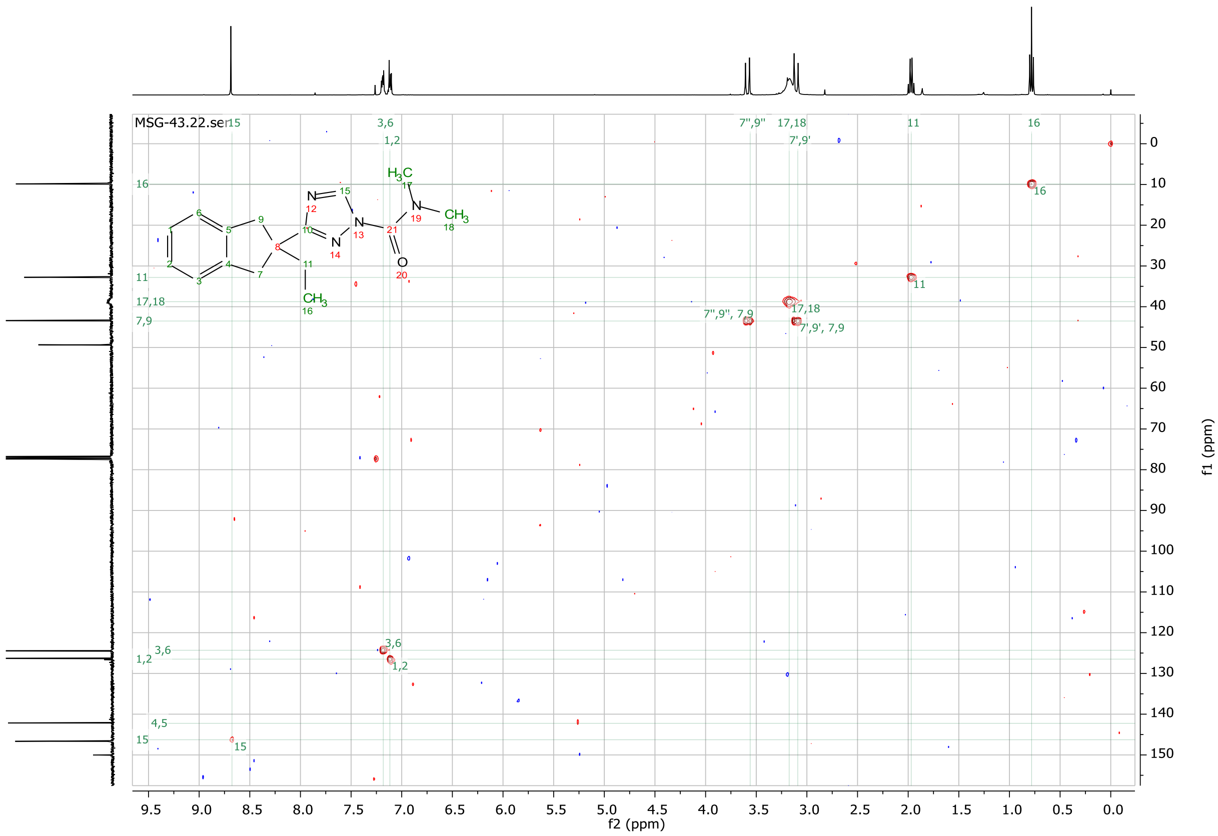

HMBC spectrum:

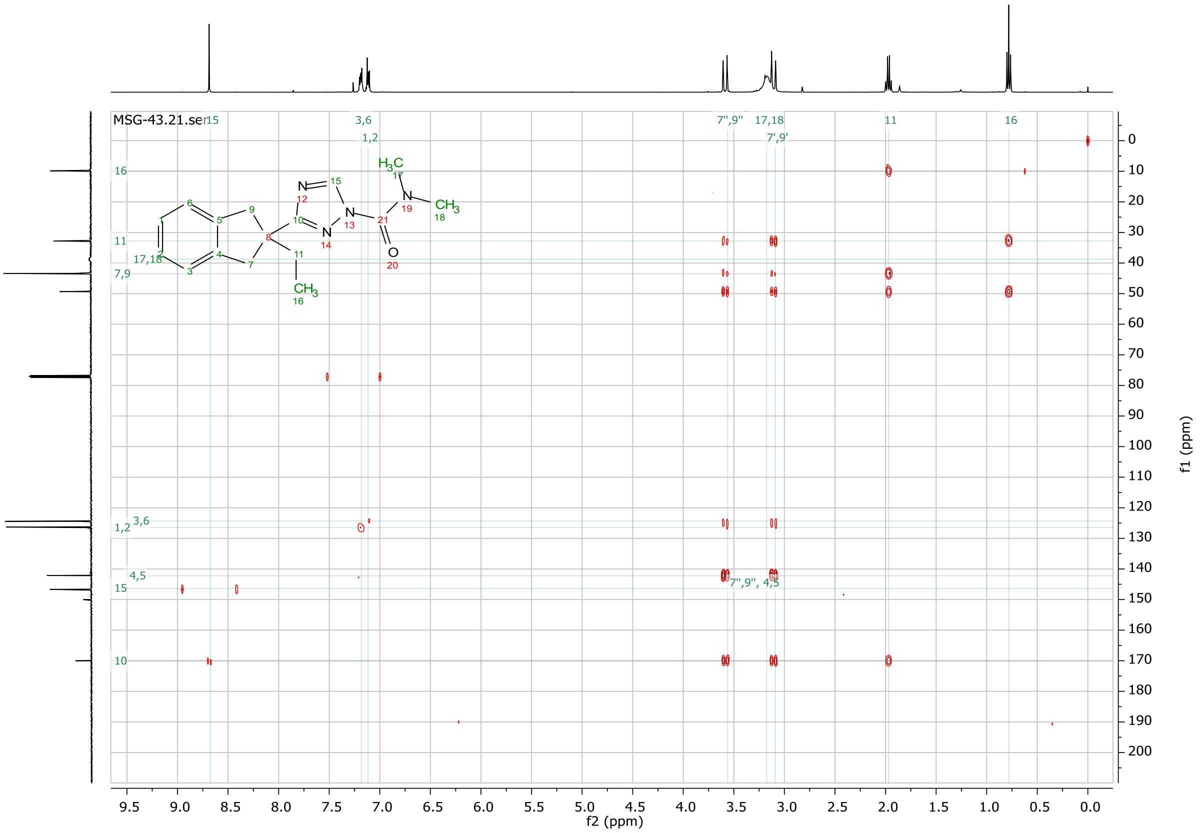

NOESY spectrum:

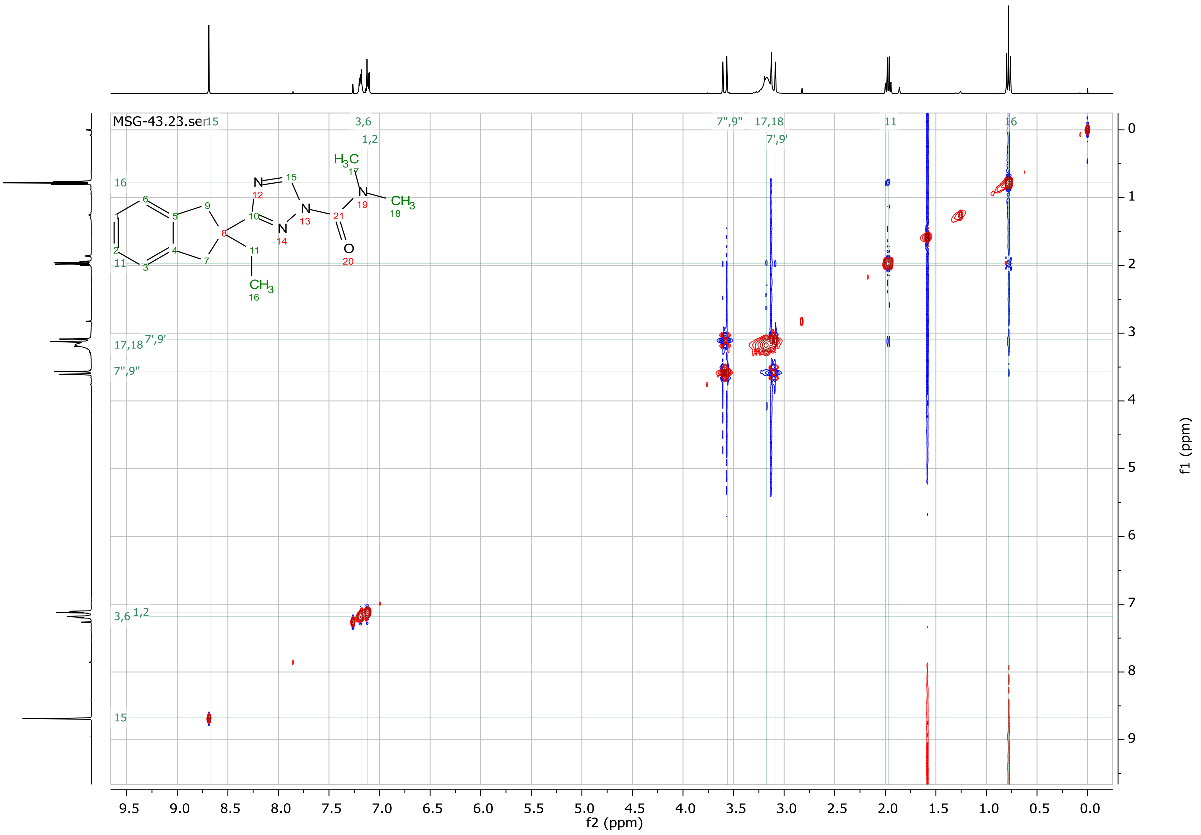

Supplement: Multimedia component 1 [file mmc1.pdf]
